# Adverse Effects Associated With Currently Commonly Used Antifungal Agents: A Network Meta-Analysis and Systematic Review

**DOI:** 10.3389/fphar.2021.697330

**Published:** 2021-10-29

**Authors:** Yan-Li Yang, Zi-Jian Xiang, Jing-Hua Yang, Wen-Jie Wang, Zhi-Chun Xu, Ruo-Lan Xiang

**Affiliations:** ^1^ Department of Pulmonary and Critical Care Medicine, Peking Union Medical College Hospital, Chinese Academy of Medical Sciences & Peking Union Medical College, Beijing, China; ^2^ Beijing Zhiyun Data Technology Co., Ltd., Beijing, China; ^3^ Department of Physiology and Pathophysiology, Peking University School of Basic Medical Sciences, Beijing, China

**Keywords:** adverse events, invasive fungal infections, antineoplastic agents, triazole, echinocandins, liposomal amphotericin B

## Abstract

**Background:** Invasive fungal infections (IFI) is an important contributing factor in morbidity and mortality of immunocompromised and critically ill patients. Although the therapeutic effects of these drugs on IFI have been well documented, the long-term use of antifungal agents has raised concerns about drug tolerability and treatment-related toxicity risks.

**Methods:** We searched articles published before June 30, 2020 in four electronic databases: Web of Science, Cochrane Library, embase and PubMed.

**Results:** 66 trials were determined to meet our inclusion criteria, providing data on 18,230 participants. We sorted out 23 AEs by system organ classes and six laboratory AEs, 13 of these were used to construct 13 network meta-analyses. Compared with LAmB, anidulafungin, caspofungin, micafungin, fluconazole, and posaconazole had a significantly low incidence of discontinuation of therapy due to AEs (OR = 0.24 (0.09,0.65), 0.24 (0.13,0.43), 0.32 (0.19,0.52), 0.38 (0.23,0.62) and 0.35 (0.17,0.69), respectively).

**Conclusion:** We found that echinocandins are the most tolerated antifungal agents with high safety. The AEs of triazole drugs are mainly concentrated on the increase in liver enzymes, nervous system disorders, especially visual disorders, gastrointestinal disorders, and cardiac diseases. LAmB is the least tolerated and has the most abundant AEs.

## Introduction

Invasive fungal infections (IFI) is an important contributing factor in morbidity and mortality of immunocompromised and critically ill patients (McNeil et al., 2001; Pfaller and Diekema, 2007). Over the past 25 years, the high incidence of IFI in humans is related to many factors (Calogiuri et al., 2019), including broad-spectrum antibiotics, antineoplastic agents, immunosuppressive agents, hyperalimentation, graft use and prosthetic devices (Lass-Flörl, 2009). In addition, with the improvement of medical care, the survival time of critically ill patients is prolonged, which makes them more vulnerable to IFI. At present, there are three kinds of drugs in the clinical treatment of IFI: azoles (mainly including itraconazole, fluconazole, posaconazole and voriconazole), echinocandins (such as anidulafungin, micafungin and caspofungin) and polyenes. Polyenes work by binding to ergosterol, a key structural component of fungal cell membranes, to form membrane pores. As a result, membrane permeability increases, causing leakage of potassium and other molecules in cells. Azoles destroy the stability of fungal cell membrane and reduces ergosterol production by inhibiting C-14a demethylation of lanosterol through binding to fungal cytochrome P-450 enzymes. Echinocandins disrupt glucan synthesis, the primary structural component of fungal cell walls by inhibition of the 1,3-b-D-glucan synthase enzyme (Bader et al., 2018), thus reduce the integrity of fungal cell walls. Although the therapeutic effects of these drugs on IFI have been well documented, the long-term use of antifungal agents has raised concerns about drug tolerability and treatment-related toxicity risks.

A large number of adverse reactions have been reported in many clinical studies in addition to the common hepatotoxicity, neurotoxicity, nausea and headache (Walsh et al., 2004; Maertens et al., 2016; Agarwal et al., 2018). In order to achieve good therapeutic effect, clinicians must evaluate and control the adverse reactions well. The objective of this study is to analyze the main adverse events (AEs) of currently commonly used antifungal agents, such as liposomal amphotericin B (LAmB), anidulafungin, caspofungin, micafungin, fluconazole, isavuconazole, itraconazole, posaconazole and voriconazole. Although direct randomized comparison is the most reliable way of comparing treatments, as the number of available treatments increases the number of possible pairwise comparisons increases quadratically, so it is common for only a small fraction of the possible comparisons to be performed. Therefore, when direct and indirect comparison results of the safety of these 9 antifungal agents were simultaneously available, we used network meta-analysis to analyze the comparison between multiple interventions based on indirect results or the combination of indirect results and direct results.

## Methods

### Protocol and Guidance

This systematic review and meta-analysis followed the Preferred Reporting Items for Systematic Reviews and Meta analyses (PRISMA) guidelines (Page et al., 2021). No review protocol or registration details are available.

### Eligibility Criteria

We enrolled Randomized Controlled Trials (RCTs) on the basis of PICOS principles, in which patients were treated with invasive fungal infection therapy or prophylactic/empiric antifungal therapy and reported toxicity or adverse events, with the intervention of one of the 9 antifungal agents, and the control group was placebo or one of the other 9 antifungal agents.

### Search Strategy and Screening

We searched articles published before June 30, 2020 in four electronic databases: Web of Science, Cochrane Library, embase and PubMed. Search terms were developed using a combination of MeSH/EMTREE terms and free-text terms to capture the relevant population, outcomes, and study type according to the populations, interventions, comparisons, outcomes, and study types. The following search terms were used in the search queries ((antifungal) OR (invasive fungal infections) OR (aspergillosis) OR (invasive mould disease) OR (paracoccidioidomycosis) OR (cryptococcosis) OR (zygomycosis) OR (Mucormycosis)) AND ((Anidulafungin) OR (Micafungin) OR (caspofungin) OR (fluconazole) OR (voriconazole) OR (itraconazole) OR (posaconazole) OR (Isavuconazole) OR (antifungal agents [MeSH Terms])). The titles and abstracts of the studies were screened independently by two methodologically competent reviewers to determine whether the cited articles met the eligibility criteria. Only after they reach an agreement over differences through consensus discussion, or arbitration by a third reviewer, can they read the full text and extract relevant data. The reasons for inclusion or exclusion were documented in detail. Non-English studies, case reports, letters and minutes of meetings were not included. The PRISMA flow diagram was used to summarize study selection processes.

### Data Extraction and Efficacy Measures

Two investigators initially used a predefined data extraction sheet to independently perform data extraction from each included study, such as antifungal mode, drugs, dose duration, AEs, sample size, patients, age, male%, grouping and number of patients in the group, authors, publication year, country, study design. The third investigator independently verified the data to ensure accuracy. If no data in digital format were available, we estimated data from the graphs using the free software Plot Digitizer. The outcomes measurements of this meta-analysis include: first, the odds ratio of the incidence of therapy discontinuation between the antifungal agents and placebo or between two antifungal agents in pairwise comparisons; and second, the odds ratio of the incidence of AEs. The adverse events by system organ classes and laboratory adverse events are listed in Table 1 (Maertens et al., 2016). We selected AEs that were elaborated in most studies to perform this network meta-analysis, and those mentioned in no more than 10 studies were not selected. We also conducted two subgroup analyses of empirical/definitive therapy and prophylactic therapy to compare the incidence of AEs.

**TABLE 1:** The antifungal agents involved in each AE, and the number of studies included in each AE.

AEs	Treatment	Studies included
Withdrawal from study medication due to adverse events	**Total**	57
	Placebo	14
	LAmB	18
	Anidulafungin	2
	Caspofungin	9
	Fluconazole	23
	Isavuconazole	2
	Itraconazole	18
	Micafungin	11
	Posaconazole	4
	Voriconazole	13
Blood and lymphatic system disorders	**Total**	9
	Placebo	4
	LAmB	3
	Fluconazole	3
	Isavuconazole	1
	Itraconazole	2
	Micafungin	3
	Voriconazole	2
Cardiac disorders	**Total**	17
	Placebo	1
	LAmB	8
	Caspofungin	3
	Fluconazole	4
	Isavuconazole	2
	Itraconazole	6
	Micafungin	5
	Posaconazole	1
	Voriconazole	5
Congenital, familial, and genetic disorders	**Total**	1
	Isavuconazole	1
	Voriconazole	1
Ear and labyrinth disorders	**Total**	1
	Isavuconazole	1
	Voriconazole	1
Endocrine disorders	**Total**	3
	Placebo	1
	LAmB	1
	Caspofungin	1
	Fluconazole	1
	Itraconazole	1
	Micafungin	1
Gastrointestinal disorders	**Total**	51
	Placebo	13
	LAmB	18
	Anidulafungin	2
	Caspofungin	9
	Fluconazole	21
	Isavuconazole	1
	Itraconazole	16
	Micafungin	8
	Posaconazole	4
	Voriconazole	11
General disorders and administrative site conditions	**Total**	46
	Placebo	12
	LAmB	18
	Anidulafungin	1
	Caspofungin	9
	Fluconazole	19
	Isavuconazole	2
	Itraconazole	12
	Micafungin	7
	Posaconazole	3
	Voriconazole	10
Hepatobiliary disorders	**Total**	23
	Placebo	3
	LAmB	5
	Caspofungin	2
	Fluconazole	10
	Isavuconazole	1
	Itraconazole	8
	Micafungin	9
	Posaconazole	2
	Voriconazole	7
Immune system disorders	**Total**	8
	Placebo	1
	LAmB	4
	Caspofungin	1
	Fluconazole	3
	Isavuconazole	1
	Itraconazole	2
	Micafungin	2
	Voriconazole	2
Infections and infestations	**Total**	8
	Placebo	1
	LAmB	2
	Caspofungin	1
	Fluconazole	4
	Isavuconazole	1
	Itraconazole	3
	Micafungin	1
	Posaconazole	1
	Voriconazole	2
Injury, poisoning, and procedural complications	**Total**	1
	Isavuconazole	1
	Voriconazole	1
Investigations (abnormal laboratory tests)	Total	1
	Isavuconazole	1
	Voriconazole	1
Metabolism and nutrition disorders	**Total**	7
	Placebo	1
	LAmB	3
	Fluconazole	2
	Isavuconazole	1
	Itraconazole	2
	Micafungin	2
	Voriconazole	3
Musculoskeletal and connective tissue disorders	**Total**	3
	Placebo	1
	LAmB	1
	Caspofungin	1
	Fluconazole	1
	Isavuconazole	1
	Voriconazole	1
Neoplasms benign, malignant and unspecifi ed	**Total**	1
	Isavuconazole	1
	Voriconazole	1
Psychiatric disorders	**Total**	4
	Placebo	1
	Fluconazole	1
	Isavuconazole	1
	Itraconazole	1
	Micafungin	2
	Voriconazole	2
Renal and urinary disorders	**Total**	16
	Placebo	1
	LAmB	11
	Caspofungin	3
	Fluconazole	5
	Isavuconazole	1
	Itraconazole	4
	Micafungin	2
	Voriconazole	5
Reproductive system and breast disorders	**Total**	2
	Placebo	1
	Fluconazole	1
	Isavuconazole	1
	Voriconazole	1
Respiratory, thoracic, and mediastinal disorders	**Total**	15
	Placebo	4
	LAmB	9
	Caspofungin	3
	Fluconazole	5
	Isavuconazole	1
	Itraconazole	3
	Micafungin	1
	Voriconazole	4
Skin and subcutaneous tissue disorders	**Total**	42
	Placebo	12
	LAmB	16
	Caspofungin	9
	Fluconazole	18
	Isavuconazole	1
	Itraconazole	12
	Micafungin	5
	Posaconazole	1
	Voriconazole	10
Social circumstances	**Total**	1
	Isavuconazole	1
	Voriconazole	1
Vascular disorders	**Total**	11
	Placebo	1
	LAmB	4
	Anidulafungin	2
	Caspofungin	5
	Fluconazole	5
	Micafungin	3
	Voriconazole	3
Nervous system disorders	**Total**	42
	Placebo	10
	LAmB	10
	Anidulafungin	3
	Caspofungin	8
	Fluconazole	16
	Isavuconazole	1
	Itraconazole	10
	Micafungin	7
	Posaconazole	4
	Voriconazole	16
Increase in liver enzymes	**Total**	29
	Placebo	6
	LAmB	11
	Anidulafungin	1
	Caspofungin	6
	Fluconazole	10
	Itraconazole	11
	Micafungin	6
	Posaconazole	3
	Voriconazole	5
Decrease in potassium	**Total**	23
	Placebo	1
	LAmB	17
	Caspofungin	7
	Fluconazole	8
	Itraconazole	6
	Micafungin	4
	Voriconazole	3
Increase in total or direct bilirubin	**Total**	14
	LAmB	8
	Caspofungin	4
	Fluconazole	5
	Itraconazole	6
	Micafungin	2
	Posaconazole	2
	Voriconazole	2
Decrease in magnesium	**Total**	8
	Placebo	1
	LAmB	7
	Caspofungin	2
	Fluconazole	2
	Itraconazole	2
	Micafungin	1
	Voriconazole	1
Increase in blood urea nitrogen	**Total**	2
	LAmB	2
	Caspofungin	2
Increase in creatinine	**Total**	11
	Placebo	1
	LAmB	8
	Caspofungin	4
	Fluconazole	1
	Itraconazole	3
	Micafungin	2
	Voriconazole	3

AE, adverse event; LAmB, liposomal amphotericin B.

### Statistical Analysis

Random effects network meta-analysis was used for the comparison of mixed multiple treatments, which uses a frequentist framework of Stata 14 network package. We presented the summary odds ratios (ORs) with their 95% CIs in league tables. Using the distribution of the ranking probabilities and the surface under the cumulative ranking curves (SUCRAs), the relative rankings of the different antifungal agents for each AE were estimated; Inconsistency in the random effect model was assessed by Q statistic that was calculated based on design-by-treatment model (Jackson et al., 2014). We evaluated the small-study effects by visually observing publication bias by using comparison-adjusted funnel plot (Chaimani et al., 2013).

### Assessment of Risk of Bias in Individual Studies

The quality of the retrieved RCTs was assessed according to the Cochrane Handbook of Systematic Reviews of Interventions (Higgins et al., 2011). The risk of bias assessment included the following domains: sequence generation (selection bias), allocation sequence concealment (selection bias), blinding of participants and personnel (performance bias), blinding of outcome assessment (detection bias), incomplete outcome data (attrition bias), and selective outcome reporting (reporting bias) and other potential sources of bias. Studies were graded as high risk, low risk or uncertain risk.

## Results

### Study Selection

**FIGURE 1:**
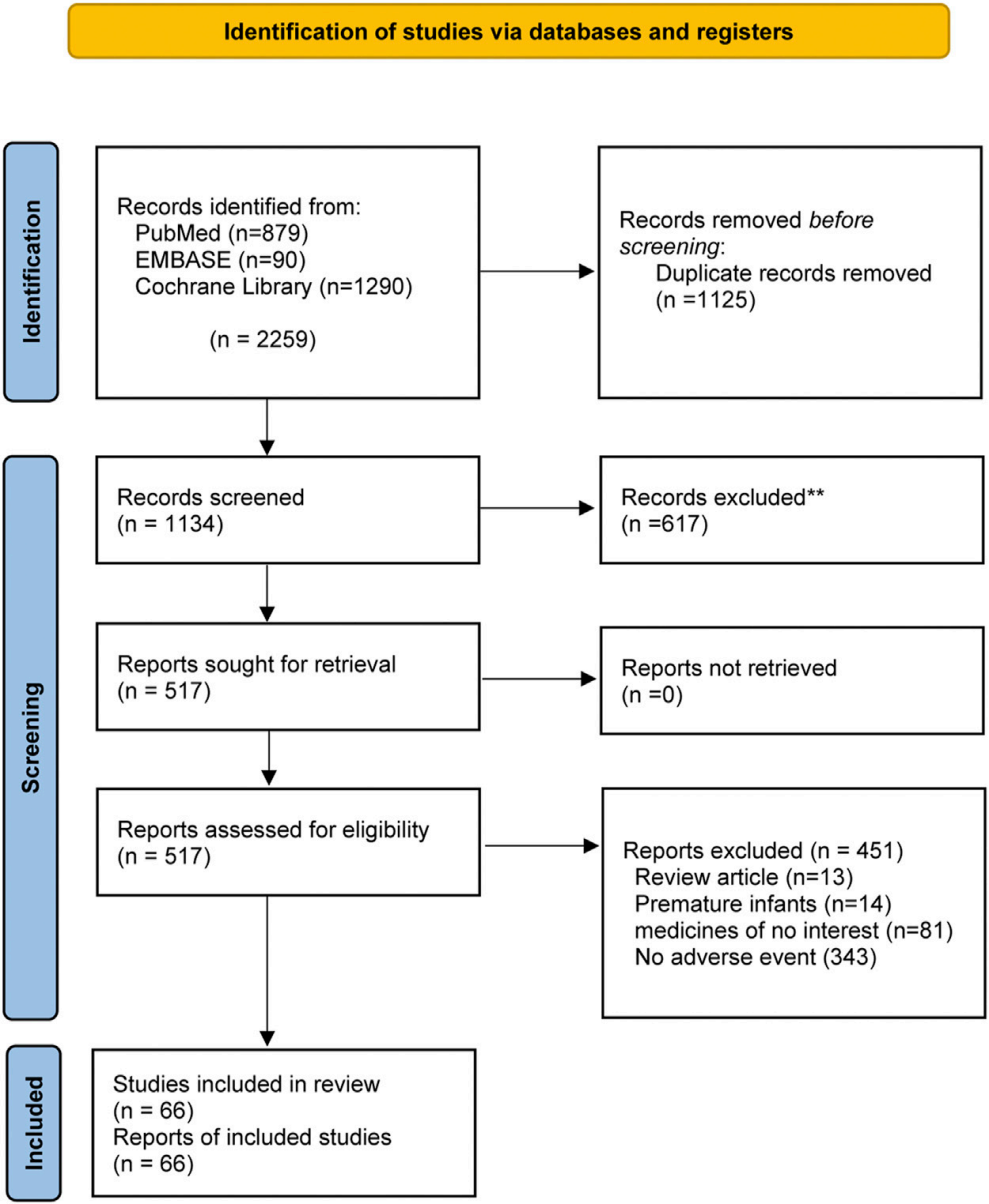
Flowchart of study selection.

We identified 1,134 studies through an initial electronic search. Of these, 617 were excluded after reading the title and abstract, with the remaining 517 studies to be further evaluated. After reading the full text, 66 trials were determined to meet our inclusion criteria, providing data on 18,230 participants (Figure 1).

### Study Characteristics and Risk of Bias Within Studies

Table 1 summarizes the 66 RCTs that were published between 1996 and 2019, of which 55 trials reported withdrawal study medication due to AEs, 39 studies reported laboratory AEs, and all 66 studies reported clinical AEs. The detailed information of each study was listed in Supplementary Table S1.

Thirty-three studies were double-blinded RCTs. One included study was prospective non-randomized, open-label trial, 31 studies were open-label, while one study was single-blinded, these studies had a high risk of performance bias. Seven studies had a high risk of attrition bias, and most studies had a low risk of reporting bias. Details on quality assessment were illustrated in Supplementary Figures S1, S2.

### Synthesis of Results

Description of the process of network meta-analysis, Supplementary Result S1.

### Tolerability Analysis

**FIGURE 2:**
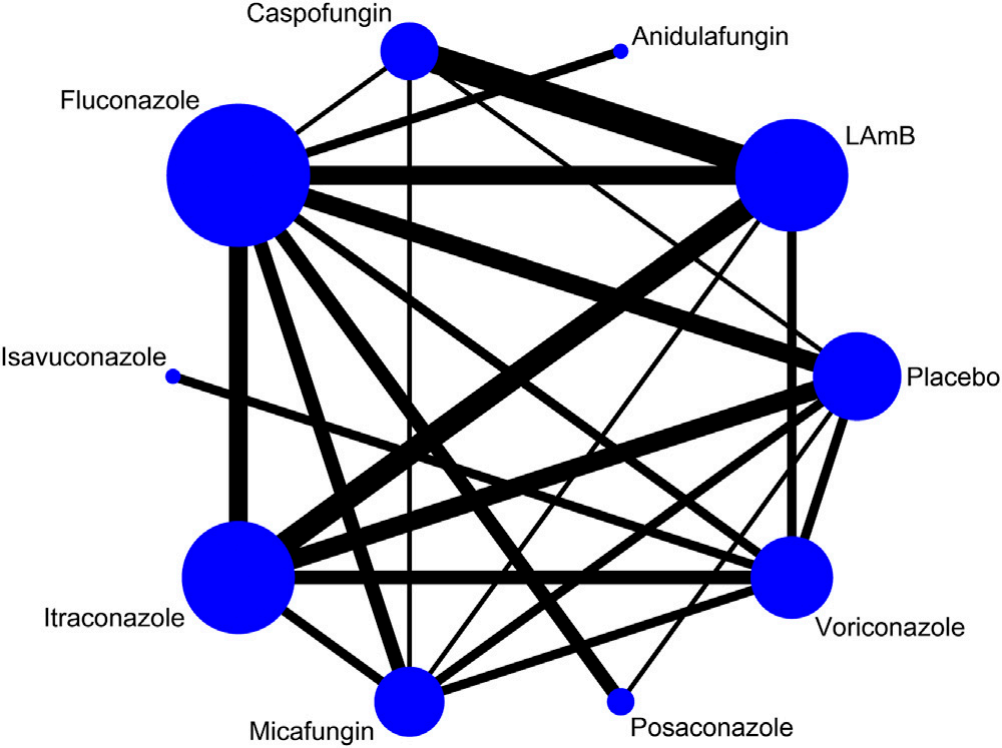
Network plot for tolerability analysis.

Nine antifungal agents were reported in 57 tolerability studies, network plot see Figure 2. Compared with placebo, LAmB, itraconazole and voriconazole had a significantly high incidence of discontinuation of therapy due to AEs (OR = 3.20 (1.81,5.66), 2.39 (1.41,4.05) and 2.50 (1.30,4.81), respectively). Compared with LAmB, anidulafungin, caspofungin, micafungin, fluconazole, and posaconazole had a significantly low incidence of discontinuation of therapy due to AEs (OR = 0.24 (0.09,0.65), 0.24 (0.13,0.43), 0.32 (0.19,0.52), 0.38 (0.23,0.62) and 0.35 (0.17,0.69), respectively), Supplementary Table S2.1. In SUCRA ranking, caspofungin was the best and LAmB was the worst. Table 2.

**TABLE 2:** According to SUCRA, the best (with the lowest side effect rate) and the worst (with the highest side effect rate) antifungal agents.

AEs	Best	Worst
Withdrawal from study medication due to adverse events	Caspofungin	LAmB
Increase in creatinine	Caspofungin	Fluconazole
Increase in total or direct bilirubin	Caspofungin	Micafungin
Decrease in potassium	Fluconazole	Placebo
Increase in liver enzymes	Anidulafungin	Posaconazole
Nervous system disorders	Posaconazole	Isavuconazole
Vascular disorders	Placebo	LAmB
Skin and subcutaneous tissue disorders	Posaconazole	Micafungin
Respiratory, thoracic, and mediastinal disorders	Isavuconazole	LAmB
Renal and urinary disorders	Itraconazole	LAmB
Hepatobiliary disorders	Caspofungin	Voriconazole
General disorders and administrative site conditions	Fluconazole	LAmB
Gastrointestinal disorders	Caspofungin	Itraconazole
Cardiac disorders	Caspofungin	Posaconazole

AEs, adverse events; LAmB, liposomal amphotericin B; SUCRA, Surface under the cumulative ranking curve.

### Adverse Events Analysis

We sorted out 23 AEs by system organ classes, of which nine were referred to by more than ten trials. We also sorted out six laboratory AEs, of which four were referred to by more than ten trials. We used these 13 AEs to conduct 13 network meta-analyses. Table 1 summarizes the antifungal agents involved in each AE and the number of studies included. Whether there is a significant difference in the odds ratio of incidence of AEs between the nine antifungal agents and placebo or between them in pairwise comparison is shown in the league tables. The ranking of antifungal agents in each AE is shown in the SUCRA Table 2.

#### Cardiac Disorders

In 17 studies, eight antifungal agents were reported to be involved in this disorder, network plot see Supplementary Figure S3. There was no significant difference in the incidence of cardiac disorders between any of antifungal agent and placebo. Caspofungin was more significantly associated with a lower incidence of cardiac disorders than LAmB was (OR = 0.18 (0.00,11.87)). Supplementary Table S2.35. Among the SUCRA rankings, caspofungin was the best and posaconazole was the worst Table 2.

#### Gastrointestinal Disorders

In 51 studies, nine antifungal agents were reported to be involved in this disorder, network plot see Supplementary Figure S4. Compared with placebo, caspofungin had a significantly lower incidence of gastrointestinal disorders (OR = 0.42 (0.18,0.95)), while itraconazole had a significantly higher one (OR = 2.03 (1.10,3.77)). Caspofungin and micafungin were more significantly associated with a lower incidence of gastrointestinal disorders than LAmB was (OR = 0.28 (0.15,0.53) and 0.52 (0.28,0.98), respectively). Supplementary Table S2.32. Among the SUCRA rankings, caspofungin was the best and itraconazole was the worst Table 2.

#### General Disorders

In 46 studies, nine antifungal agents were reported to be involved in this disorder, network plot see Supplementary Figure S5. Compared with placebo, LAmB had a significantly higher incidence of general disorders (OR = 5.10 (1.80,14.45)). Caspofungin, micafungin, fluconazole and itraconazole were more significantly associated with a lower incidence of general disorders than LAmB was (OR = 0.17 (0.06,0.46), 0.28 (0.09,0.86), 0.15 (0.06,0.36) and 0.28 (0.11,0.72), respectively). Supplementary Table S2.29. Among the SUCRA rankings, fluconazole was the best and LAmB was the worst Table 2.

#### Hepatobiliary Disorders

In 23 studies, eight antifungal agents were reported to be involved in this disorder, network plot see Supplementary Figure S6. Compared with placebo, caspofungin had a significantly lower incidence of hepatobiliary disorders (OR = 0.16 (0.05,0.54)). Caspofungin was more significantly associated with a lower incidence of hepatobiliary disorders than LAmB was (OR = 0.18 (0.08,0.43)). Supplementary Table S2.26. Among the SUCRA rankings, caspofungin was the best and voriconazole was the worst Table 2.

#### Renal and Urinary Disorders

In 16 studies, seven antifungal agents were reported to be involved in this disorder, network plot see Supplementary Figure S7. There was no significant difference in the incidence of renal and urinary disorders between any of antifungal agent and placebo. Caspofungin, fluconazole, itraconazole and voriconazole were more significantly associated with a lower incidence of renal and urinary disorders than LAmB was (OR = 0.32 (0.11,0.94), 0.20 (0.07,0.61), 0.12 (0.04,0.39), 0.17 (0.03,0.87), respectively). Supplementary Table S2.23. Among the SUCRA rankings, itraconazole was the best and LAmB was the worst Table 2.

#### Respiratory, Thoracic, and Mediastinal Disorders

In 15 studies, seven antifungal agents were reported to be involved in this disorder, network plot see Supplementary Figure S8. Compared with placebo, LAmB had a significantly higher incidence of respiratory, thoracic, and mediastinal disorders (OR = 3.88 (1.11,13.60)). Isavuconazole, micafungin and voriconazole were more significantly associated with a lower incidence of respiratory, thoracic, and mediastinal disorders than LAmB was (OR = 0.08 (0.03,0.24), 0.16 (0.04,0.63), 0.08 (0.03,0.24), respectively). Supplementary Table S2.21. Among the SUCRA rankings, isavuconazole was the best and LAmB was the worst Table 2.

#### Skin and Subcutaneous Tissue Disorders

In 42 studies, eight antifungal agents were reported to be involved in this disorder, network plot see Supplementary Figure S9. Compared with placebo, voriconazole had a significantly higher incidence of skin and subcutaneous tissue disorders (OR = 2.93 (1.12,7.67)). Compared with LAmB, all of seven antifungal agents had no significant difference in incidence of skin and subcutaneous tissue disorders. Supplementary Table S2.18. Among the SUCRA rankings, posaconazole was the best and micafungin was the worst Table 2.

#### Vascular Disorders

In 24 studies, six antifungal agents were reported to be involved in this disorder, network plot see Supplementary Figure S10. There was no significant difference in incidence of vascular disorders between any antifungal agent and placebo. Fluconazole was more significantly associated with a lower incidence of vascular disorders than LAmB was (OR = 0.26 (0.10,0.68)). Supplementary Table S2.17. Among the SUCRA rankings, placebo was the best and LAmB was the worst Table 2.

#### Nervous System Disorders

In 42 studies, nine antifungal agents were reported to be involved in this disorder, network plot see Supplementary Figure S11. Compared with placebo, isavuconazole and voriconazole had a significantly higher incidence of nervous system disorders (OR = 6.03 (1.09,33.55) and 8.66 (3.23,23.21), respectively). Isavuconazole and voriconazole were more significantly associated with a higher incidence of nervous system disorders than LAmB was (OR = 8.46 (1.53,46.80) and 12.15 (4.58,32.25), respectively). Supplementary Table S2.14. Among the SUCRA rankings, posaconazole was the best and isavuconazole was the worst Table 2.

#### Increase in Liver Enzymes

In 29 studies, eight antifungal agents were reported to be involved in this disorder, network plot see Supplementary Figure S12. There was no significant difference in incidence of increase in liver enzymes between any antifungal agent and placebo. Anidulafungin and caspofungin were more significantly associated with a lower incidence of increase in liver enzymes than LAmB was (OR = 0.18 (0.03,0.98) and 0.58 (0.39,0.86), respectively). Supplementary Table S2.11. Among the SUCRA rankings, anidulafungin was the best and posaconazole was the worst Table 2.

#### Decrease in Potassium

In 23 studies, six antifungal agents were reported to be involved in this disorder, network plot see Supplementary Figure S13. Compared with placebo, fluconazole and voriconazole had a significantly lower incidence of a decrease in potassium (OR = 0.03 (0.00,0.61) and 0.03 (0.00,0.65), respectively). Caspofungin, micafungin, fluconazole, itraconazole and voriconazole were more significantly associated with a lower incidence of a decrease in potassium than LAmB was (OR = 0.33 (0.16,0.71), 0.35 (0.12,0.98), 0.18 (0.08,0.41), 0.40 (0.19,0.85), and 0.17 (0.05,0.60), respectively) Supplementary Table S2.8. Among the SUCRA rankings, fluconazole was the best and placebo was the worst Table 2.

#### Increase in Total or Direct Bilirubin

In 14 studies, seven antifungal agents were reported to be involved in this disorder, network plot see Supplementary Figure S14. There was no comparison result between them and placebo, and the eight antifungal agents had no significant difference in incidence of increase in total or direct bilirubin Supplementary Table S2.6. Among the SUCRA rankings, caspofungin was the best and micafungin was the worst Table 2.

#### Increase in Creatinine

In 11 studies, six antifungal agents were reported to be involved in this disorder, network plot see Supplementary Figure S15. There was no significant difference in incidence of an increase in creatinine between any antifungal agent and placebo. Caspofungin was more significantly associated with a lower incidence of an increase in creatinine than LAmB was (OR = 0.11 (0.05,0.25)) Supplementary Table S2.4. Among the SUCRA rankings, caspofungin was the best and fluconazole was the worst Table 2.

### Subgroup Analysis

The results of the comparative analysis of incidence of above 13 AEs associated with antifungal agents in the “prophylaxis subgroup” and the “therapy subgroup” (including empirical therapy for patients with neutropenia and therapy for patients with fungal infections) are shown in Supplementary Table S2. The best and the worst antifungal agents are shown in Supplementary Table S3 (prophylaxis therapy subgroup), Supplementary Table S4 (empirical/definitive therapy subgroup) according to the SUCRA for incidence of AEs.

Five antifungal agents were reported in tolerability of prophylaxis therapy subgroup. Compared with placebo, LAmB and itraconazole had a significantly high incidence of discontinuation of therapy due to AEs (OR = 5.51 (1.91,15.93) and 2.79 (1.09,7.13), respectively). Compared with LAmB, micafungin, fluconazole, Itraconazole, and posaconazole had a significantly low incidence of discontinuation of therapy due to AEs (OR = 0.18 (0.06,0.52), 0.27 (0.12,0.64), 0.51 (0.30,0.87) and 0.25 (0.09,0.65), respectively) Supplementary Table S2.3. In SUCRA ranking, Placebo was the best and LAmB was the worst Supplementary Table S2. The results of tolerability of the empirical/definitive therapy subgroup were consistent with the result of ignoring subgroups Supplementary Table S2.2.

### Inconsistency and Publication Bias Assessment

The assessment of design-by-treatment model did not detect any significant global inconsistency. A *p* value <0.05 indicates a significant inconsistency (Supplementary Tables S5–S7). Only three subgroups analysis showed inconsistent fitting (*p* value ≤0.05). We perform network meta-analysis under inconsistency model instead of consistency model. In addition, 14 comparison-adjusted funnel plots of tolerability and 13 AEs were all roughly symmetrical, revealing no publication bias across studies (Supplementary Figures S16–S29).

## Discussion

The incidence of invasive fungal diseases (IFDs) has somewhat increased over the past decades (Falci and Pasqualotto, 2013), and long duration and high cost of IFD treatment highlight the importance of drug tolerability and safety. In this study, a network meta-analysis of AEs associated with nine commonly used antifungal agents was conducted. A total of 66 RCT studies consisting of 18,230 samples were included. This work includes three perspectives: the antifungal agent tolerability, clinical AEs and laboratory AEs. Meanwhile, a subgroup analysis of AEs associated with prophylactic and therapeutic drugs was conducted.

### Tolerability Analysis

In this study, tolerability was measured by odds ratio of the number of withdrawals from study medication due to AEs, excluding withdrawals for other reasons, such as loss of contact, patient’s willingness. Our study found that LAmB has the highest risk of withdrawals, with a withdrawal rate as high as 3.20 times that of placebo. Notably, both voriconazole and itraconazole have similarly poor tolerability performance, with withdrawal rates 2.50 and 2.39 times that of placebo, respectively. As a second-generation triazole, voriconazole is significantly less tolerated than posaconazole, and the withdrawal rate is 2.06 times that of the first-generation triazole fluconazole. Posaconazole is the best tolerated of all azoles. This suggests that although voriconazole has a good therapeutic efficacy, patient tolerability is a problem that requires special attention, especially in patients with severe debilitating conditions. Posaconazole is well tolerated but can only be taken orally, which limits its use in patients who cannot eat normally. Our study also found that echinocandins have a significantly stronger tolerability than azoles and polyenes, with caspofungin performing best, and anidulafungin and micafungin ranking higher than placebo. It can be seen that echinocandins are very well-tolerated drugs, but their use is restricted due to the problems of the strain selection, intravenous injection and high price. Nevertheless, we believe that the use of echinocandins is a necessary option for ICU patients with invasive *Candida* and aspergillosis infections.

### Echinocandins

Echinocandins are a relatively new class of antifungal agents. The three approved echinocandins (caspofungin, micafungin and anidulafungin) have similar chemical structures and exert antifungal activity to inhibit b-glucan synthesis by targeting 1,3-b-d-glucan synthase. B-glucan is a major component of fungal cell walls (Chen et al., 2011). There is no similar target structure in humans, which explains the good tolerability and safety of echinocandins compared with other classes of antifungal agents. Our study found that in terms of laboratory indicators, the risk of elevated liver enzymes and decreased potassium in echinocandins is lower than that in azoles and polyenes, but the risk of elevated total bilirubin is highest in micafungin. We found in the “therapy subgroup” that the bilirubin elevation rate of micafangin is 5.79 times that of itraconazole, which is a notable phenomenon. We also found that micafungin has the highest risk of skin and subcutaneous tissue disorders, and that caspofungin also has a higher risk of the same disorders than azoles. Caspofungin and micafungin are less likely than LAmB to cause renal and urinary disorders, but are at higher risk than other azoles. In addition, micafungin, also known as echinocandins, has a risk of hepatobiliary disorders as high as 4.16 times that of caspofungin. These AEs of echinocandins deserve attention in clinical treatment.

### Triazole

Trizole antifungal agents are used to treat IFD caused by a range of medically important opportunistic fungal pathogens (Livermore and Hope, 2012). Trizole antifungal inhibits the enzyme lanosterol demethylase by blocking the biosynthesis of ergosterol (Zhang et al., 2014), mainly including fluconazole, itraconazole, isavuconazole,posaconazole and voriconazole. As the most widely used antifungal agent, trazole has currently attracted increasing attention for its AEs. Our study found that the risk of elevated liver enzymes is higher with triazoles than with echinocandins, LAmB and placebo in laboratory indicators. The risk of posaconazole-induced elevation of liver enzymes is 10.75 times that of anidulafungin. Therefore, attention should be paid to the changes of liver function indicators in the treatment, especially in patients with liver dysfunction. Among the clinical AEs, voriconazole and isavuconazole have the highest incidence of nervous system disorders (mainly including visual disturbances, epilepsy, depression, insomnia, etc.), which is 8.66 times and 6.03 times that in placebo, respectively. However, posaconazole has a minimal risk of nervous system disorders and is superior to placebo and echinocandins. Voriconazole and isavuconazole also have a higher risk of general disorders (including pyrexia, weight loss, chills, asthenia, pain, etc.) than echinocandins and placebo. Itraconazole has a higher risk of gastrointestinal disorders than other drugs, and posaconazole has the highest risk of heart disease.

### LAmB

LAmB is a polyene antifungal agent. It is once regarded as the main method of antifungal treatment, but its efficacy is increasingly limited due to safety concerns. However, LAmB remains important in dealing with mucormycosis, cryptococcal and other emerging yeast infections, as well as in rescuing multiple mold and yeast infections (Falci and Pasqualotto, 2013). Our study found that LAmB is at a higher risk for decrease in potassium, skin and subcutaneous tissue disorders, respiratory, thoracic and mediastinal disorders, renal and urinary disorders, general disorders, gastrointestinal disorders, and cardiac disorders. Although LAmB is associated with many AEs, fully understanding and rational management of these AEs in clinical treatment will also contribute to the correct use of LAmB.

### Limitations

First, as with all pooled analyses, network meta-analysis should combine the results of similar studies only. However, factors contributing to non-statistical heterogeneity are difficult to quantify, and subjective assessment is essential to determine the RCT to be included (Kim et al., 2014). Despite repeated assessments by three our authors, heterogeneity is inevitable in this study. Our analysis of influencing factors showed that age, follow-up time, and drug use dose were responsible for the heterogeneity observed in the overall efficacy analysis. Therefore, our findings on the AEs of these drugs should be interpreted cautiously in conjunction with individual practice. Second, due to ethical issues, few studies used placebo as a control group, while most of the studies used LAmB as the control group. Therefore, we focused mainly on comparisons with LAmB and pairwise comparisons between drugs rather than with placebo. Third, because of the numerous and inconsistent descriptions of AE in each study, we must subjectively sort and combine the data, which is bound to differ from the original author’s understanding and will have a certain impact on the results. Fourth, this network meta-analysis included a number of small-studies, so it is possible to overestimate the effect size.

## Conclusion

This network meta-analysis found that echinocandins are the most tolerated antifungal agents with high safety, while the impacts of micafungin on liver function and skin deserve attention. In addition, the skin and subcutaneous tissue disorders in caspofungin also require attention. The AEs of triazole are mainly concentrated on the increase in liver enzymes, nervous system disorders, especially visual disorders, general disorders, gastrointestinal disorders, and cardiac diseases. Besides, the low tolerability and the risk of increase in liver enzymes and visual disorders associated with voriconazole, the risk of increase in liver enzymes and visual disorders associated with isavuconazole, the low tolerability and the risk of gastrointestinal disorders associated with itraconazole, as well as the risk of increase in liver enzymes and cardiac diseases associated with posaconazole should be given special attention. LAmB is the least tolerated and has the most abundant AEs, such as decrease in potassium, renal and urinary disorders and cardiac diseases, etc., which should be given full attention in clinical treatment.

## Data Availability

The original contributions presented in the study are included in the article/Supplementary Material, further inquiries can be directed to the corresponding author.

## Abbreviations

AEs, adverse events; IFDs, invasive fungal diseases; IFI, Invasive fungal infections; LAmB, liposomal amphotericin B; SUCRAs, surface under the cumulative ranking curves; OR, Odds Ratio; PICOS, Participant, Intervention, Comparison, Outcome, Study design; RCT, Randomized Controlled Trial.
