# Supplementary material for: Adverse Effects Associated With Currently Commonly Used Antifungal Agents: A Network Meta-Analysis and Systematic Review
Source: Front Pharmacol. 2021 Oct 29;12:697330. doi: 10.3389/fphar.2021.697330 (PMC8585744; doi:10.3389/fphar.2021.697330)
Supplement: Supplementary file 1 [file DataSheet1.docx]

Supplementary Material

1. **Supplementary Result S1:** The process of network meta-analysis

# Supplementary Figures and Tables

## 2.1 Supplementary Tables

**Table S1.** Characteristics of included studies

**Table S2.** League table showing the results of the network meta-analysis comparing the effects (Odds ratio OR) for each AE of all antifungal drugs and 95% confidence intervals (95% CI)

S2.1. Withdrawal from study medication due to adverse events

S2.2. Withdrawal from study medication due to adverse events - empirical /definitive therapy subgroup

S2.3. Withdrawal from study medication due to adverse events - prophylaxis therapy subgroup

S2.4. Increase in creatinine

S2.5. Increase in creatinine - empirical /definitive therapy subgroup

S2.6. Increase in total or direct bilirubin

S2.7. Increase in total or direct bilirubin - empirical /definitive therapy subgroup

S2.8. Decrease in potassium

S2.9. Decrease in potassium - empirical /definitive therapy subgroup

S2.10. Decrease in potassium - prophylaxis therapy subgroup

S2.11. Increase in liver enzymes

S2.12. Increase in liver enzymes - empirical /definitive therapy subgroup

S2.13. Increase in liver enzymes - prophylaxis therapy subgroup

S2.14. Nervous system disorders

S2.15. Nervous system disorders - empirical /definitive therapy subgroup

S2.16 Nervous system disorders - prophylaxis therapy subgroup

S2.17. Vascular disorders

S2.18. Skin and subcutaneous tissue disorders

S2.19. Skin and subcutaneous tissue disorders - empirical /definitive therapy subgroup

S2.20. Skin and subcutaneous tissue disorders - prophylaxis therapy subgroup

S2.21. Respiratory, thoracic, and mediastinal disorders

S2.22. Respiratory, thoracic, and mediastinal disorders - empirical /definitive therapy subgroup

S2.23. Renal and urinary disorders

S2.24. Renal and urinary disorders - empirical /definitive therapy subgroup

S2.25. Renal and urinary disorders - prophylaxis therapy subgroup

S2.26. Hepatobiliary disorders

S2.27. Hepatobiliary disorders - empirical /definitive therapy subgroup

S2.28. Hepatobiliary disorders - prophylaxis therapy subgroup

S2.29. General disorders and administrative site conditions

S2.30. General disorders and administrative site conditions - empirical /definitive therapy subgroup

S2.31. General disorders and administrative site conditions - prophylaxis therapy subgroup

S2.32. Gastrointestinal disorders

S2.33. Gastrointestinal disorders - empirical /definitive therapy subgroup

S2.34. Gastrointestinal disorders - prophylaxis therapy subgroup

S2.35. Cardiac disorders

S2.36. Cardiac disorders - empirical /definitive therapy subgroup

S2.37. Cardiac disorders - prophylaxis therapy subgroup

**Table S3.** According to SUCRA, the best (with the lowest side effect rate) and the worst (with the highest side effect rate) antifungal agents: prophylaxis therapy subgroup

**Table S4.** According to SUCRA, the best (with the lowest side effect rate) and the worst (with the highest side effect rate) antifungal agents: empirical /definitive therapy subgroup

**Table S5.** The inconsistency estimation results of each AE meta-analysis

**Table S6.** The inconsistency estimation results of each AE meta-analysis: empirical /definitive therapy subgroup

**Table S7.** The inconsistency estimation results of each AE meta-analysis: prophylaxis therapy subgroup

## 2.2 Supplementary Figures

**Figure S1** Risk of bias graph

**Figure S2** Risk of bias summary graph

**Figure S3** Network plot for Cardiac disorders analysis

**Figure S4** Network plot for Gastrointestinal disorders analysis

**Figure S5** Network plot for General disorders and administrative site conditions analysis

**Figure S6** Network plot for Hepatobiliary disorders analysis

**Figure S7** Network plot for Renal and urinary disorders analysis

**Figure S8** Network plot for Respiratory, thoracic, and mediastinal disorders analysis

**Figure S9** Network plot for Skin and subcutaneous tissue disorders analysis

**Figure S10** Network plot for Vascular disorders analysis

**Figure S11** Network plot for Nervous system disorders analysis

**Figure S12** Network plot for Increase in liver enzymes analysis

**Figure S13** Network plot for Decrease in potassium analysis

**Figure S14** Network plot for Increase in total or direct bilirubin analysis

**Figure S15** Network plot for Increase in creatinine analysis

**Figure S16** Funnel plot for Cardiac disorders analysis

**Figure S17** Funnel plot for Gastrointestinal disorders analysis

**Figure S18** Funnel plot for General disorders and administrative site conditions analysis

**Figure S19** Funnel plot for Hepatobiliary disorders analysis

**Figure S20** Funnel plot for Renal and urinary disorders analysis

**Figure S21** Funnel plot for Respiratory, thoracic, and mediastinal disorders analysis

**Figure S22** Funnel plot for Skin and subcutaneous tissue disorders analysis

**Figure S23** Funnel plot for Vascular disorders analysis

**Figure S24** Funnel plot for Nervous system disorders analysis

**Figure S25** Funnel plot for Increase in liver enzymes analysis

**Figure S26** Funnel plot for Decrease in potassium analysis

**Figure S27** Funnel plot for Increase in total or direct bilirubin analysis

**Figure S28** Funnel plot for Increase in creatinine analysis

**Figure S29** Funnel plot for tolerability analysis

**3 References**

**Supplementary Result S1:** The process of network meta-analysis

We sorted out 23 AEs by system organ classes, of which nine were referred to by more than ten trials. We also sorted out 6 laboratory AEs, of which four were referred to by more than ten trials. We used these 13 AEs to conduct 13 network meta- analyses. We drew the overall network plot (Figure 2) and 13 sub-network plots (Figure S3-Figure S15). The larger the nodes in the network plot, the more patients were included in the corresponding intervention, and the thicker the line, the more intervention studies were directly compared. Table 1 was a quantitative description of the 13 network plots, illustrating the specific number of interventions in each subnet and corresponding included studies. Before performing the network meta-analysis, we conducted a consistency test (Tables S5-S7), and selected the corresponding network meta-analysis model based on the results. The results of the pairwise comparison of the incidence of each AE of the 9 antifungal agents were shown in the league tables. The subgroup analyses of "prophylaxis" and "therapy (including empirical therapy for patients with neutropenia and therapy for patients with fungal infections)" were also carried out, (Table S2.1-Table S2.37). We also SUCRA ranked 9 antifungal agents among 13 AEs. Finally, a visualization of public bias was performed using comparison-adjusted funnel plots (Figure S16-S29).

**Table S1.** Characteristics of included studies

| Study | Year | Study design | Age | Male% | Type | Antifungal agents | Fungal | Patients | Sample size | Country |
| --- | --- | --- | --- | --- | --- | --- | --- | --- | --- | --- |
| Aaron (Aaron et al., 2012) | 2012 | RCT double-blind | 25.3 (10.5) | 56.0% | definitive | itraconazole | Aspergillus fumigatus | Cystic Fibrosis | 35 | Canada |
| Ally (Ally et al., 2001) | 2001 | RCT double-blind | 18 to 75 |  | definitive | Voriconazole, Fluconazole | Esophageal Candidiasis | esophageal candidiasis in immunocompromised patients | 391 | France |
| Arathoon (Arathoon et al., 2002) | 2002 | RCT double-blind | 18 to 65 | 79.0% | definitive | Caspofungin, LAmB | Candidiases | Oropharyngeal and Esophageal Candidiases | 140 | Guatemala |
| Boogaerts (Boogaerts et al., 2001a) | 2001 | RCT open-label | 45 | 61.1% | prophylaxis | Itraconazole, amphotericin B | - | neutropenic cancer patients | 277 | Belgium |
| Boogaerts (Boogaerts et al., 2001b) | 2001 | RCT open-label | 18 to 81 | 50.0% | prophylaxis | Itraconazole, Amphotericin B | - | neutropenic cancer patients | 384 | Belgium |
| Chariyalertsak (Chariyalertsak et al., 2002) | 2002 | RCT double-blind | 33.4 (22 to 51) | 38.1% | prophylaxis | Itraconazole | - | Immunodeficiency Virus Infection | 129 | Thailand |
| Cornely (Cornely et al., 2007) | 2007 | RCT double-blind | 50(16) | 54.0% | prophylaxis | Posaconazole, Fluconazole, Itraconazole | - | Neutropenia | 602 | Germany |
| Cornely (Cornely et al., 2015) | 2015 | RCT open-label | 53.6 | 64.7% | definitive | micafungin | aspergillosis | invasive aspergillosis | 17 | Germany |
| Corvò (Corvò et al., 2008) | 2008 | RCT double-blind | 31 (22.5) | 77.5% | prophylaxis | fluconazole | oropharyngeal candidiasis | patients undergoing radiotherapy for head and neck tumour | 268 | Italy |
| de Wet (de Wet et al., 2004) | 2004 | RCT double-blind | 35.5(8.1) | 46.7% | definitive | Micafungin, Fluconazole | Esophageal Candidiasis | HIV-Positive Patients | 245 | South Africa |
| de Wet (de Wet et al., 2005) | 2005 | RCT double-blind | 37.2(10.59) | 50.4% | definitive | micafungin, fluconazole | oesophageal candidiasis | patients with documented clinical symptoms of Oesophageal candidiasis | 518 | South Africa |
| Dowell (Dowell et al., 2005) | 2005 | RCT double-blind | 20 to 40 | 100.0% | definitive | Anidulafungin, Voriconazole | - | healthy | 17 | USA |
| Galgiani (Galgiani et al., 2000) | 2000 | RCT double-blind | 37 | 74.0% | definitive | Fluconazole, Itraconazole | Coccidioidomycosis | patients with chronic pulmonary, soft tissue, or skeletal coccidioidal infections | 191 | USA |
| Gallin (Gallin et al., 2003) | 2003 | RCT double-blind | 5.0 to 56.7 | 85.0% | prophylaxis | Itraconazole | - | Patients with chronic granulomatous disease | 39 | USA |
| Glasmacher (Glasmacher et al., 2006) | 2006 | RCT open-label | - | 56.5% | prophylaxis | itraconazole, fluconazole | - | patients with haematological malignancy and profound neutropenia | 494 | Germany |
| Groll (Groll et al., 2010) | 2010 | RCT open-label | 40.4(12.6) | 61.1% | empirical | Caspofungin, LAmB | - | granulocytopenia | 38 | Germany |
| Harousseau (Harousseau et al., 2000) | 2000 | RCT open-label | 48 (15 to 75) | 59.4% | empirical | Itraconazole, Amphotericin B | - | Patients with Hematological Malignancy and Profound Neutropenia | 557 | France |
| Herbrecht (Herbrecht et al., 2002) | 2002 | RCT open-label | 48.5(13 to 79) | 68.1% | empirical | VORICONAZOLE, LAmB | ASPERGILLOSIS | with definite or probable invasive aspergillosis | 277 | France |
| Hiramatsu (Hiramatsu et al., 2008) | 2008 | RCT open-label | 46.5 | 64.0% | empirical | micafungin, fluconazole | - | neutropenic patients receiving hematopoietic stem cell transplantation | 100 | Japan |
| Huang (Huang et al., 2012) | 2012 | RCT open-label | 32.1 (10.1) | 64.0% | empirical | Micafungin,  Itraconazole | - | Patients undergoing Hematopoietic Stem Cell Transplant | 283 | China |
| Ito (Ito et al., 2007) | 2007 | RCT open-label | 58 (16 to 80) | 55.3% | empirical | Itraconazole, Fluconazole | - | Patients with Acute Myeloid Leukemia and Myelodysplastic Syndromes | 209 | Japan |
| Jarvis (Jarvis et al., 2019) | 2019 | RCT open-label | 38 (32 to 43) | 54.0% | empirical | LAmB | Cryptococcal | Human immunodeficiency virus (HIV)-infected adults with Cryptococcal Meningitis | 79 | UK |
| Jeong (Jeong et al., 2016) | 2016 | RCT open-label | 49 (20 to 85) | 57.5% | empirical | micafungin, itraconazole | - | febrile neutropenic patients with hematological malignancies | 148 | South Korea |
| Kakuda (Kakuda et al., 2013) | 2013 | RCT open-label | 29 (18 to 45) | 83.3% | empirical | Fluconazole, Voriconazole | - | HIV-Negative Volunteers | 18 | USA |
| Keirns (Keirns et al., 2017) | 2017 | RCT double-blind | 33.0(10.6) | 41.5% | empirical | Isavuconazole |  | healthy individuals | 160 | USA |
| Kohno (Kohno et al., 2010) | 2010 | RCT open-label | 70.9 | 77.6% | empirical | micafungin, voriconazole | aspergillosis | chronic pulmonary aspergillosis | 107 | Japan |
| Kohno (Kohno et al., 2013) | 2013 | RCT double-blind | 69.1(10.1) | 79.3% | empirical | caspofungin, micafungin | candidiasis, aspergillosis | patients with fungal infections | 121 | Japan |
| Krause (Krause et al., 2004) | 2004 | RCT double-blind | 37.5(10.4) | 42.3% | empirical | Anidulafungin, Fluconazole | Candidiasis | had esophageal candidiasis diagnosed and who had a predisposing risk | 601 | USA |
| Krishna (Krishna et al., 2012) | 2012 | RCT double-blind | 47.7 (8.2) | 50.0% | empirical | posaconazole | - | healthy volunteers | 25 | USA |
| Kuse (Kuse et al., 2007) | 2007 | RCT double-blind | 54·5 (18 to 89) | 63.0% | empirical | Micafungin, LAmB | candidaemia | had clinical signs of systemic candida infection | 531 | Germany |
| Le (Le et al., 2017) | 2017 | RCT open-label | 34 (30 to 38) | 70.7% | empirical | Itraconazole, LAmB | Talaromycosis | HIV-infected adults who had talaromycosis | 435 | UK |
| Lefebvre (Lefebvre and Domenge, 2002) | 2002 | RCT open-label | 59(11) | 83.0% | empirical | fluconazole, LAmB | candidiasis | cancer who were suffering from candidiasis during cancer treatment | 268 | France |
| Liu (Liu et al., 2007) | 2007 | RCT open-label | 18 to 55 | 100.0% | empirical | Voriconazole | - | healthy nonsmoking males | 34 | USA |
| Maertens (Maertens et al., 2010) | 2010 | RCT double-blind | 2 to 17 | - | empirical | Caspofungin, LAmB | - | Pediatric Patients with Persistent Fever and Neutropenia | 81 | Belgium |
| Maertens (Maertens et al., 2016) | 2016 | RCT double-blind | 51·1 (16·2) | 56.0% | empirical | Isavuconazole, voriconazole | Aspergillus | Patients with suspected invasive mould disease | 516 | Germany |
| Malik (Malik et al., 1998) | 1998 | RCT open-label | 33(17) | 69.0% | empirical | Fluconazole, LAmB | - | Cancer Patients with Prolonged Fever and Neutropenia | 100 | Pakistan |
| Mandhaniya (Mandhaniya et al., 2011) | 2011 | RCT open-label | 5.5 (1.5 to 15) | 74.0% | empirical | Voriconazole Amphotericin B |  | children with denovo acute myeloid leukemia and acute lymphoblastic leukemia | 100 | UK |
| Marks (Marks et al., 2011) | 2011 | RCT open-label |  |  | empirical | Voriconazole, itraconazole |  | allogeneic haematopoietic stem-cell transplant recipients | 465 | UK |
| Mattiuzzi (Mattiuzzi et al., 2011) | 2011 | RCT open-label | 59(23 to 83) | 63.0% | empirical | voriconazole, itraconazole |  | patients with acute myelogenous leukemia or high-risk myelodysplastic syndrome | 123 | USA |
| McKinsey (McKinsey et al., 1999) | 1999 | RCT double-blind | 37 | 96.0% | empirical | Prophylaxis | - | Patients with Advanced Human Immunodeficiency Virus Infection | 295 | USA |
| Migoya (Migoya et al., 2011) | 2011 | RCT double-blind | 18 to 45 | - | empirical | Caspofungin | - | Healthy Adult Participants | 20 | USA |
| Mora-Duarte (Mora-Duarte et al., 2002) | 2002 | RCT double-blind | 56(17 to 84) | 51.4% | empirical | CASPOFUNGIN, LAmB | CANDIDIASIS | patients who had clinical evidence of infection and a positive culture for candida. | 224 | Costa Rica |
| Ostrosky-Zeichner (Ostrosky-Zeichner et al., 2014) | 2014 | RCT double-blind | 58.2 (17.6) | 60.7% | empirical | Caspofungin | - | High-Risk Adults in the Critical Care Setting | 219 | USA |
| Oyake (Oyake et al., 2016) | 2016 | RCT open-label | 53 (18 to 80) | 54.0% | empirical | micafungin, voriconazole | - | febrile neutropenic patients with hematological disorders | 100 | Japan |
| Park (Park et al., 2006) | 2006 | matched case control | 35.8±12.5 | 64.6% | empirical | Itraconazole, LAmB |  | Patients with Persistent Neutropenic Fever | 96 | Korea |
| Parkes-Ratanshi (Parkes-Ratanshi et al., 2011) | 2011 | RCT double-blind | 35.9 (9.1) | 37.6% | empirical | fluconazole | cryptococcal | HIV positive Ugandan adults | 1519 | Uganda |
| Purkins (Purkins et al., 2003a) | 2003 | RCT single-blind | 27 (20 to 45) | 100.0% | empirical | voriconazole | - | healthy men | 56 | UK |
| Purkins (Purkins et al., 2003b) | 2003 warfarin | RCT double-blind | 24 (19 to 37) | 100.0% | empirical | Voriconazole | - | healthy male subjects | 14 | UK |
| Queiroz-Telles (Queiroz-Telles et al., 2007) | 2007 | RCT open-label | 48.3 (30 to 86) | 97.1% | definitive | Voriconazole, Itraconazole | Paracoccidioidomycosis | patientswith evidence of yeast cells consistent with P. brasiliensis, or culture positive for P. brasiliensis. | 53 | Brazil |
| Reboli (Reboli et al., 2007) | 2007 | RCT double-blind | 57.0 to 17.0 | 51.0% | definitive | Anidulafungin, Fluconazole | Candidiasis | Adults with invasive candidiasis | 245 | USA |
| Rotstein (Rotstein et al., 1999) | 1999 | RCT double-blind | 47.6 (18 to 80) | 53.2% | prophylaxis | Fluconazole | - | Neutropenic Cancer Patients | 274 | Canada |
| Saliba (Saliba et al., 2015) | 2015 | RCT open-label | 51.2 (11.2) | 67.4% | prophylaxis | Micafungin |  | High-Risk Liver Transplant Recipients | 344 | France |
| Schuster (Schuster et al., 2008) | 2008 | RCT double-blind | 53 (19) | 76.0% | Empirical | Fluconazole | - | ICU patients | 249 | USA |
| Shang (Shang et al., 2012) | 2012 | RCT open-label | 21 to 65 | 74.2% | definitive | micafungin, voriconazole | - | kidney transplant recipients | 65 | China |
| Sobel (Sobel et al., 2004) | 2004 | RCT open-label | 33.8( 18 to 65). | 0.0% | definitive | Fluconazole | Vulvovaginal Candidiasis | women with recurrent vulvovaginal candidiasis | 343 | USA |
| Taillandier (Taillandier et al., 2000) | 2000 | RCT open-label | 84(8) | 76.0% | definitive | fluconazole, LAmB | oropharyngeal candidosis | older patients with oropharyngeal candidosis | 305 | France |
| Ullmann (Ullmann et al., 2007) | 2007 | RCT double-blind | 42.2(13 to 72) | 67.0% | prophylaxis | posaconazole, fluconazole |  | patients with graft-versus-host disease who were receiving immunosuppressive therapy | 600 | Germany |
| van Burik (van Burik et al., 2004) | 2004 | RCT double-blind | 43.2 (0.6 to 73.0) | 59.5% | prophylaxis | Micafungin, Fluconazole | - | Patients Undergoing Hematopoietic Stem Cell Transplantation | 882 | USA |
| Vazquez (Vazquez et al., 2006) | 2006 | RCT double-blind | 36.4(7.8) | 74.0% | definitive | Posaconazole Fluconazole | Oropharyngeal Candidiasis | Subjects with HIV/AIDS | 350 | USA |
| Villanueva (Villanueva et al., 2001) | 2001 | RCT double-blind | (21 to 66) | 78.9% | definitive | Caspofungin, LAmB | Candidiasis | adults with endoscopically documented symptomatic Candida esophagitis | 128 | USA |
| Villanueva (Villanueva et al., 2002) | 2002 | RCT double-blind | 37(11) | 64.0% | definitive | Caspofungin, Fluconazole | Esophageal Candidiasis | patients had endoscopically and microbiologically documented Candida esophagitis | 177 | USA |
| Viscoli (Viscoli et al., 1996) | 1996 | RCT open-label | 27(1-73) | 68.0% | Empirical | Fluconazole, LAmB |  | Unexplained Fever in Granulocytopenic Cancer Patients | 112 | Italy |
| Walsh (Walsh et al., 2002) | 2002 | RCT open-label | 46.3(12 to 82) | 56.1% | EMPIRICAL | VORICONAZOLE, LAmB |  | IN PATIENTS WITH NEUTROPENIA AND PERSISTENT FEVER | 837 | USA |
| Walsh (Walsh et al., 2004) | 2004 | RCT double-blind | 51(17 to 83) | 57.2% | EMPIRICAL | Caspofungin,LAmB |  | in Patients with Persistent Fever and Neutropenia | 1095 | USA |
| Winston (Winston et al., 2000) | 2000 | RCT open-label | 47 (15) | 50.0% | prophylaxis | Fluconazole, Amphotericin B |  | Febrile Neutropenic Patients with Cancer | 317 | USA |
| Winston (Winston and Busuttil, 2002) | 2002 | RCT open-label | 53 (24 to 72) | 53.0% | prophylaxis | ITRACONAZOLE, FLUCONAZOLE |  | IN LIVER TRANSPLANT RECIPIENTS | 188 | USA |
| Total 66 | studies |  |  |  |  |  |  |  | 18230 |  |

**Table S2.** League table showing the results of the network meta-analysis comparing the effects (Odds ratio OR) for each AE of all antifungal drugs and 95% confidence intervals (95% CI)

**S2.1.** Withdrawal from study medication due to adverse events

| _Placebo_ | _LAmB_ | _Anidulafungin_ | _Caspofungin_ |  | _Micafungin_ | _Fluconazole_ | _Isavuconazole_ | _Itraconazole_ | _Posaconazole_ | _Voriconazole_ |
| --- | --- | --- | --- | --- | --- | --- | --- | --- | --- | --- |
| Placebo | 3.20 (1.81,5.66) | 0.76 (0.28,2.09) | 0.76 (0.35,1.64) |  | 1.02 (0.60,1.72) | 1.21 (0.76,1.94) | 1.69 (0.58,4.94) | 2.39 (1.41,4.05) | 1.11 (0.56,2.19) | 2.50 (1.30,4.81) |
| 0.31 (0.18,0.55) | LAmB | 0.24 (0.09,0.65) | 0.24 (0.13,0.43) |  | 0.32 (0.19,0.52) | 0.38 (0.23,0.62) | 0.53 (0.20,1.41) | 0.75 (0.52,1.06) | 0.35 (0.17,0.69) | 0.78 (0.47,1.30) |
| 1.32 (0.48,3.63) | 4.22 (1.54,11.57) | Anidulafungin | 1.00 (0.32,3.15) |  | 1.34 (0.49,3.66) | 1.60 (0.65,3.92) | 2.22 (0.59,8.45) | 3.15 (1.17,8.48) | 1.46 (0.52,4.07) | 3.29 (1.15,9.42) |
| 1.31 (0.61,2.82) | 4.21 (2.30,7.69) | 1.00 (0.32,3.13) | Caspofungin |  | 1.33 (0.66,2.72) | 1.59 (0.77,3.28) | 2.22 (0.71,6.89) | 3.14 (1.60,6.14) | 1.45 (0.61,3.49) | 3.28 (1.53,7.05) |
| 0.98 (0.58,1.67) | 3.15 (1.93,5.14) | 0.75 (0.27,2.04) | 0.75 (0.37,1.53) |  | Micafungin | 1.19 (0.75,1.91) | 1.66 (0.60,4.60) | 2.35 (1.47,3.76) | 1.09 (0.55,2.16) | 2.46 (1.39,4.36) |
| 0.82 (0.51,1.32) | 2.64 (1.62,4.29) | 0.63 (0.26,1.53) | 0.63 (0.30,1.29) |  | 0.84 (0.52,1.34) | Fluconazole | 1.39 (0.49,3.93) | 1.97 (1.26,3.07) | 0.91 (0.55,1.51) | 2.06 (1.14,3.74) |
| 0.59 (0.20,1.74) | 1.90 (0.71,5.06) | 0.45 (0.12,1.71) | 0.45 (0.15,1.40) |  | 0.60 (0.22,1.67) | 0.72 (0.25,2.03) | Isavuconazole | 1.42 (0.54,3.72) | 0.66 (0.21,2.06) | 1.48 (0.66,3.34) |
| 0.42 (0.25,0.71) | 1.34 (0.94,1.91) | 0.32 (0.12,0.85) | 0.32 (0.16,0.62) |  | 0.42 (0.27,0.68) | 0.51 (0.33,0.79) | 0.71 (0.27,1.85) | Itraconazole | 0.46 (0.24,0.90) | 1.05 (0.65,1.69) |
| 0.90 (0.46,1.78) | 2.89 (1.45,5.78) | 0.69 (0.25,1.91) | 0.69 (0.29,1.65) |  | 0.92 (0.46,1.82) | 1.10 (0.66,1.81) | 1.52 (0.48,4.79) | 2.16 (1.11,4.20) | Posaconazole | 2.26 (1.04,4.87) |
| 0.40 (0.21,0.77) | 1.28 (0.77,2.14) | 0.30 (0.11,0.87) | 0.30 (0.14,0.65) |  | 0.41 (0.23,0.72) | 0.49 (0.27,0.88) | 0.68 (0.30,1.52) | 0.96 (0.59,1.54) | 0.44 (0.21,0.96) | Voriconazole |

**S2.2.** Withdrawal from study medication due to adverse events - empirical /definitive therapy subgroup

| _Placebo_ | _LAmB_ | _Anidulafungin_ | _Caspofungin_ | _Micafungin_ | _Fluconazole_ | _Isavuconazole_ | _Itraconazole_ | _Posaconazole_ | _Voriconazole_ |
| --- | --- | --- | --- | --- | --- | --- | --- | --- | --- |
| Placebo | 2.81 (1.33,5.93) | 0.71 (0.22,2.25) | 0.69 (0.28,1.69) | 1.05 (0.48,2.30) | 1.10 (0.59,2.07) | 1.79 (0.51,6.23) | 2.61 (1.27,5.33) | 1.08 (0.32,3.69) | 2.57 (1.15,5.71) |
| 0.36 (0.17,0.75) | LAmB | 0.25 (0.08,0.80) | 0.25 (0.13,0.46) | 0.37 (0.21,0.67) | 0.39 (0.20,0.76) | 0.64 (0.21,1.90) | 0.93 (0.57,1.51) | 0.39 (0.11,1.36) | 0.91 (0.52,1.61) |
| 1.42 (0.44,4.52) | 3.98 (1.25,12.68) | Anidulafungin | 0.98 (0.28,3.49) | 1.48 (0.45,4.92) | 1.56 (0.60,4.06) | 2.53 (0.59,10.82) | 3.69 (1.13,12.04) | 1.54 (0.36,6.46) | 3.63 (1.12,11.77) |
| 1.44 (0.59,3.52) | 4.06 (2.18,7.53) | 1.02 (0.29,3.63) | Caspofungin | 1.51 (0.70,3.28) | 1.59 (0.69,3.68) | 2.58 (0.75,8.83) | 3.77 (1.78,7.95) | 1.57 (0.40,6.10) | 3.71 (1.67,8.24) |
| 0.96 (0.43,2.10) | 2.68 (1.50,4.81) | 0.67 (0.20,2.24) | 0.66 (0.31,1.43) | Micafungin | 1.05 (0.52,2.13) | 1.71 (0.54,5.39) | 2.49 (1.39,4.47) | 1.04 (0.29,3.75) | 2.45 (1.29,4.66) |
| 0.91 (0.48,1.70) | 2.55 (1.32,4.92) | 0.64 (0.25,1.67) | 0.63 (0.27,1.45) | 0.95 (0.47,1.93) | Fluconazole | 1.62 (0.50,5.30) | 2.37 (1.21,4.61) | 0.98 (0.33,2.92) | 2.33 (1.13,4.80) |
| 0.56 (0.16,1.95) | 1.57 (0.53,4.69) | 0.40 (0.09,1.69) | 0.39 (0.11,1.33) | 0.59 (0.19,1.85) | 0.62 (0.19,2.02) | Isavuconazole | 1.46 (0.48,4.42) | 0.61 (0.13,2.95) | 1.44 (0.59,3.53) |
| 0.38 (0.19,0.78) | 1.08 (0.66,1.75) | 0.27 (0.08,0.88) | 0.27 (0.13,0.56) | 0.40 (0.22,0.72) | 0.42 (0.22,0.82) | 0.68 (0.23,2.07) | Itraconazole | 0.42 (0.12,1.47) | 0.98 (0.57,1.70) |
| 0.92 (0.27,3.13) | 2.59 (0.74,9.11) | 0.65 (0.15,2.74) | 0.64 (0.16,2.48) | 0.97 (0.27,3.50) | 1.02 (0.34,3.01) | 1.65 (0.34,7.99) | 2.40 (0.68,8.51) | Posaconazole | 2.37 (0.65,8.57) |
| 0.39 (0.18,0.87) | 1.09 (0.62,1.93) | 0.28 (0.08,0.89) | 0.27 (0.12,0.60) | 0.41 (0.21,0.77) | 0.43 (0.21,0.89) | 0.70 (0.28,1.71) | 1.02 (0.59,1.76) | 0.42 (0.12,1.53) | Voriconazole |

**S2.3.** Withdrawal from study medication due to adverse events - prophylaxis therapy subgroup

| _Placebo_ |  | _LAmB_ | _Micafungin_ | _Fluconazole_ | _Itraconazole_ | _Posaconazole_ |
| --- | --- | --- | --- | --- | --- | --- |
| Placebo |  | 5.51 (1.91,15.93) | 1.01 (0.50,2.05) | 1.51 (0.69,3.33) | 2.79 (1.09,7.13) | 1.35 (0.53,3.45) |
| 0.18 (0.06,0.52) |  | LAmB | 0.18 (0.06,0.52) | 0.27 (0.12,0.64) | 0.51 (0.30,0.87) | 0.25 (0.09,0.65) |
| 0.99 (0.49,2.00) |  | 5.44 (1.91,15.49) | Micafungin | 1.49 (0.74,3.01) | 2.76 (1.10,6.95) | 1.34 (0.56,3.17) |
| 0.66 (0.30,1.46) |  | 3.65 (1.57,8.46) | 0.67 (0.33,1.35) | Fluconazole | 1.85 (0.93,3.67) | 0.90 (0.54,1.49) |
| 0.36 (0.14,0.91) |  | 1.97 (1.15,3.38) | 0.36 (0.14,0.91) | 0.54 (0.27,1.07) | Itraconazole | 0.48 (0.21,1.13) |
| 0.74 (0.29,1.89) |  | 4.08 (1.54,10.79) | 0.75 (0.32,1.78) | 1.12 (0.67,1.86) | 2.07 (0.89,4.80) | Posaconazole |

**S2.4.** Increase in creatinine

| _Placebo_ | _LAmB_ | _Caspofungin_ | _Micafungin_ | _Fluconazole_ | _Itraconazole_ | _Voriconazole_ |
| --- | --- | --- | --- | --- | --- | --- |
| Placebo | 3.20 (0.36,28.17) | 0.36 (0.04,3.68) | 1.46 (0.14,15.03) | 7.52 (0.14,407.14) | 0.98 (0.07,13.56) | 1.69 (0.17,16.52) |
| 0.31 (0.04,2.75) | LAmB | 0.11 (0.05,0.25) | 0.45 (0.19,1.06) | 2.35 (0.08,66.77) | 0.30 (0.07,1.34) | 0.53 (0.27,1.05) |
| 2.77 (0.27,28.21) | 8.86 (3.93,19.95) | Caspofungin | 4.03 (1.25,12.95) | 20.81 (0.67,649.81) | 2.70 (0.50,14.55) | 4.68 (1.68,13.03) |
| 0.69 (0.07,7.09) | 2.20 (0.94,5.14) | 0.25 (0.08,0.80) | Micafungin | 5.17 (0.17,161.63) | 0.67 (0.12,3.63) | 1.16 (0.45,2.99) |
| 0.13 (0.00,7.20) | 0.43 (0.01,12.09) | 0.05 (0.00,1.50) | 0.19 (0.01,6.05) | Fluconazole | 0.13 (0.01,2.61) | 0.22 (0.01,6.62) |
| 1.03 (0.07,14.26) | 3.28 (0.75,14.45) | 0.37 (0.07,2.00) | 1.49 (0.28,8.08) | 7.71 (0.38,154.99) | Itraconazole | 1.73 (0.36,8.28) |
| 0.59 (0.06,5.79) | 1.89 (0.96,3.75) | 0.21 (0.08,0.60) | 0.86 (0.34,2.22) | 4.45 (0.15,131.26) | 0.58 (0.12,2.76) | Voriconazole |

**S2.5.** Increase in creatinine - empirical /definitive therapy subgroup

| _Placebo_ | _LAmB_ | _Caspofungin_ | _Micafungin_ | _Itraconazole_ | _Voriconazole_ |
| --- | --- | --- | --- | --- | --- |
| Placebo | 3.20 (0.35,28.98) | 0.36 (0.03,3.80) | 1.44 (0.13,15.57) | 1.04 (0.06,16.76) | 1.62 (0.16,16.76) |
| 0.31 (0.03,2.83) | LAmB | 0.11 (0.05,0.26) | 0.45 (0.18,1.11) | 0.33 (0.06,1.77) | 0.51 (0.24,1.10) |
| 2.78 (0.26,29.39) | 8.90 (3.85,20.58) | Caspofungin | 4.01 (1.18,13.59) | 2.90 (0.44,19.15) | 4.52 (1.51,13.56) |
| 0.69 (0.06,7.50) | 2.22 (0.90,5.45) | 0.25 (0.07,0.85) | Micafungin | 0.72 (0.11,4.91) | 1.13 (0.42,3.05) |
| 0.96 (0.06,15.44) | 3.07 (0.57,16.67) | 0.34 (0.05,2.28) | 1.38 (0.20,9.39) | Itraconazole | 1.56 (0.24,10.00) |
| 0.62 (0.06,6.35) | 1.97 (0.91,4.25) | 0.22 (0.07,0.66) | 0.89 (0.33,2.40) | 0.64 (0.10,4.11) | Voriconazole |

**S2.6.** Increase in total or direct bilirubin

| _LAmB_ | _Caspofungin_ | _Micafungin_ | _Fluconazole_ | _Itraconazole_ | _Posaconazole_ | _Voriconazole_ |
| --- | --- | --- | --- | --- | --- | --- |
| LAmB | 0.57 (0.24,1.38) | 1.83 (0.46,7.20) | 0.95 (0.32,2.88) | 1.09 (0.53,2.26) | 1.37 (0.33,5.70) | 0.80 (0.31,2.05) |
| 1.75 (0.72,4.21) | Caspofungin | 3.19 (0.65,15.77) | 1.67 (0.44,6.37) | 1.91 (0.62,5.87) | 2.40 (0.47,12.23) | 1.40 (0.39,5.08) |
| 0.55 (0.14,2.16) | 0.31 (0.06,1.55) | Micafungin | 0.52 (0.16,1.65) | 0.60 (0.16,2.28) | 0.75 (0.16,3.53) | 0.44 (0.09,2.17) |
| 1.05 (0.35,3.17) | 0.60 (0.16,2.30) | 1.92 (0.61,6.07) | Fluconazole | 1.15 (0.37,3.57) | 1.44 (0.46,4.53) | 0.84 (0.20,3.46) |
| 0.92 (0.44,1.90) | 0.52 (0.17,1.61) | 1.67 (0.44,6.39) | 0.87 (0.28,2.72) | Itraconazole | 1.26 (0.31,5.08) | 0.73 (0.25,2.16) |
| 0.73 (0.18,3.02) | 0.42 (0.08,2.12) | 1.33 (0.28,6.24) | 0.69 (0.22,2.18) | 0.79 (0.20,3.21) | Posaconazole | 0.58 (0.11,3.07) |
| 1.25 (0.49,3.19) | 0.71 (0.20,2.59) | 2.28 (0.46,11.28) | 1.19 (0.29,4.90) | 1.36 (0.46,4.00) | 1.72 (0.33,9.02) | Voriconazole |

**S2.7.** Increase in total or direct bilirubin - empirical /definitive therapy subgroup

| _LAmB_ | _Caspofungin_ | _Micafungin_ | _Fluconazole_ | _Itraconazole_ | _Voriconazole_ |
| --- | --- | --- | --- | --- | --- |
| LAmB | 0.57 (0.27,1.22) | 5.37 (0.80,36.05) | 0.64 (0.18,2.33) | 0.93 (0.37,2.31) | 0.73 (0.37,1.44) |
| 1.76 (0.82,3.77) | Caspofungin | 9.45 (1.23,72.56) | 1.13 (0.26,4.89) | 1.63 (0.51,5.24) | 1.28 (0.46,3.57) |
| 0.19 (0.03,1.25) | 0.11 (0.01,0.81) | Micafungin | 0.12 (0.01,1.24) | 0.17 (0.03,0.92) | 0.14 (0.02,1.03) |
| 1.56 (0.43,5.67) | 0.89 (0.20,3.85) | 8.39 (0.81,87.14) | Fluconazole | 1.45 (0.28,7.45) | 1.14 (0.27,4.90) |
| 1.08 (0.43,2.68) | 0.61 (0.19,1.97) | 5.79 (1.09,30.82) | 0.69 (0.13,3.56) | Itraconazole | 0.79 (0.25,2.45) |
| 1.37 (0.69,2.71) | 0.78 (0.28,2.17) | 7.36 (0.97,55.56) | 0.88 (0.20,3.77) | 1.27 (0.41,3.96) | Voriconazole |

**S2.8.** Decrease in potassium

| _Placebo_ | _LAmB_ | _Caspofungin_ | _Micafungin_ | _Fluconazole_ | _Itraconazole_ | _Voriconazole_ |
| --- | --- | --- | --- | --- | --- | --- |
| Placebo | 0.17 (0.01,3.02) | 0.06 (0.00,1.11) | 0.06 (0.00,1.25) | 0.03 (0.00,0.61) | 0.07 (0.00,1.32) | 0.03 (0.00,0.65) |
| 6.00 (0.33,108.53) | LAmB | 0.33 (0.16,0.71) | 0.35 (0.12,0.98) | 0.18 (0.08,0.41) | 0.40 (0.19,0.85) | 0.17 (0.05,0.60) |
| 17.98 (0.90,358.69) | 3.00 (1.40,6.40) | Caspofungin | 1.04 (0.33,3.29) | 0.54 (0.20,1.48) | 1.19 (0.42,3.40) | 0.49 (0.11,2.19) |
| 17.33 (0.80,375.92) | 2.89 (1.02,8.19) | 0.96 (0.30,3.06) | Micafungin | 0.52 (0.19,1.47) | 1.15 (0.34,3.91) | 0.48 (0.09,2.47) |
| 33.00 (1.63,666.22) | 5.50 (2.46,12.30) | 1.84 (0.68,4.97) | 1.90 (0.68,5.34) | Fluconazole | 2.19 (0.84,5.69) | 0.91 (0.20,4.07) |
| 15.07 (0.76,300.89) | 2.51 (1.17,5.38) | 0.84 (0.29,2.39) | 0.87 (0.26,2.96) | 0.46 (0.18,1.19) | Itraconazole | 0.41 (0.09,1.84) |
| 36.36 (1.53,864.94) | 6.06 (1.67,21.99) | 2.02 (0.46,8.95) | 2.10 (0.41,10.86) | 1.10 (0.25,4.94) | 2.41 (0.54,10.68) | Voriconazole |

**S2.9.** Decrease in potassium - empirical /definitive therapy subgroup

| _Placebo_ | _LAmB_ | _Caspofungin_ | _Micafungin_ | _Fluconazole_ | _Itraconazole_ | _Voriconazole_ |
| --- | --- | --- | --- | --- | --- | --- |
| Placebo | 0.17 (0.01,2.08) | 0.06 (0.00,0.78) | 0.09 (0.01,1.22) | 0.04 (0.00,0.50) | 0.04 (0.00,0.49) | 0.06 (0.00,0.86) |
| 6.00 (0.48,74.77) | LAmB | 0.36 (0.22,0.57) | 0.53 (0.25,1.12) | 0.21 (0.09,0.48) | 0.21 (0.10,0.44) | 0.38 (0.19,0.75) |
| 16.77 (1.29,218.22) | 2.80 (1.75,4.46) | Caspofungin | 1.47 (0.64,3.38) | 0.59 (0.24,1.43) | 0.60 (0.26,1.40) | 1.06 (0.46,2.42) |
| 11.40 (0.82,158.53) | 1.90 (0.90,4.03) | 0.68 (0.30,1.56) | Micafungin | 0.40 (0.13,1.20) | 0.41 (0.14,1.15) | 0.72 (0.26,1.97) |
| 28.37 (2.00,401.89) | 4.73 (2.09,10.67) | 1.69 (0.70,4.10) | 2.49 (0.83,7.44) | Fluconazole | 1.01 (0.35,2.94) | 1.79 (0.64,4.97) |
| 28.00 (2.03,385.34) | 4.67 (2.28,9.54) | 1.67 (0.71,3.91) | 2.46 (0.87,6.93) | 0.99 (0.34,2.86) | Itraconazole | 1.76 (0.65,4.80) |
| 15.87 (1.16,216.57) | 2.64 (1.34,5.24) | 0.95 (0.41,2.17) | 1.39 (0.51,3.82) | 0.56 (0.20,1.55) | 0.57 (0.21,1.54) | Voriconazole |

**S2.10.** Decrease in potassium - prophylaxis therapy subgroup

| _LAmB_ | _Micafungin_ | _Fluconazole_ | _Itraconazole_ | _Voriconazole_ |
| --- | --- | --- | --- | --- |
| LAmB | 0.18 (0.02,2.19) | 0.14 (0.02,0.85) | 0.53 (0.13,2.17) | 0.02 (0.00,0.46) |
| 5.41 (0.46,64.00) | Micafungin | 0.78 (0.14,4.35) | 2.88 (0.25,33.40) | 0.13 (0.00,6.15) |
| 6.94 (1.18,40.96) | 1.28 (0.23,7.16) | Fluconazole | 3.70 (0.64,21.20) | 0.17 (0.01,5.27) |
| 1.88 (0.46,7.65) | 0.35 (0.03,4.03) | 0.27 (0.05,1.55) | Itraconazole | 0.04 (0.00,1.20) |
| 41.74 (2.15,810.11) | 7.72 (0.16,366.27) | 6.01 (0.19,190.61) | 22.23 (0.84,591.42) | Voriconazole |

**S2.11.** Increase in liver enzymes

| _Placebo_ | _LAmB_ | _Anidulafungin_ | _Caspofungin_ | _Micafungin_ | _Fluconazole_ | _Itraconazole_ | _Posaconazole_ | _Voriconazole_ |
| --- | --- | --- | --- | --- | --- | --- | --- | --- |
| Placebo | 1.32 (0.64,2.74) | 0.24 (0.04,1.44) | 0.77 (0.35,1.69) | 0.98 (0.42,2.33) | 1.37 (0.62,3.04) | 1.39 (0.71,2.72) | 2.55 (0.96,6.75) | 1.52 (0.69,3.35) |
| 0.75 (0.36,1.56) | LAmB | 0.18 (0.03,0.98) | 0.58 (0.39,0.86) | 0.74 (0.40,1.37) | 1.04 (0.62,1.75) | 1.05 (0.68,1.61) | 1.93 (0.87,4.26) | 1.15 (0.68,1.94) |
| 4.21 (0.70,25.48) | 5.58 (1.02,30.45) | Anidulafungin | 3.23 (0.59,17.69) | 4.15 (0.74,23.36) | 5.79 (1.15,29.09) | 5.85 (1.05,32.66) | 10.75 (1.89,61.24) | 6.42 (1.13,36.35) |
| 1.31 (0.59,2.88) | 1.73 (1.16,2.58) | 0.31 (0.06,1.70) | Caspofungin | 1.29 (0.70,2.36) | 1.79 (1.05,3.07) | 1.81 (1.06,3.11) | 3.33 (1.48,7.52) | 1.99 (1.08,3.66) |
| 1.02 (0.43,2.40) | 1.35 (0.73,2.49) | 0.24 (0.04,1.36) | 0.78 (0.42,1.43) | Micafungin | 1.40 (0.75,2.58) | 1.41 (0.76,2.63) | 2.59 (1.08,6.20) | 1.55 (0.76,3.16) |
| 0.73 (0.33,1.61) | 0.96 (0.57,1.62) | 0.17 (0.03,0.87) | 0.56 (0.33,0.95) | 0.72 (0.39,1.33) | Fluconazole | 1.01 (0.56,1.82) | 1.86 (0.97,3.55) | 1.11 (0.59,2.08) |
| 0.72 (0.37,1.41) | 0.95 (0.62,1.46) | 0.17 (0.03,0.95) | 0.55 (0.32,0.95) | 0.71 (0.38,1.32) | 0.99 (0.55,1.78) | Itraconazole | 1.84 (0.81,4.18) | 1.10 (0.61,1.97) |
| 0.39 (0.15,1.04) | 0.52 (0.23,1.15) | 0.09 (0.02,0.53) | 0.30 (0.13,0.68) | 0.39 (0.16,0.92) | 0.54 (0.28,1.03) | 0.54 (0.24,1.24) | Posaconazole | 0.60 (0.25,1.43) |
| 0.66 (0.30,1.44) | 0.87 (0.52,1.46) | 0.16 (0.03,0.88) | 0.50 (0.27,0.92) | 0.65 (0.32,1.32) | 0.90 (0.48,1.69) | 0.91 (0.51,1.64) | 1.67 (0.70,4.02) | Voriconazole |

**S2.12.** Increase in liver enzymes - empirical /definitive therapy subgroup

| _Placebo_ | _LAmB_ | _Anidulafungin_ | _Caspofungin_ | _Micafungin_ | _Fluconazole_ | _Itraconazole_ | _Posaconazole_ | _Voriconazole_ |
| --- | --- | --- | --- | --- | --- | --- | --- | --- |
| Placebo | 1.13 (0.25,5.13) | 0.20 (0.02,2.07) | 0.70 (0.14,3.36) | 1.20 (0.22,6.59) | 1.16 (0.24,5.71) | 0.92 (0.18,4.64) | 6.26 (0.28,140.61) | 1.33 (0.32,5.57) |
| 0.88 (0.19,4.00) | LAmB | 0.18 (0.03,1.11) | 0.62 (0.38,1.00) | 1.06 (0.46,2.41) | 1.03 (0.52,2.02) | 0.81 (0.43,1.52) | 5.53 (0.17,175.67) | 1.18 (0.59,2.32) |
| 4.98 (0.48,51.30) | 5.64 (0.90,35.30) | Anidulafungin | 3.47 (0.55,21.82) | 5.96 (0.86,41.35) | 5.79 (1.05,31.83) | 4.56 (0.67,31.11) | 31.17 (0.64,1522.92) | 6.63 (1.01,43.64) |
| 1.43 (0.30,6.91) | 1.62 (1.00,2.63) | 0.29 (0.05,1.81) | Caspofungin | 1.72 (0.77,3.80) | 1.67 (0.84,3.31) | 1.31 (0.60,2.85) | 8.97 (0.27,293.28) | 1.91 (0.87,4.17) |
| 0.84 (0.15,4.60) | 0.95 (0.42,2.15) | 0.17 (0.02,1.16) | 0.58 (0.26,1.29) | Micafungin | 0.97 (0.39,2.43) | 0.76 (0.29,2.02) | 5.23 (0.15,181.77) | 1.11 (0.41,3.03) |
| 0.86 (0.18,4.23) | 0.97 (0.50,1.92) | 0.17 (0.03,0.95) | 0.60 (0.30,1.19) | 1.03 (0.41,2.58) | Fluconazole | 0.79 (0.33,1.91) | 5.39 (0.16,177.55) | 1.15 (0.51,2.56) |
| 1.09 (0.22,5.53) | 1.24 (0.66,2.32) | 0.22 (0.03,1.50) | 0.76 (0.35,1.66) | 1.31 (0.50,3.45) | 1.27 (0.52,3.07) | Itraconazole | 6.84 (0.20,228.52) | 1.45 (0.59,3.58) |
| 0.16 (0.01,3.59) | 0.18 (0.01,5.75) | 0.03 (0.00,1.57) | 0.11 (0.00,3.64) | 0.19 (0.01,6.65) | 0.19 (0.01,6.12) | 0.15 (0.00,4.89) | Posaconazole | 0.21 (0.01,6.54) |
| 0.75 (0.18,3.14) | 0.85 (0.43,1.68) | 0.15 (0.02,0.99) | 0.52 (0.24,1.15) | 0.90 (0.33,2.45) | 0.87 (0.39,1.95) | 0.69 (0.28,1.70) | 4.70 (0.15,144.51) | Voriconazole |

**S2.13.** Increase in liver enzymes - prophylaxis therapy subgroup

| _Placebo_ | _LAmB_ | _Micafungin_ | _Fluconazole_ | _Itraconazole_ | _Posaconazole_ | _Voriconazole_ |
| --- | --- | --- | --- | --- | --- | --- |
| Placebo | 0.75 (0.23,2.43) | 0.56 (0.19,1.68) | 1.07 (0.35,3.28) | 1.44 (0.71,2.91) | 2.00 (0.61,6.50) | 2.15 (0.33,14.00) |
| 1.33 (0.41,4.32) | LAmB | 0.75 (0.20,2.80) | 1.43 (0.36,5.64) | 1.92 (0.75,4.92) | 2.66 (0.65,11.01) | 2.86 (0.40,20.65) |
| 1.78 (0.60,5.35) | 1.34 (0.36,5.02) | Micafungin | 1.91 (0.86,4.22) | 2.57 (1.02,6.50) | 3.57 (1.44,8.86) | 3.84 (0.54,27.48) |
| 0.94 (0.31,2.87) | 0.70 (0.18,2.78) | 0.52 (0.24,1.16) | Fluconazole | 1.35 (0.49,3.67) | 1.87 (1.14,3.08) | 2.01 (0.27,14.93) |
| 0.69 (0.34,1.41) | 0.52 (0.20,1.34) | 0.39 (0.15,0.99) | 0.74 (0.27,2.02) | Itraconazole | 1.39 (0.48,4.01) | 1.49 (0.26,8.47) |
| 0.50 (0.15,1.63) | 0.38 (0.09,1.55) | 0.28 (0.11,0.70) | 0.53 (0.32,0.88) | 0.72 (0.25,2.08) | Posaconazole | 1.08 (0.14,8.23) |
| 0.47 (0.07,3.03) | 0.35 (0.05,2.52) | 0.26 (0.04,1.87) | 0.50 (0.07,3.70) | 0.67 (0.12,3.80) | 0.93 (0.12,7.12) | Voriconazole |

**S2.14.** Nervous system disorders

| _Placebo_ | _LAmB_ | _Anidulafungin_ | _Caspofungin_ | _Micafungin_ | _Fluconazole_ | _Isavuconazole_ | _Itraconazole_ | _Posaconazole_ | _Voriconazole_ |
| --- | --- | --- | --- | --- | --- | --- | --- | --- | --- |
| Placebo | 0.71 (0.22,2.31) | 1.31 (0.26,6.65) | 0.58 (0.17,1.95) | 0.81 (0.24,2.72) | 0.98 (0.47,2.08) | 6.03 (1.09,33.55) | 1.13 (0.43,2.98) | 0.52 (0.15,1.78) | 8.66 (3.23,23.21) |
| 1.40 (0.43,4.55) | LAmB | 1.84 (0.32,10.57) | 0.81 (0.37,1.78) | 1.14 (0.33,3.94) | 1.38 (0.46,4.12) | 8.46 (1.53,46.80) | 1.58 (0.49,5.13) | 0.72 (0.16,3.28) | 12.15 (4.58,32.25) |
| 0.76 (0.15,3.87) | 0.54 (0.09,3.13) | Anidulafungin | 0.44 (0.07,2.64) | 0.62 (0.11,3.60) | 0.75 (0.17,3.31) | 4.60 (0.56,38.12) | 0.86 (0.16,4.50) | 0.39 (0.06,2.43) | 6.61 (1.36,32.09) |
| 1.73 (0.51,5.86) | 1.24 (0.56,2.72) | 2.27 (0.38,13.64) | Caspofungin | 1.41 (0.41,4.82) | 1.71 (0.54,5.38) | 10.46 (1.78,61.55) | 1.95 (0.55,6.90) | 0.89 (0.19,4.23) | 15.02 (5.10,44.26) |
| 1.23 (0.37,4.11) | 0.88 (0.25,3.03) | 1.61 (0.28,9.38) | 0.71 (0.21,2.43) | Micafungin | 1.21 (0.42,3.51) | 7.42 (1.25,44.05) | 1.39 (0.41,4.68) | 0.63 (0.14,2.84) | 10.66 (3.57,31.86) |
| 1.02 (0.48,2.15) | 0.72 (0.24,2.16) | 1.33 (0.30,5.88) | 0.59 (0.19,1.85) | 0.83 (0.28,2.40) | Fluconazole | 6.13 (1.15,32.55) | 1.14 (0.50,2.64) | 0.52 (0.18,1.50) | 8.80 (3.57,21.70) |
| 0.17 (0.03,0.92) | 0.12 (0.02,0.65) | 0.22 (0.03,1.80) | 0.10 (0.02,0.56) | 0.13 (0.02,0.80) | 0.16 (0.03,0.87) | Isavuconazole | 0.19 (0.03,1.04) | 0.09 (0.01,0.62) | 1.44 (0.35,5.85) |
| 0.89 (0.34,2.35) | 0.63 (0.19,2.06) | 1.16 (0.22,6.09) | 0.51 (0.14,1.81) | 0.72 (0.21,2.44) | 0.87 (0.38,2.01) | 5.36 (0.96,29.91) | Itraconazole | 0.46 (0.12,1.70) | 7.69 (2.85,20.76) |
| 1.94 (0.56,6.68) | 1.38 (0.30,6.26) | 2.54 (0.41,15.67) | 1.12 (0.24,5.29) | 1.57 (0.35,7.03) | 1.91 (0.67,5.44) | 11.69 (1.62,84.20) | 2.18 (0.59,8.08) | Posaconazole | 16.79 (4.19,67.24) |
| 0.12 (0.04,0.31) | 0.08 (0.03,0.22) | 0.15 (0.03,0.73) | 0.07 (0.02,0.20) | 0.09 (0.03,0.28) | 0.11 (0.05,0.28) | 0.70 (0.17,2.84) | 0.13 (0.05,0.35) | 0.06 (0.01,0.24) | Voriconazole |

**S2.15.** Nervous system disorders - empirical /definitive therapy subgroup

| _Placebo_ | _LAmB_ | _Anidulafungin_ | _Caspofungin_ | _Micafungin_ | _Fluconazole_ | _Isavuconazole_ | _Itraconazole_ | _Posaconazole_ | _Voriconazole_ |
| --- | --- | --- | --- | --- | --- | --- | --- | --- | --- |
| Placebo | 0.57 (0.13,2.57) | 1.32 (0.18,9.43) | 0.50 (0.11,2.24) | 0.71 (0.15,3.30) | 0.87 (0.25,3.05) | 7.62 (0.85,68.35) | 0.23 (0.03,1.62) | 0.63 (0.10,3.98) | 10.94 (3.01,39.77) |
| 1.75 (0.39,7.90) | LAmB | 2.31 (0.31,16.94) | 0.88 (0.35,2.19) | 1.25 (0.30,5.20) | 1.53 (0.40,5.84) | 13.35 (1.55,115.16) | 0.41 (0.07,2.54) | 1.10 (0.14,8.48) | 19.17 (5.65,65.11) |
| 0.76 (0.11,5.45) | 0.43 (0.06,3.19) | Anidulafungin | 0.38 (0.05,2.86) | 0.54 (0.08,3.84) | 0.66 (0.13,3.45) | 5.79 (0.47,71.17) | 0.18 (0.02,1.97) | 0.48 (0.05,4.74) | 8.31 (1.41,49.00) |
| 2.00 (0.45,8.99) | 1.14 (0.46,2.86) | 2.64 (0.35,19.83) | Caspofungin | 1.43 (0.35,5.77) | 1.75 (0.45,6.83) | 15.26 (1.70,137.25) | 0.47 (0.07,3.16) | 1.26 (0.16,9.80) | 21.91 (6.00,80.01) |
| 1.41 (0.30,6.52) | 0.80 (0.19,3.34) | 1.85 (0.26,13.14) | 0.70 (0.17,2.84) | Micafungin | 1.23 (0.35,4.30) | 10.71 (1.22,93.84) | 0.33 (0.05,2.18) | 0.88 (0.12,6.57) | 15.37 (4.40,53.70) |
| 1.14 (0.33,3.99) | 0.65 (0.17,2.48) | 1.50 (0.29,7.82) | 0.57 (0.15,2.23) | 0.81 (0.23,2.84) | Fluconazole | 8.71 (1.06,71.64) | 0.27 (0.04,1.78) | 0.72 (0.14,3.66) | 12.51 (4.01,38.98) |
| 0.13 (0.01,1.18) | 0.07 (0.01,0.65) | 0.17 (0.01,2.12) | 0.07 (0.01,0.59) | 0.09 (0.01,0.82) | 0.11 (0.01,0.94) | Isavuconazole | 0.03 (0.00,0.39) | 0.08 (0.01,1.12) | 1.44 (0.24,8.47) |
| 4.27 (0.62,29.66) | 2.44 (0.39,15.12) | 5.63 (0.51,62.24) | 2.13 (0.32,14.41) | 3.04 (0.46,20.14) | 3.74 (0.56,24.83) | 32.57 (2.57,412.39) | Itraconazole | 2.69 (0.24,30.37) | 46.77 (7.61,287.38) |
| 1.59 (0.25,10.06) | 0.91 (0.12,6.97) | 2.09 (0.21,20.74) | 0.79 (0.10,6.17) | 1.13 (0.15,8.39) | 1.39 (0.27,7.07) | 12.11 (0.89,163.90) | 0.37 (0.03,4.19) | Posaconazole | 17.38 (2.58,117.18) |
| 0.09 (0.03,0.33) | 0.05 (0.02,0.18) | 0.12 (0.02,0.71) | 0.05 (0.01,0.17) | 0.07 (0.02,0.23) | 0.08 (0.03,0.25) | 0.70 (0.12,4.11) | 0.02 (0.00,0.13) | 0.06 (0.01,0.39) | Voriconazole |

**S2.16** Nervous system disorders - prophylaxis therapy subgroup

| _Placebo_ | _LAmB_ | _Fluconazole_ | _Itraconazole_ | _Posaconazole_ | _Voriconazole_ |
| --- | --- | --- | --- | --- | --- |
| Placebo | 0.37 (0.03,4.12) | 1.05 (0.57,1.94) | 1.27 (0.53,3.06) | 0.36 (0.09,1.43) | 2.95 (0.96,9.07) |
| 2.71 (0.24,30.23) | LAmB | 2.83 (0.27,30.25) | 3.45 (0.36,32.71) | 0.98 (0.07,14.01) | 7.98 (0.94,67.46) |
| 0.96 (0.52,1.77) | 0.35 (0.03,3.77) | Fluconazole | 1.22 (0.58,2.56) | 0.35 (0.10,1.18) | 2.81 (1.01,7.83) |
| 0.78 (0.33,1.88) | 0.29 (0.03,2.75) | 0.82 (0.39,1.72) | Itraconazole | 0.28 (0.07,1.17) | 2.31 (1.14,4.68) |
| 2.76 (0.70,10.88) | 1.02 (0.07,14.57) | 2.89 (0.85,9.87) | 3.52 (0.85,14.55) | Posaconazole | 8.13 (1.67,39.70) |
| 0.34 (0.11,1.04) | 0.13 (0.01,1.06) | 0.36 (0.13,0.99) | 0.43 (0.21,0.88) | 0.12 (0.03,0.60) | Voriconazole |

**S2.17.** Vascular disorders

| _Placebo_ | _LAmB_ | _Anidulafungin_ | _Caspofungin_ | _Micafungin_ | _Fluconazole_ | _Voriconazole_ |
| --- | --- | --- | --- | --- | --- | --- |
| Placebo | 82.87 (0.91,7587.36) | 21.39 (0.20,2299.01) | 46.69 (0.53,4118.76) | 31.25 (0.39,2497.36) | 21.32 (0.25,1851.82) | 5.34 (0.24,120.99) |
| 0.01 (0.00,1.10) | LAmB | 0.26 (0.05,1.41) | 0.56 (0.31,1.03) | 0.38 (0.13,1.13) | 0.26 (0.10,0.68) | 0.06 (0.00,1.69) |
| 0.05 (0.00,5.03) | 3.87 (0.71,21.19) | Anidulafungin | 2.18 (0.44,10.84) | 1.46 (0.28,7.52) | 1.00 (0.25,4.02) | 0.25 (0.01,8.15) |
| 0.02 (0.00,1.89) | 1.77 (0.97,3.24) | 0.46 (0.09,2.28) | Caspofungin | 0.67 (0.26,1.71) | 0.46 (0.21,1.01) | 0.11 (0.00,2.85) |
| 0.03 (0.00,2.56) | 2.65 (0.88,7.97) | 0.68 (0.13,3.52) | 1.49 (0.59,3.81) | Micafungin | 0.68 (0.29,1.61) | 0.17 (0.01,3.71) |
| 0.05 (0.00,4.08) | 3.89 (1.47,10.26) | 1.00 (0.25,4.05) | 2.19 (0.99,4.82) | 1.47 (0.62,3.46) | Fluconazole | 0.25 (0.01,6.11) |
| 0.19 (0.01,4.24) | 15.51 (0.59,406.62) | 4.00 (0.12,130.57) | 8.74 (0.35,217.56) | 5.85 (0.27,126.70) | 3.99 (0.16,97.23) | Voriconazole |

**S2.18.** Skin and subcutaneous tissue disorders

| _Placebo_ | _LAmB_ | _Caspofungin_ | _Micafungin_ | _Fluconazole_ | _Isavuconazole_ | _Itraconazole_ | _Posaconazole_ | _Voriconazole_ |
| --- | --- | --- | --- | --- | --- | --- | --- | --- |
| Placebo | 1.88 (0.82,4.29) | 1.61 (0.57,4.54) | 2.63 (0.95,7.27) | 1.22 (0.70,2.14) | 1.99 (0.42,9.51) | 1.33 (0.68,2.60) | 0.58 (0.10,3.42) | 2.93 (1.12,7.67) |
| 0.53 (0.23,1.22) | LAmB | 0.86 (0.39,1.90) | 1.40 (0.51,3.83) | 0.65 (0.32,1.32) | 1.06 (0.23,5.00) | 0.71 (0.33,1.51) | 0.31 (0.05,1.92) | 1.56 (0.61,4.01) |
| 0.62 (0.22,1.75) | 1.16 (0.53,2.57) | Caspofungin | 1.63 (0.54,4.91) | 0.76 (0.30,1.93) | 1.24 (0.23,6.60) | 0.82 (0.30,2.28) | 0.36 (0.05,2.47) | 1.82 (0.58,5.65) |
| 0.38 (0.14,1.06) | 0.71 (0.26,1.96) | 0.61 (0.20,1.85) | Micafungin | 0.47 (0.20,1.10) | 0.76 (0.15,3.80) | 0.50 (0.18,1.42) | 0.22 (0.03,1.46) | 1.12 (0.39,3.15) |
| 0.82 (0.47,1.43) | 1.54 (0.76,3.12) | 1.32 (0.52,3.37) | 2.15 (0.91,5.08) | Fluconazole | 1.63 (0.37,7.25) | 1.09 (0.55,2.13) | 0.47 (0.09,2.56) | 2.40 (1.03,5.57) |
| 0.50 (0.11,2.39) | 0.94 (0.20,4.42) | 0.81 (0.15,4.30) | 1.32 (0.26,6.58) | 0.61 (0.14,2.72) | Isavuconazole | 0.66 (0.14,3.17) | 0.29 (0.03,2.75) | 1.47 (0.43,5.02) |
| 0.75 (0.39,1.48) | 1.42 (0.66,3.02) | 1.22 (0.44,3.37) | 1.98 (0.70,5.59) | 0.92 (0.47,1.81) | 1.51 (0.32,7.19) | Itraconazole | 0.43 (0.07,2.69) | 2.21 (0.84,5.81) |
| 1.74 (0.29,10.35) | 3.26 (0.52,20.44) | 2.80 (0.40,19.39) | 4.56 (0.68,30.48) | 2.12 (0.39,11.54) | 3.47 (0.36,33.09) | 2.30 (0.37,14.27) | Posaconazole | 5.09 (0.77,33.75) |
| 0.34 (0.13,0.90) | 0.64 (0.25,1.64) | 0.55 (0.18,1.71) | 0.90 (0.32,2.53) | 0.42 (0.18,0.97) | 0.68 (0.20,2.33) | 0.45 (0.17,1.19) | 0.20 (0.03,1.30) | Voriconazole |

**S2.19.** Skin and subcutaneous tissue disorders - empirical /definitive therapy subgroup

| _Placebo_ | _LAmB_ | _Caspofungin_ | _Micafungin_ | _Fluconazole_ | _Isavuconazole_ | _Itraconazole_ | _Posaconazole_ | _Voriconazole_ |
| --- | --- | --- | --- | --- | --- | --- | --- | --- |
| Placebo | 0.06 (0.00,1.09) | 1.07 (0.01,120.40) | 13.52 (0.35,524.27) | 0.14 (0.00,8.49) | 30.81 (1.96,483.33) | 26.00 (1.83,368.92) | 0.84 (0.42,1.67) | 1.97 (0.16,23.69) |
| 17.17 (0.91,322.44) | LAmB | 0.84 (0.40,1.76) | 5.49 (0.98,30.84) | 0.03 (0.00,0.46) | 0.06 (0.00,2.61) | 0.06 (0.00,2.52) | 0.01 (0.00,0.28) | 0.09 (0.00,3.76) |
| 0.93 (0.01,105.07) | 1.18 (0.57,2.48) | Caspofungin | 6.51 (1.37,30.97) | 0.03 (0.00,0.50) | 0.08 (0.00,2.87) | 0.07 (0.00,2.77) | 0.02 (0.00,0.31) | 0.11 (0.00,4.14) |
| 0.07 (0.00,2.87) | 0.18 (0.03,1.02) | 0.15 (0.03,0.73) | Micafungin | 0.00 (0.00,0.12) | 0.01 (0.00,0.61) | 0.01 (0.00,0.59) | 0.00 (0.00,0.07) | 0.02 (0.00,0.88) |
| 7.35 (0.12,458.20) | 36.79 (2.18,621.12) | 31.05 (2.00,481.08) | 202.15 (8.64,4732.11) | Fluconazole | 2.35 (0.18,31.22) | 2.24 (0.17,30.09) | 0.47 (0.14,1.61) | 3.45 (0.27,44.54) |
| 0.03 (0.00,0.51) | 15.64 (0.38,639.10) | 13.20 (0.35,500.73) | 85.92 (1.64,4490.86) | 0.43 (0.03,5.64) | Isavuconazole | 0.95 (0.03,32.48) | 0.20 (0.01,3.50) | 1.47 (1.00,2.15) |
| 0.04 (0.00,0.55) | 16.43 (0.40,679.49) | 13.87 (0.36,532.74) | 90.27 (1.71,4773.12) | 0.45 (0.03,6.00) | 1.05 (0.03,35.85) | Itraconazole | 0.21 (0.01,3.72) | 1.54 (0.05,51.55) |
| 1.19 (0.60,2.38) | 78.07 (3.58,1700.11) | 65.89 (3.27,1326.44) | 428.95 (14.56,12639.05) | 2.12 (0.62,7.23) | 4.99 (0.29,87.30) | 4.75 (0.27,84.06) | Posaconazole | 7.33 (0.43,124.92) |
| 0.51 (0.04,6.10) | 10.65 (0.27,426.89) | 8.99 (0.24,334.33) | 58.53 (1.14,3003.40) | 0.29 (0.02,3.73) | 0.68 (0.47,1.00) | 0.65 (0.02,21.67) | 0.14 (0.01,2.33) | Voriconazole |

**S2.20.** Skin and subcutaneous tissue disorders - prophylaxis therapy subgroup

| _Placebo_ | _LAmB_ | _Micafungin_ | _Fluconazole_ | _Itraconazole_ | _Voriconazole_ |
| --- | --- | --- | --- | --- | --- |
| Placebo | 1.40 (0.35,5.68) | 1.65 (0.22,12.39) | 1.14 (0.50,2.59) | 1.42 (0.57,3.53) | 17.13 (0.48,609.15) |
| 0.71 (0.18,2.89) | LAmB | 1.18 (0.12,11.67) | 0.82 (0.21,3.21) | 1.01 (0.32,3.19) | 12.21 (0.46,326.45) |
| 0.61 (0.08,4.55) | 0.85 (0.09,8.45) | Micafungin | 0.69 (0.11,4.38) | 0.86 (0.11,6.81) | 10.38 (0.19,571.83) |
| 0.87 (0.39,1.98) | 1.23 (0.31,4.82) | 1.44 (0.23,9.10) | Fluconazole | 1.24 (0.48,3.19) | 14.98 (0.43,526.55) |
| 0.71 (0.28,1.76) | 0.99 (0.31,3.13) | 1.16 (0.15,9.24) | 0.81 (0.31,2.08) | Itraconazole | 12.10 (0.37,393.38) |
| 0.06 (0.00,2.08) | 0.08 (0.00,2.19) | 0.10 (0.00,5.30) | 0.07 (0.00,2.35) | 0.08 (0.00,2.69) | Voriconazole |

**S2.21.** Respiratory, thoracic, and mediastinal disorders

| _Placebo_ | _LAmB_ | _Caspofungin_ | _Fluconazole_ | _Isavuconazole_ | _Itraconazole_ | _Micafungin_ | _Voriconazole_ |
| --- | --- | --- | --- | --- | --- | --- | --- |
| Placebo | 3.88 (1.11,13.60) | 1.25 (0.23,6.81) | 1.28 (0.83,1.96) | 0.31 (0.06,1.48) | 3.26 (0.94,11.28) | 0.62 (0.36,1.08) | 0.32 (0.07,1.49) |
| 0.26 (0.07,0.90) | LAmB | 0.32 (0.10,1.01) | 0.33 (0.09,1.16) | 0.08 (0.03,0.24) | 0.84 (0.51,1.39) | 0.16 (0.04,0.63) | 0.08 (0.03,0.24) |
| 0.80 (0.15,4.37) | 3.11 (0.99,9.73) | Caspofungin | 1.02 (0.19,5.59) | 0.24 (0.05,1.20) | 2.61 (0.75,9.10) | 0.50 (0.08,2.97) | 0.26 (0.05,1.21) |
| 0.78 (0.51,1.20) | 3.04 (0.86,10.69) | 0.98 (0.18,5.35) | Fluconazole | 0.24 (0.05,1.16) | 2.55 (0.73,8.93) | 0.49 (0.24,0.98) | 0.25 (0.05,1.16) |
| 3.27 (0.68,15.89) | 12.70 (4.21,38.37) | 4.09 (0.83,20.03) | 4.18 (0.86,20.22) | Isavuconazole | 10.68 (3.20,35.61) | 2.04 (0.38,10.88) | 1.05 (0.74,1.48) |
| 0.31 (0.09,1.06) | 1.19 (0.72,1.97) | 0.38 (0.11,1.33) | 0.39 (0.11,1.37) | 0.09 (0.03,0.31) | Itraconazole | 0.19 (0.05,0.74) | 0.10 (0.03,0.31) |
| 1.60 (0.92,2.78) | 6.22 (1.58,24.47) | 2.00 (0.34,11.91) | 2.05 (1.02,4.12) | 0.49 (0.09,2.61) | 5.23 (1.35,20.32) | Micafungin | 0.51 (0.10,2.63) |
| 3.13 (0.67,14.61) | 12.14 (4.25,34.66) | 3.91 (0.83,18.42) | 4.00 (0.86,18.59) | 0.96 (0.67,1.35) | 10.21 (3.22,32.33) | 1.95 (0.38,10.02) | Voriconazole |

**S2.22.** Respiratory, thoracic, and mediastinal disorders - empirical /definitive therapy subgroup

| _Placebo_ | _LAmB_ | _Caspofungin_ | _Fluconazole_ | _Isavuconazole_ | _Itraconazole_ | _Voriconazole_ |
| --- | --- | --- | --- | --- | --- | --- |
| Placebo | 2.58 (0.61,10.81) | 0.83 (0.13,5.19) | 1.15 (0.72,1.84) | 0.21 (0.04,1.14) | 2.52 (0.65,9.80) | 0.22 (0.04,1.15) |
| 0.39 (0.09,1.63) | LAmB | 0.32 (0.10,1.01) | 0.45 (0.10,1.91) | 0.08 (0.03,0.25) | 0.98 (0.45,2.13) | 0.08 (0.03,0.24) |
| 1.21 (0.19,7.54) | 3.11 (0.99,9.73) | Caspofungin | 1.39 (0.22,8.79) | 0.25 (0.05,1.24) | 3.04 (0.76,12.09) | 0.26 (0.06,1.25) |
| 0.87 (0.54,1.39) | 2.24 (0.52,9.57) | 0.72 (0.11,4.57) | Fluconazole | 0.18 (0.03,1.00) | 2.19 (0.55,8.79) | 0.19 (0.04,1.01) |
| 4.78 (0.87,26.13) | 12.32 (4.07,37.29) | 3.97 (0.81,19.46) | 5.50 (1.00,30.25) | Isavuconazole | 12.05 (3.21,45.27) | 1.05 (0.74,1.48) |
| 0.40 (0.10,1.54) | 1.02 (0.47,2.22) | 0.33 (0.08,1.31) | 0.46 (0.11,1.83) | 0.08 (0.02,0.31) | Itraconazole | 0.09 (0.02,0.31) |
| 4.57 (0.87,24.09) | 11.77 (4.11,33.70) | 3.79 (0.80,17.89) | 5.25 (0.99,27.90) | 0.96 (0.67,1.35) | 11.52 (3.21,41.30) | Voriconazole |

**S2.23.** Renal and urinary disorders

| _Placebo_ | _LAmB_ | _Caspofungin_ | _Micafungin_ | _Fluconazole_ | _Isavuconazole_ | _Itraconazole_ | _Voriconazole_ |
| --- | --- | --- | --- | --- | --- | --- | --- |
| Placebo | 6.49 (0.32,130.98) | 2.08 (0.09,50.21) | 1.68 (0.06,48.25) | 1.31 (0.06,31.13) | 1.02 (0.06,18.09) | 0.81 (0.03,20.27) | 1.08 (0.09,13.15) |
| 0.15 (0.01,3.11) | LAmB | 0.32 (0.11,0.94) | 0.26 (0.03,2.04) | 0.20 (0.07,0.61) | 0.16 (0.02,1.40) | 0.12 (0.04,0.39) | 0.17 (0.03,0.87) |
| 0.48 (0.02,11.64) | 3.12 (1.06,9.19) | Caspofungin | 0.81 (0.08,8.39) | 0.63 (0.13,3.03) | 0.49 (0.04,5.56) | 0.39 (0.08,1.89) | 0.52 (0.07,3.72) |
| 0.59 (0.02,17.05) | 3.86 (0.49,30.38) | 1.23 (0.12,12.79) | Micafungin | 0.78 (0.11,5.41) | 0.60 (0.04,8.56) | 0.48 (0.05,4.77) | 0.64 (0.07,5.99) |
| 0.76 (0.03,18.04) | 4.94 (1.63,14.94) | 1.58 (0.33,7.58) | 1.28 (0.18,8.88) | Fluconazole | 0.77 (0.07,8.56) | 0.61 (0.14,2.62) | 0.82 (0.12,5.69) |
| 0.98 (0.06,17.52) | 6.39 (0.72,57.00) | 2.04 (0.18,23.24) | 1.66 (0.12,23.46) | 1.29 (0.12,14.31) | Isavuconazole | 0.79 (0.07,9.49) | 1.06 (0.26,4.40) |
| 1.24 (0.05,31.21) | 8.06 (2.59,25.07) | 2.58 (0.53,12.55) | 2.09 (0.21,20.78) | 1.63 (0.38,6.97) | 1.26 (0.11,15.10) | Itraconazole | 1.34 (0.17,10.22) |
| 0.93 (0.08,11.34) | 6.03 (1.14,31.78) | 1.93 (0.27,13.84) | 1.56 (0.17,14.62) | 1.22 (0.18,8.47) | 0.94 (0.23,3.92) | 0.75 (0.10,5.72) | Voriconazole |

**S2.24.** Renal and urinary disorders - empirical /definitive therapy subgroup

| _Placebo_ | _LAmB_ | _Caspofungin_ | _Micafungin_ | _Fluconazole_ | _Isavuconazole_ | _Itraconazole_ | _Voriconazole_ |
| --- | --- | --- | --- | --- | --- | --- | --- |
| Placebo | 11.83 (0.91,153.78) | 3.53 (0.25,49.92) | 1.08 (0.03,35.67) | 5.57 (0.38,82.59) | 1.02 (0.12,8.65) | 1.09 (0.06,21.10) | 1.08 (0.13,8.80) |
| 0.08 (0.01,1.10) | LAmB | 0.30 (0.15,0.58) | 0.09 (0.00,2.15) | 0.47 (0.20,1.08) | 0.09 (0.02,0.40) | 0.09 (0.02,0.41) | 0.09 (0.02,0.40) |
| 0.28 (0.02,4.00) | 3.35 (1.73,6.48) | Caspofungin | 0.30 (0.01,7.71) | 1.58 (0.55,4.56) | 0.29 (0.05,1.52) | 0.31 (0.06,1.56) | 0.30 (0.06,1.53) |
| 0.93 (0.03,30.75) | 10.99 (0.46,259.82) | 3.28 (0.13,83.06) | Micafungin | 5.17 (0.20,136.24) | 0.94 (0.06,16.00) | 1.01 (0.03,33.33) | 1.00 (0.06,16.44) |
| 0.18 (0.01,2.66) | 2.12 (0.92,4.88) | 0.63 (0.22,1.83) | 0.19 (0.01,5.09) | Fluconazole | 0.18 (0.03,1.04) | 0.20 (0.04,1.07) | 0.19 (0.04,1.05) |
| 0.98 (0.12,8.38) | 11.64 (2.52,53.77) | 3.48 (0.66,18.40) | 1.06 (0.06,17.97) | 5.48 (0.96,31.29) | Isavuconazole | 1.08 (0.13,9.03) | 1.06 (0.70,1.61) |
| 0.91 (0.05,17.66) | 10.83 (2.47,47.48) | 3.23 (0.64,16.32) | 0.99 (0.03,32.36) | 5.10 (0.93,27.81) | 0.93 (0.11,7.80) | Itraconazole | 0.99 (0.12,7.93) |
| 0.93 (0.11,7.59) | 10.99 (2.52,47.87) | 3.28 (0.65,16.46) | 1.00 (0.06,16.44) | 5.17 (0.95,28.06) | 0.94 (0.62,1.43) | 1.01 (0.13,8.17) | Voriconazole |

**S2.25.** Renal and urinary disorders - prophylaxis therapy subgroup

| _LAmB_ | _Micafungin_ | _Fluconazole_ | _Itraconazole_ | _Voriconazole_ |
| --- | --- | --- | --- | --- |
| LAmB | 0.07 (0.00,12.08) | 0.04 (0.00,1.19) | 0.07 (0.00,1.79) | 1.00 (0.00,200.06) |
| 15.15 (0.08,2771.52) | Micafungin | 0.62 (0.01,32.82) | 1.05 (0.00,246.74) | 15.15 (0.01,25549.64) |
| 24.50 (0.84,712.73) | 1.62 (0.03,85.86) | Fluconazole | 1.70 (0.04,71.83) | 24.50 (0.05,13074.82) |
| 14.40 (0.56,371.27) | 0.95 (0.00,222.88) | 0.59 (0.01,24.81) | Itraconazole | 14.40 (0.03,7208.12) |
| 1.00 (0.00,200.06) | 0.07 (0.00,111.32) | 0.04 (0.00,21.78) | 0.07 (0.00,34.77) | Voriconazole |

**S2.26.** Hepatobiliary disorders

| _Placebo_ | _LAmB_ | _Caspofungin_ | _Micafungin_ | _Fluconazole_ | _Isavuconazole_ | _Itraconazole_ | _Posaconazole_ | _Voriconazole_ |
| --- | --- | --- | --- | --- | --- | --- | --- | --- |
| Placebo | 0.89 (0.35,2.24) | 0.16 (0.05,0.54) | 0.67 (0.38,1.19) | 0.71 (0.39,1.27) | 0.93 (0.37,2.34) | 0.76 (0.38,1.50) | 1.31 (0.33,5.13) | 1.84 (0.87,3.87) |
| 1.12 (0.45,2.83) | LAmB | 0.18 (0.08,0.43) | 0.75 (0.35,1.63) | 0.79 (0.37,1.72) | 1.05 (0.42,2.62) | 0.85 (0.42,1.73) | 1.47 (0.35,6.23) | 2.07 (0.99,4.31) |
| 6.22 (1.87,20.74) | 5.53 (2.35,13.05) | Caspofungin | 4.16 (1.40,12.38) | 4.39 (1.47,13.18) | 5.81 (1.73,19.48) | 4.72 (1.63,13.68) | 8.13 (1.57,42.06) | 11.44 (3.88,33.75) |
| 1.49 (0.84,2.66) | 1.33 (0.61,2.88) | 0.24 (0.08,0.71) | Micafungin | 1.06 (0.71,1.58) | 1.40 (0.66,2.97) | 1.13 (0.70,1.84) | 1.95 (0.53,7.14) | 2.75 (1.62,4.65) |
| 1.42 (0.79,2.55) | 1.26 (0.58,2.73) | 0.23 (0.08,0.68) | 0.95 (0.63,1.42) | Fluconazole | 1.32 (0.60,2.91) | 1.07 (0.69,1.66) | 1.85 (0.54,6.39) | 2.60 (1.47,4.62) |
| 1.07 (0.43,2.69) | 0.95 (0.38,2.37) | 0.17 (0.05,0.58) | 0.72 (0.34,1.52) | 0.76 (0.34,1.66) | Isavuconazole | 0.81 (0.39,1.71) | 1.40 (0.33,6.00) | 1.97 (1.15,3.38) |
| 1.32 (0.67,2.60) | 1.17 (0.58,2.38) | 0.21 (0.07,0.61) | 0.88 (0.54,1.43) | 0.93 (0.60,1.44) | 1.23 (0.58,2.60) | Itraconazole | 1.72 (0.47,6.26) | 2.42 (1.45,4.05) |
| 0.77 (0.19,3.00) | 0.68 (0.16,2.89) | 0.12 (0.02,0.64) | 0.51 (0.14,1.87) | 0.54 (0.16,1.87) | 0.71 (0.17,3.06) | 0.58 (0.16,2.11) | Posaconazole | 1.41 (0.36,5.43) |
| 0.54 (0.26,1.15) | 0.48 (0.23,1.01) | 0.09 (0.03,0.26) | 0.36 (0.22,0.62) | 0.38 (0.22,0.68) | 0.51 (0.30,0.87) | 0.41 (0.25,0.69) | 0.71 (0.18,2.74) | Voriconazole |

**S2.27.** Hepatobiliary disorders - empirical /definitive therapy subgroup

| _Placebo_ | _LAmB_ | _Caspofungin_ | _Micafungin_ | _Fluconazole_ | _Isavuconazole_ | _Itraconazole_ | _Voriconazole_ |
| --- | --- | --- | --- | --- | --- | --- | --- |
| Placebo | 3.64 (0.24,55.03) | 0.64 (0.04,10.35) | 2.20 (0.20,24.56) | 0.73 (0.32,1.66) | 2.73 (0.21,35.32) | 2.94 (0.16,52.73) | 5.38 (0.44,65.65) |
| 0.27 (0.02,4.15) | LAmB | 0.18 (0.07,0.42) | 0.60 (0.17,2.10) | 0.20 (0.02,2.68) | 0.75 (0.21,2.62) | 0.81 (0.30,2.14) | 1.48 (0.48,4.57) |
| 1.56 (0.10,25.22) | 5.69 (2.39,13.52) | Caspofungin | 3.43 (0.86,13.73) | 1.14 (0.08,16.35) | 4.26 (1.03,17.68) | 4.60 (1.25,16.95) | 8.39 (2.25,31.29) |
| 0.45 (0.04,5.07) | 1.66 (0.48,5.76) | 0.29 (0.07,1.16) | Micafungin | 0.33 (0.03,3.23) | 1.24 (0.53,2.92) | 1.34 (0.28,6.51) | 2.44 (1.26,4.75) |
| 1.36 (0.60,3.09) | 4.97 (0.37,66.15) | 0.87 (0.06,12.48) | 3.00 (0.31,29.03) | Fluconazole | 3.72 (0.33,42.13) | 4.02 (0.25,63.88) | 7.33 (0.69,78.04) |
| 0.37 (0.03,4.74) | 1.33 (0.38,4.67) | 0.23 (0.06,0.97) | 0.81 (0.34,1.90) | 0.27 (0.02,3.04) | Isavuconazole | 1.08 (0.22,5.28) | 1.97 (1.15,3.38) |
| 0.34 (0.02,6.08) | 1.24 (0.47,3.28) | 0.22 (0.06,0.80) | 0.75 (0.15,3.63) | 0.25 (0.02,3.96) | 0.93 (0.19,4.53) | Itraconazole | 1.83 (0.41,8.12) |
| 0.19 (0.02,2.27) | 0.68 (0.22,2.10) | 0.12 (0.03,0.44) | 0.41 (0.21,0.80) | 0.14 (0.01,1.45) | 0.51 (0.30,0.87) | 0.55 (0.12,2.44) | Voriconazole |

**S2.28.** Hepatobiliary disorders - prophylaxis therapy subgroup

| _Placebo_ | _LAmB_ | _Micafungin_ | _Fluconazole_ | _Itraconazole_ | _Posaconazole_ | _Voriconazole_ |
| --- | --- | --- | --- | --- | --- | --- |
| Placebo | 0.45 (0.07,2.96) | 0.64 (0.31,1.35) | 0.73 (0.31,1.73) | 0.83 (0.33,2.09) | 1.36 (0.30,6.13) | 2.31 (0.74,7.25) |
| 2.23 (0.34,14.68) | LAmB | 1.44 (0.25,8.21) | 1.62 (0.30,8.83) | 1.85 (0.34,10.05) | 3.03 (0.37,24.53) | 5.15 (0.96,27.62) |
| 1.55 (0.74,3.24) | 0.70 (0.12,3.98) | Micafungin | 1.13 (0.71,1.80) | 1.29 (0.72,2.32) | 2.11 (0.56,7.90) | 3.59 (1.47,8.77) |
| 1.37 (0.58,3.25) | 0.62 (0.11,3.36) | 0.89 (0.55,1.41) | Fluconazole | 1.14 (0.71,1.83) | 1.87 (0.54,6.46) | 3.18 (1.40,7.21) |
| 1.20 (0.48,3.03) | 0.54 (0.10,2.94) | 0.78 (0.43,1.40) | 0.88 (0.55,1.41) | Itraconazole | 1.64 (0.45,6.01) | 2.79 (1.41,5.52) |
| 0.73 (0.16,3.31) | 0.33 (0.04,2.67) | 0.47 (0.13,1.77) | 0.54 (0.15,1.85) | 0.61 (0.17,2.24) | Posaconazole | 1.70 (0.39,7.35) |
| 0.43 (0.14,1.35) | 0.19 (0.04,1.04) | 0.28 (0.11,0.68) | 0.31 (0.14,0.72) | 0.36 (0.18,0.71) | 0.59 (0.14,2.54) | Voriconazole |

**S2.29.** General disorders and administrative site conditions

| _Placebo_ | _LAmB_ | _Anidulafungin_ | _Caspofungin_ | _Micafungin_ | _Fluconazole_ | _Isavuconazole_ | _Itraconazole_ | _Posaconazole_ | _Voriconazole_ |
| --- | --- | --- | --- | --- | --- | --- | --- | --- | --- |
| Placebo | 5.10 (1.80,14.45) | 0.51 (0.03,9.88) | 0.86 (0.24,3.10) | 1.45 (0.47,4.51) | 0.76 (0.32,1.78) | 4.63 (0.58,36.92) | 1.45 (0.53,3.97) | 0.95 (0.16,5.71) | 2.24 (0.70,7.15) |
| 0.20 (0.07,0.56) | LAmB | 0.10 (0.01,1.95) | 0.17 (0.06,0.46) | 0.28 (0.09,0.86) | 0.15 (0.06,0.36) | 0.91 (0.12,6.86) | 0.28 (0.11,0.72) | 0.19 (0.03,1.17) | 0.44 (0.15,1.28) |
| 1.98 (0.10,38.63) | 10.07 (0.51,198.21) | Anidulafungin | 1.70 (0.08,36.72) | 2.86 (0.14,58.57) | 1.50 (0.09,25.87) | 9.15 (0.28,303.89) | 2.87 (0.14,57.19) | 1.88 (0.07,51.31) | 4.44 (0.21,93.63) |
| 1.16 (0.32,4.21) | 5.93 (2.16,16.32) | 0.59 (0.03,12.74) | Caspofungin | 1.69 (0.46,6.23) | 0.88 (0.28,2.81) | 5.39 (0.59,49.39) | 1.69 (0.48,5.94) | 1.11 (0.15,8.22) | 2.61 (0.65,10.45) |
| 0.69 (0.22,2.15) | 3.52 (1.17,10.59) | 0.35 (0.02,7.14) | 0.59 (0.16,2.19) | Micafungin | 0.52 (0.19,1.42) | 3.19 (0.36,28.41) | 1.00 (0.32,3.13) | 0.66 (0.10,4.45) | 1.55 (0.40,5.94) |
| 1.32 (0.56,3.09) | 6.72 (2.80,16.13) | 0.67 (0.04,11.50) | 1.13 (0.36,3.60) | 1.91 (0.70,5.19) | Fluconazole | 6.10 (0.79,46.93) | 1.91 (0.76,4.80) | 1.25 (0.23,6.74) | 2.96 (0.99,8.80) |
| 0.22 (0.03,1.73) | 1.10 (0.15,8.32) | 0.11 (0.00,3.63) | 0.19 (0.02,1.70) | 0.31 (0.04,2.79) | 0.16 (0.02,1.26) | Isavuconazole | 0.31 (0.04,2.58) | 0.21 (0.02,2.77) | 0.49 (0.09,2.71) |
| 0.69 (0.25,1.89) | 3.51 (1.39,8.90) | 0.35 (0.02,6.95) | 0.59 (0.17,2.08) | 1.00 (0.32,3.12) | 0.52 (0.21,1.31) | 3.19 (0.39,26.18) | Itraconazole | 0.65 (0.11,4.05) | 1.55 (0.46,5.18) |
| 1.05 (0.18,6.34) | 5.37 (0.85,33.84) | 0.53 (0.02,14.57) | 0.90 (0.12,6.73) | 1.53 (0.22,10.37) | 0.80 (0.15,4.31) | 4.87 (0.36,65.87) | 1.53 (0.25,9.47) | Posaconazole | 2.37 (0.33,16.70) |
| 0.45 (0.14,1.42) | 2.27 (0.78,6.60) | 0.23 (0.01,4.75) | 0.38 (0.10,1.53) | 0.65 (0.17,2.47) | 0.34 (0.11,1.01) | 2.06 (0.37,11.52) | 0.65 (0.19,2.16) | 0.42 (0.06,2.99) | Voriconazole |

**S2.30.** General disorders and administrative site conditions - empirical /definitive therapy subgroup

| _Placebo_ | _LAmB_ | _Anidulafungin_ | _Caspofungin_ | _Micafungin_ | _Fluconazole_ | _Isavuconazole_ | _Itraconazole_ | _Posaconazole_ | _Voriconazole_ |
| --- | --- | --- | --- | --- | --- | --- | --- | --- | --- |
| Placebo | 8.92 (2.09,38.04) | 0.87 (0.04,20.02) | 1.50 (0.31,7.20) | 3.68 (0.64,21.17) | 1.30 (0.38,4.51) | 4.55 (0.49,42.17) | 2.01 (0.28,14.22) | 1.01 (0.11,9.40) | 2.20 (0.56,8.61) |
| 0.11 (0.03,0.48) | LAmB | 0.10 (0.00,2.14) | 0.17 (0.06,0.49) | 0.41 (0.10,1.71) | 0.15 (0.05,0.45) | 0.51 (0.06,4.40) | 0.23 (0.04,1.28) | 0.11 (0.01,1.13) | 0.25 (0.07,0.88) |
| 1.15 (0.05,26.46) | 10.26 (0.47,225.43) | Anidulafungin | 1.72 (0.07,40.31) | 4.23 (0.17,102.61) | 1.50 (0.08,26.74) | 5.23 (0.14,190.69) | 2.31 (0.07,72.06) | 1.17 (0.03,40.02) | 2.53 (0.11,58.23) |
| 0.67 (0.14,3.22) | 5.97 (2.04,17.47) | 0.58 (0.02,13.63) | Caspofungin | 2.46 (0.53,11.35) | 0.87 (0.24,3.16) | 3.04 (0.30,31.19) | 1.34 (0.19,9.28) | 0.68 (0.06,7.34) | 1.47 (0.32,6.70) |
| 0.27 (0.05,1.57) | 2.43 (0.58,10.10) | 0.24 (0.01,5.75) | 0.41 (0.09,1.88) | Micafungin | 0.35 (0.09,1.40) | 1.24 (0.11,14.25) | 0.55 (0.06,4.66) | 0.28 (0.02,3.19) | 0.60 (0.11,3.27) |
| 0.77 (0.22,2.65) | 6.84 (2.24,20.91) | 0.67 (0.04,11.88) | 1.15 (0.32,4.15) | 2.82 (0.72,11.09) | Fluconazole | 3.49 (0.41,30.02) | 1.54 (0.23,10.10) | 0.78 (0.10,6.04) | 1.68 (0.49,5.84) |
| 0.22 (0.02,2.04) | 1.96 (0.23,16.92) | 0.19 (0.01,6.97) | 0.33 (0.03,3.37) | 0.81 (0.07,9.30) | 0.29 (0.03,2.47) | Isavuconazole | 0.44 (0.03,5.88) | 0.22 (0.01,4.13) | 0.48 (0.08,2.78) |
| 0.50 (0.07,3.52) | 4.44 (0.78,25.27) | 0.43 (0.01,13.51) | 0.74 (0.11,5.14) | 1.83 (0.21,15.60) | 0.65 (0.10,4.26) | 2.26 (0.17,30.14) | Itraconazole | 0.50 (0.03,7.70) | 1.09 (0.16,7.26) |
| 0.99 (0.11,9.14) | 8.80 (0.88,87.51) | 0.86 (0.02,29.44) | 1.47 (0.14,15.97) | 3.62 (0.31,41.91) | 1.29 (0.17,10.00) | 4.49 (0.24,83.19) | 1.98 (0.13,30.23) | Posaconazole | 2.17 (0.21,22.32) |
| 0.45 (0.12,1.78) | 4.06 (1.14,14.44) | 0.40 (0.02,9.12) | 0.68 (0.15,3.10) | 1.67 (0.31,9.15) | 0.59 (0.17,2.06) | 2.07 (0.36,11.92) | 0.91 (0.14,6.07) | 0.46 (0.04,4.75) | Voriconazole |

**S2.31.** General disorders and administrative site conditions - prophylaxis therapy subgroup

| _Placebo_ | _LAmB_ | _Micafungin_ | _Fluconazole_ | _Itraconazole_ | _Posaconazole_ | _Voriconazole_ |
| --- | --- | --- | --- | --- | --- | --- |
| Placebo | 0.01 (0.00,0.25) | 5.28 (0.59,47.43) | 99.41 (9.45,1046.12) | 0.98 (0.32,3.00) | 7.02 (0.07,677.32) | 0.57 (0.14,2.31) |
| 78.13 (4.07,1500.57) | LAmB | 0.01 (0.00,0.19) | 0.01 (0.00,0.08) | 0.01 (0.00,0.09) | 0.04 (0.00,2.07) | 4.47 (0.66,30.31) |
| 0.19 (0.02,1.70) | 90.02 (5.28,1536.23) | Micafungin | 0.88 (0.13,6.17) | 0.52 (0.06,4.27) | 3.52 (0.07,174.74) | 402.60 (13.14,12331.65) |
| 0.01 (0.00,0.11) | 101.74 (12.85,805.30) | 1.13 (0.16,7.87) | Fluconazole | 0.59 (0.10,3.51) | 3.98 (0.13,117.80) | 454.99 (27.17,7618.22) |
| 1.02 (0.33,3.14) | 173.64 (11.27,2675.60) | 1.93 (0.23,15.90) | 1.71 (0.29,10.21) | Itraconazole | 6.78 (0.15,313.15) | 776.54 (27.58,21866.31) |
| 0.14 (0.00,13.76) | 25.59 (0.48,1356.64) | 0.28 (0.01,14.12) | 0.25 (0.01,7.45) | 0.15 (0.00,6.80) | Posaconazole | 114.46 (1.39,9392.45) |
| 1.75 (0.43,7.04) | 0.22 (0.03,1.52) | 0.00 (0.00,0.08) | 0.00 (0.00,0.04) | 0.00 (0.00,0.04) | 0.01 (0.00,0.72) | Voriconazole |

**S2.32.** Gastrointestinal disorders

| _Placebo_ | _LAmB_ | _Anidulafungin_ | _Caspofungin_ | _Micafungin_ | _Fluconazole_ | _Isavuconazole_ | _Itraconazole_ | _Posaconazole_ | _Voriconazole_ |
| --- | --- | --- | --- | --- | --- | --- | --- | --- | --- |
| Placebo | 1.47 (0.77,2.84) | 0.60 (0.13,2.77) | 0.42 (0.18,0.95) | 0.77 (0.39,1.53) | 1.01 (0.59,1.71) | 1.01 (0.24,4.33) | 2.03 (1.10,3.77) | 1.03 (0.40,2.67) | 1.10 (0.51,2.38) |
| 0.68 (0.35,1.30) | LAmB | 0.41 (0.09,1.84) | 0.28 (0.15,0.53) | 0.52 (0.28,0.98) | 0.68 (0.41,1.13) | 0.69 (0.17,2.73) | 1.38 (0.85,2.24) | 0.70 (0.27,1.81) | 0.75 (0.40,1.39) |
| 1.66 (0.36,7.62) | 2.45 (0.54,11.02) | Anidulafungin | 0.69 (0.14,3.41) | 1.28 (0.28,5.96) | 1.67 (0.40,7.03) | 1.68 (0.24,12.01) | 3.37 (0.75,15.25) | 1.70 (0.33,8.92) | 1.83 (0.40,8.46) |
| 2.39 (1.05,5.42) | 3.52 (1.90,6.53) | 1.44 (0.29,7.07) | Caspofungin | 1.85 (0.84,4.04) | 2.41 (1.18,4.90) | 2.42 (0.55,10.75) | 4.85 (2.32,10.17) | 2.45 (0.84,7.20) | 2.63 (1.14,6.09) |
| 1.29 (0.66,2.55) | 1.91 (1.02,3.57) | 0.78 (0.17,3.62) | 0.54 (0.25,1.19) | Micafungin | 1.30 (0.74,2.30) | 1.31 (0.31,5.61) | 2.63 (1.39,4.96) | 1.33 (0.49,3.58) | 1.43 (0.66,3.08) |
| 0.99 (0.58,1.68) | 1.46 (0.88,2.43) | 0.60 (0.14,2.52) | 0.42 (0.20,0.85) | 0.77 (0.43,1.35) | Fluconazole | 1.01 (0.25,4.06) | 2.02 (1.21,3.36) | 1.02 (0.45,2.32) | 1.09 (0.57,2.10) |
| 0.99 (0.23,4.21) | 1.45 (0.37,5.78) | 0.59 (0.08,4.24) | 0.41 (0.09,1.83) | 0.76 (0.18,3.26) | 0.99 (0.25,4.01) | Isavuconazole | 2.00 (0.50,8.09) | 1.01 (0.20,5.08) | 1.09 (0.32,3.72) |
| 0.49 (0.26,0.91) | 0.73 (0.45,1.18) | 0.30 (0.07,1.34) | 0.21 (0.10,0.43) | 0.38 (0.20,0.72) | 0.50 (0.30,0.83) | 0.50 (0.12,2.02) | Itraconazole | 0.51 (0.19,1.31) | 0.54 (0.28,1.05) |
| 0.97 (0.38,2.53) | 1.44 (0.55,3.74) | 0.59 (0.11,3.08) | 0.41 (0.14,1.20) | 0.75 (0.28,2.03) | 0.98 (0.43,2.24) | 0.99 (0.20,4.96) | 1.98 (0.76,5.14) | Posaconazole | 1.07 (0.38,3.04) |
| 0.91 (0.42,1.96) | 1.34 (0.72,2.49) | 0.55 (0.12,2.53) | 0.38 (0.16,0.88) | 0.70 (0.32,1.52) | 0.91 (0.48,1.76) | 0.92 (0.27,3.15) | 1.84 (0.95,3.56) | 0.93 (0.33,2.64) | Voriconazole |

**S2.33.** Gastrointestinal disorders - empirical /definitive therapy subgroup

| _Placebo_ | _LAmB_ | _Anidulafungin_ | _Caspofungin_ | _Micafungin_ | _Fluconazole_ | _Isavuconazole_ | _Itraconazole_ | _Posaconazole_ | _Voriconazole_ |
| --- | --- | --- | --- | --- | --- | --- | --- | --- | --- |
| Placebo | 1.58 (0.60,4.19) | 0.69 (0.14,3.45) | 0.47 (0.16,1.33) | 1.14 (0.39,3.38) | 1.14 (0.51,2.55) | 1.20 (0.25,5.75) | 1.41 (0.33,5.97) | 1.17 (0.31,4.47) | 1.30 (0.45,3.71) |
| 0.63 (0.24,1.68) | LAmB | 0.44 (0.10,2.00) | 0.30 (0.16,0.54) | 0.72 (0.33,1.59) | 0.72 (0.38,1.38) | 0.76 (0.18,3.10) | 0.89 (0.27,2.94) | 0.74 (0.20,2.75) | 0.82 (0.37,1.81) |
| 1.45 (0.29,7.21) | 2.28 (0.50,10.42) | Anidulafungin | 0.67 (0.14,3.28) | 1.65 (0.34,8.04) | 1.65 (0.41,6.71) | 1.73 (0.25,11.95) | 2.03 (0.31,13.40) | 1.70 (0.28,10.38) | 1.88 (0.40,8.77) |
| 2.14 (0.75,6.11) | 3.38 (1.84,6.24) | 1.48 (0.31,7.20) | Caspofungin | 2.45 (1.02,5.88) | 2.45 (1.14,5.26) | 2.56 (0.57,11.45) | 3.01 (0.80,11.41) | 2.51 (0.64,9.85) | 2.78 (1.09,7.10) |
| 0.87 (0.30,2.58) | 1.38 (0.63,3.03) | 0.60 (0.12,2.94) | 0.41 (0.17,0.98) | Micafungin | 1.00 (0.47,2.15) | 1.05 (0.23,4.78) | 1.23 (0.31,4.94) | 1.03 (0.26,4.09) | 1.14 (0.43,3.01) |
| 0.87 (0.39,1.95) | 1.38 (0.73,2.62) | 0.60 (0.15,2.45) | 0.41 (0.19,0.88) | 1.00 (0.46,2.15) | Fluconazole | 1.04 (0.26,4.26) | 1.23 (0.34,4.46) | 1.02 (0.33,3.22) | 1.13 (0.52,2.48) |
| 0.84 (0.17,4.03) | 1.32 (0.32,5.42) | 0.58 (0.08,4.00) | 0.39 (0.09,1.75) | 0.96 (0.21,4.38) | 0.96 (0.23,3.91) | Isavuconazole | 1.18 (0.19,7.28) | 0.98 (0.16,5.98) | 1.09 (0.34,3.50) |
| 0.71 (0.17,3.02) | 1.12 (0.34,3.70) | 0.49 (0.07,3.24) | 0.33 (0.09,1.26) | 0.81 (0.20,3.26) | 0.81 (0.22,2.95) | 0.85 (0.14,5.26) | Itraconazole | 0.83 (0.15,4.70) | 0.92 (0.23,3.74) |
| 0.85 (0.22,3.25) | 1.35 (0.36,5.00) | 0.59 (0.10,3.61) | 0.40 (0.10,1.56) | 0.97 (0.24,3.89) | 0.98 (0.31,3.07) | 1.02 (0.17,6.21) | 1.20 (0.21,6.77) | Posaconazole | 1.11 (0.28,4.40) |
| 0.77 (0.27,2.20) | 1.22 (0.55,2.68) | 0.53 (0.11,2.49) | 0.36 (0.14,0.92) | 0.88 (0.33,2.33) | 0.88 (0.40,1.93) | 0.92 (0.29,2.96) | 1.08 (0.27,4.39) | 0.90 (0.23,3.58) | Voriconazole |

**S2.34.** Gastrointestinal disorders - prophylaxis therapy subgroup

| _Placebo_ | _LAmB_ | _Micafungin_ | _Fluconazole_ | _Itraconazole_ | _Posaconazole_ | _Voriconazole_ |
| --- | --- | --- | --- | --- | --- | --- |
| Placebo | 1.49 (0.49,4.51) | 0.56 (0.20,1.60) | 0.95 (0.41,2.19) | 2.01 (0.84,4.81) | 0.97 (0.20,4.67) | 1.02 (0.25,4.19) |
| 0.67 (0.22,2.04) | LAmB | 0.38 (0.11,1.29) | 0.64 (0.24,1.68) | 1.35 (0.63,2.88) | 0.65 (0.13,3.37) | 0.69 (0.21,2.22) |
| 1.79 (0.62,5.11) | 2.66 (0.77,9.12) | Micafungin | 1.69 (0.61,4.67) | 3.59 (1.29,10.01) | 1.74 (0.33,9.26) | 1.83 (0.41,8.27) |
| 1.06 (0.46,2.44) | 1.57 (0.59,4.15) | 0.59 (0.21,1.63) | Fluconazole | 2.12 (0.99,4.56) | 1.03 (0.26,3.98) | 1.08 (0.29,4.08) |
| 0.50 (0.21,1.19) | 0.74 (0.35,1.58) | 0.28 (0.10,0.78) | 0.47 (0.22,1.01) | Itraconazole | 0.48 (0.11,2.20) | 0.51 (0.17,1.57) |
| 1.03 (0.21,4.93) | 1.53 (0.30,7.88) | 0.58 (0.11,3.07) | 0.97 (0.25,3.78) | 2.07 (0.45,9.41) | Posaconazole | 1.05 (0.16,6.82) |
| 0.98 (0.24,3.99) | 1.45 (0.45,4.67) | 0.55 (0.12,2.47) | 0.92 (0.24,3.49) | 1.96 (0.64,6.05) | 0.95 (0.15,6.14) | Voriconazole |

**S2.35.** Cardiac disorders

| _Placebo_ | _LAmB_ | _Anidulafungin_ | _Caspofungin_ | _Micafungin_ | _Fluconazole_ | _Isavuconazole_ | _Itraconazole_ | _Posaconazole_ | _Voriconazole_ |
| --- | --- | --- | --- | --- | --- | --- | --- | --- | --- |
| Placebo | 5.02 (0.56,45.04) | 5.30 (0.11,250.56) | 0.97 (0.10,9.42) | 1.46 (0.36,5.86) | 1.89 (0.47,7.54) | 2.73 (0.13,56.07) | 4.19 (0.45,38.90) | 9.99 (0.49,205.09) | 2.86 (0.18,45.38) |
| 0.20 (0.02,1.79) | LAmB | 1.06 (0.02,63.90) | 0.19 (0.04,0.84) | 0.29 (0.04,1.91) | 0.38 (0.05,2.70) | 0.54 (0.06,5.18) | 0.83 (0.24,2.90) | 1.99 (0.11,36.20) | 0.57 (0.08,3.83) |
| 0.19 (0.00,8.93) | 0.95 (0.02,57.39) | Anidulafungin | 0.18 (0.00,11.87) | 0.27 (0.01,12.61) | 0.36 (0.01,13.00) | 0.51 (0.01,51.39) | 0.79 (0.01,48.93) | 1.89 (0.02,177.82) | 0.54 (0.01,45.75) |
| 1.03 (0.11,9.93) | 5.15 (1.19,22.35) | 5.44 (0.08,350.98) | Caspofungin | 1.49 (0.22,10.23) | 1.94 (0.24,15.83) | 2.80 (0.20,39.58) | 4.30 (0.70,26.44) | 10.25 (0.47,225.45) | 2.94 (0.28,30.87) |
| 0.69 (0.17,2.77) | 3.45 (0.52,22.77) | 3.64 (0.08,167.13) | 0.67 (0.10,4.58) | Micafungin | 1.30 (0.35,4.76) | 1.87 (0.11,30.94) | 2.88 (0.42,19.62) | 6.86 (0.38,124.07) | 1.97 (0.16,24.40) |
| 0.53 (0.13,2.12) | 2.66 (0.37,19.14) | 2.81 (0.08,102.70) | 0.52 (0.06,4.23) | 0.77 (0.21,2.84) | Fluconazole | 1.45 (0.08,25.54) | 2.22 (0.29,16.71) | 5.30 (0.33,85.32) | 1.52 (0.11,20.43) |
| 0.37 (0.02,7.54) | 1.84 (0.19,17.57) | 1.94 (0.02,193.94) | 0.36 (0.03,5.05) | 0.53 (0.03,8.81) | 0.69 (0.04,12.20) | Isavuconazole | 1.53 (0.17,14.22) | 3.66 (0.11,122.07) | 1.05 (0.29,3.74) |
| 0.24 (0.03,2.22) | 1.20 (0.34,4.18) | 1.27 (0.02,78.41) | 0.23 (0.04,1.43) | 0.35 (0.05,2.37) | 0.45 (0.06,3.39) | 0.65 (0.07,6.04) | Itraconazole | 2.39 (0.15,38.51) | 0.68 (0.11,4.35) |
| 0.10 (0.00,2.06) | 0.50 (0.03,9.15) | 0.53 (0.01,50.04) | 0.10 (0.00,2.15) | 0.15 (0.01,2.63) | 0.19 (0.01,3.04) | 0.27 (0.01,9.10) | 0.42 (0.03,6.76) | Posaconazole | 0.29 (0.01,7.65) |
| 0.35 (0.02,5.55) | 1.76 (0.26,11.79) | 1.85 (0.02,156.95) | 0.34 (0.03,3.58) | 0.51 (0.04,6.31) | 0.66 (0.05,8.87) | 0.95 (0.27,3.40) | 1.46 (0.23,9.30) | 3.49 (0.13,93.19) | Voriconazole |

**S2.36.** Cardiac disorders - empirical /definitive therapy subgroup

| _LAmB_ | _Caspofungin_ | _Micafungin_ | _Isavuconazole_ | _Itraconazole_ | _Voriconazole_ |
| --- | --- | --- | --- | --- | --- |
| LAmB | 0.23 (0.03,1.49) | 0.47 (0.03,7.65) | 0.73 (0.04,12.44) | 1.30 (0.16,10.41) | 0.82 (0.08,7.96) |
| 4.44 (0.67,29.27) | Caspofungin | 2.09 (0.16,27.29) | 3.23 (0.12,88.41) | 5.75 (0.44,74.73) | 3.64 (0.21,62.06) |
| 2.12 (0.13,34.43) | 0.48 (0.04,6.24) | Micafungin | 1.55 (0.04,62.34) | 2.75 (0.17,44.49) | 1.74 (0.07,46.17) |
| 1.37 (0.08,23.43) | 0.31 (0.01,8.47) | 0.65 (0.02,26.11) | Isavuconazole | 1.78 (0.10,31.48) | 1.13 (0.20,6.32) |
| 0.77 (0.10,6.19) | 0.17 (0.01,2.26) | 0.36 (0.02,5.88) | 0.56 (0.03,9.94) | Itraconazole | 0.63 (0.06,6.44) |
| 1.22 (0.13,11.81) | 0.27 (0.02,4.68) | 0.57 (0.02,15.23) | 0.89 (0.16,4.98) | 1.58 (0.16,16.07) | Voriconazole |

**S2.37.** Cardiac disorders - prophylaxis therapy subgroup

| _Placebo_ | _LAmB_ | _Micafungin_ | _Fluconazole_ | _Itraconazole_ | _Posaconazole_ |
| --- | --- | --- | --- | --- | --- |
| Placebo | 10.63 (0.70,160.86) | 1.51 (0.80,2.88) | 2.43 (0.83,7.11) | 5.70 (0.37,87.41) | 13.24 (0.87,202.70) |
| 0.09 (0.01,1.42) | LAmB | 0.14 (0.01,2.00) | 0.23 (0.02,2.78) | 0.54 (0.29,0.98) | 1.25 (0.10,15.82) |
| 0.66 (0.35,1.26) | 7.02 (0.50,98.43) | Micafungin | 1.61 (0.68,3.79) | 3.77 (0.27,53.51) | 8.75 (0.62,124.07) |
| 0.41 (0.14,1.20) | 4.37 (0.36,53.02) | 0.62 (0.26,1.47) | Fluconazole | 2.34 (0.19,28.84) | 5.44 (0.44,66.87) |
| 0.18 (0.01,2.69) | 1.86 (1.02,3.40) | 0.27 (0.02,3.77) | 0.43 (0.03,5.26) | Itraconazole | 2.32 (0.19,28.68) |
| 0.08 (0.00,1.16) | 0.80 (0.06,10.19) | 0.11 (0.01,1.62) | 0.18 (0.01,2.26) | 0.43 (0.03,5.31) | Posaconazole |

**Table S3.** According to SUCRA, the best (with the lowest side effect rate) and the worst (with the highest side effect rate) antifungal agents: prophylaxis therapy subgroup

| AEs | Best | Worst |
| --- | --- | --- |
| Withdrawal from study medication due to adverse events | Placebo | LAmB |
| Increase in total or direct bilirubin | - | - |
| Decrease in potassium | Voriconazole | LAmB |
| Increase in liver enzymes | Micafungin | Posaconazole |
| Nervous system disorders | Posaconazole | Voriconazole |
| Vascular disorders | - | - |
| Skin and subcutaneous tissue disorders | Placebo | Voriconazole |
| Respiratory, thoracic, and mediastinal disorders | - | - |
| Renal and urinary disorders | Fluconazole | Voriconazole |
| Hepatobiliary disorders | LAmB | Voriconazole |
| General disorders and administrative site conditions | Itraconazole | Voriconazole |
| Gastrointestinal disorders | Micafungin | Itraconazole |
| Cardiac disorders | Placebo | LAmB |

**Table S4.** According to SUCRA, the best (with the lowest side effect rate) and the worst (with the highest side effect rate) antifungal agents: empirical /definitive therapy subgroup

| AEs | Best | Worst |
| --- | --- | --- |
| Withdrawal from study medication due to adverse events | Caspofungin | LAmB |
| Increase in creatinine | Caspofungin | LAmB |
| Increase in total or direct bilirubin | Caspofungin | Micafungin |
| Decrease in potassium | Fluconazole | Placebo |
| Increase in liver enzymes | Anidulafungin | Posaconazole |
| Nervous system disorders | Itraconazole | Voriconazole |
| Vascular disorders | Placebo | LAmB |
| Skin and subcutaneous tissue disorders | Isavuconazole | Micafungin |
| Respiratory, thoracic, and mediastinal disorders | Isavuconazole | LAmB |
| Renal and urinary disorders | Isavuconazole | LAmB |
| Hepatobiliary disorders | Caspofungin | Voriconazole |
| General disorders and administrative site conditions | Placebo | Isavuconazole |
| Gastrointestinal disorders | Caspofungin | LAmB |
| Cardiac disorders | Caspofungin | Itraconazole |

**Table S5.** The inconsistency estimation results of each AE meta-analysis

| AEs | P value |
| --- | --- |
| Withdrawal from study medication due to adverse events | 0.9783 |
| Increase in total or direct bilirubin | 0.1854 |
| Decrease in potassium | 0.5668 |
| Increase in liver enzymes | 0.4645 |
| Nervous system disorders | 0.7283 |
| Vascular disorders | 0.8899 |
| Skin and subcutaneous tissue disorders | 0.1836 |
| Respiratory, thoracic, and mediastinal disorders | 0.4369 |
| Renal and urinary disorders | 0.8931 |
| Hepatobiliary disorders | 0.8543 |
| General disorders and administrative site conditions | 0.9969 |
| Gastrointestinal disorders | 0.1797 |
| Cardiac disorders | 0.5957 |

A P value < 0.05 indicates a significant inconsistency

**Table S6.** The inconsistency estimation results of each AE meta-analysis: empirical /definitive therapy subgroup

| AEs | P value |
| --- | --- |
| Withdrawal from study medication due to adverse events | 0.9908 |
| Increase in total or direct bilirubin | 0.2038 |
| Decrease in potassium | 0.7509 |
| Increase in liver enzymes | 0.1912 |
| Nervous system disorders | 0.6312 |
| Vascular disorders | 0.8899 |
| Skin and subcutaneous tissue disorders | 0.0447 |
| Respiratory, thoracic, and mediastinal disorders | 0.6583 |
| Renal and urinary disorders | 1 |
| Hepatobiliary disorders | 0.6067 |
| General disorders and administrative site conditions | 0.9934 |
| Gastrointestinal disorders | 0.4864 |
| Cardiac disorders | 0.2578 |

A P value < 0.05 indicates a significant inconsistency

**Table S7.** The inconsistency estimation results of each AE meta-analysis: prophylaxis therapy subgroup

| AEs | P value |
| --- | --- |
| Withdrawal from study medication due to adverse events | 0.5289 |
| Increase in total or direct bilirubin | - |
| Decrease in potassium | 0.021 |
| Increase in liver enzymes | 0.4225 |
| Nervous system disorders | 0.9956 |
| Vascular disorders | - |
| Skin and subcutaneous tissue disorders | 0.0946 |
| Respiratory, thoracic, and mediastinal disorders |  |
| Renal and urinary disorders | 0.5958 |
| Hepatobiliary disorders | 0.6201 |
| General disorders and administrative site conditions | 0.0483 |
| Gastrointestinal disorders | 0.376 |
| Cardiac disorders | 0.7151 |

A P value < 0.05 indicates a significant inconsistency

**Figure S1** Risk of bias graph


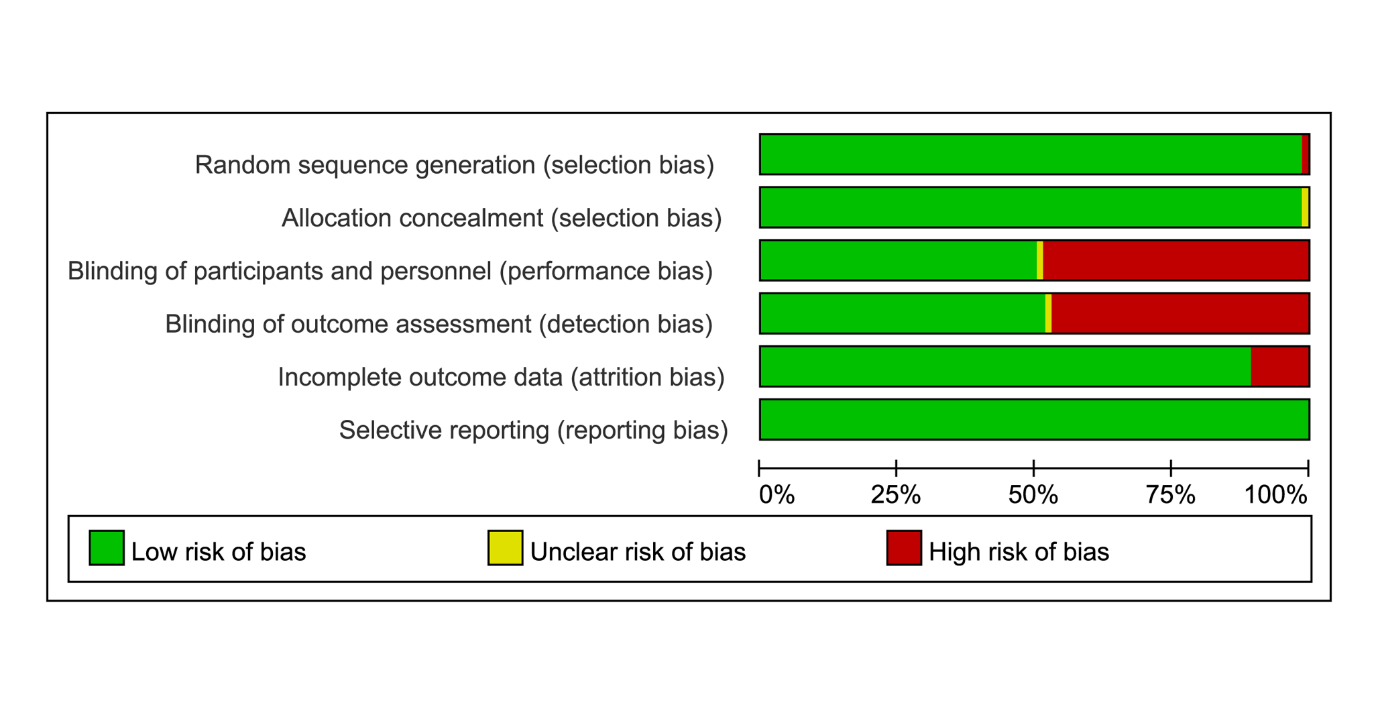


**Figure S2** Risk of bias summary graph


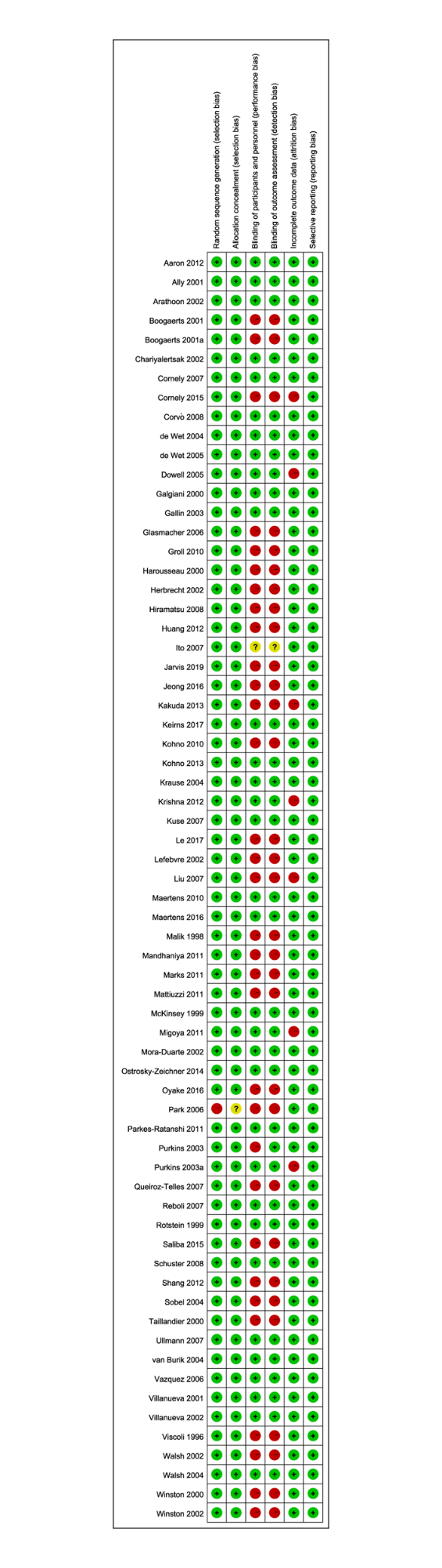


**Figure S3** Network plot for Cardiac disorders analysis


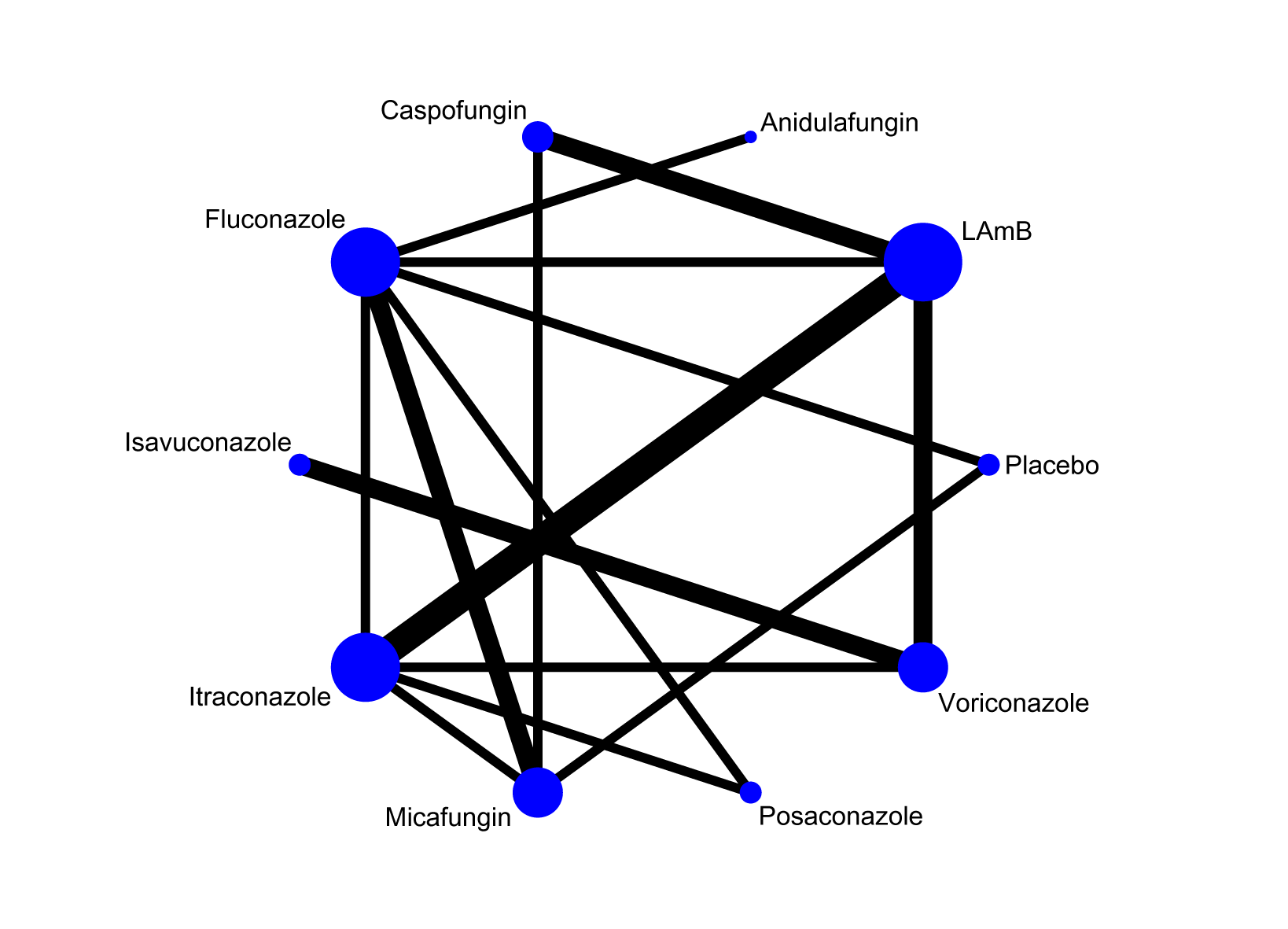


**Figure S4** Network plot for Gastrointestinal disorders analysis


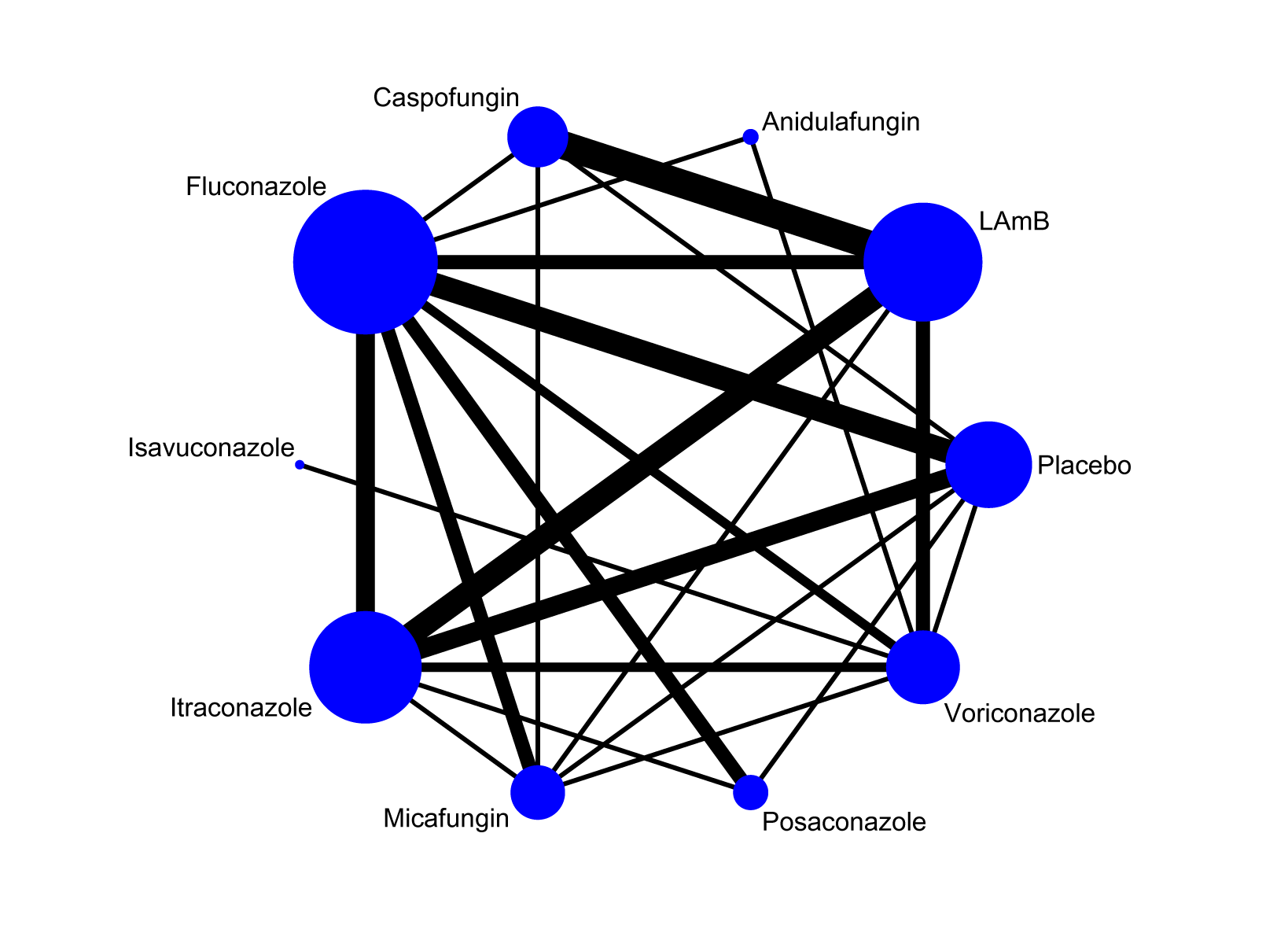


**Figure S5** Network plot for General disorders and administrative site conditions analysis


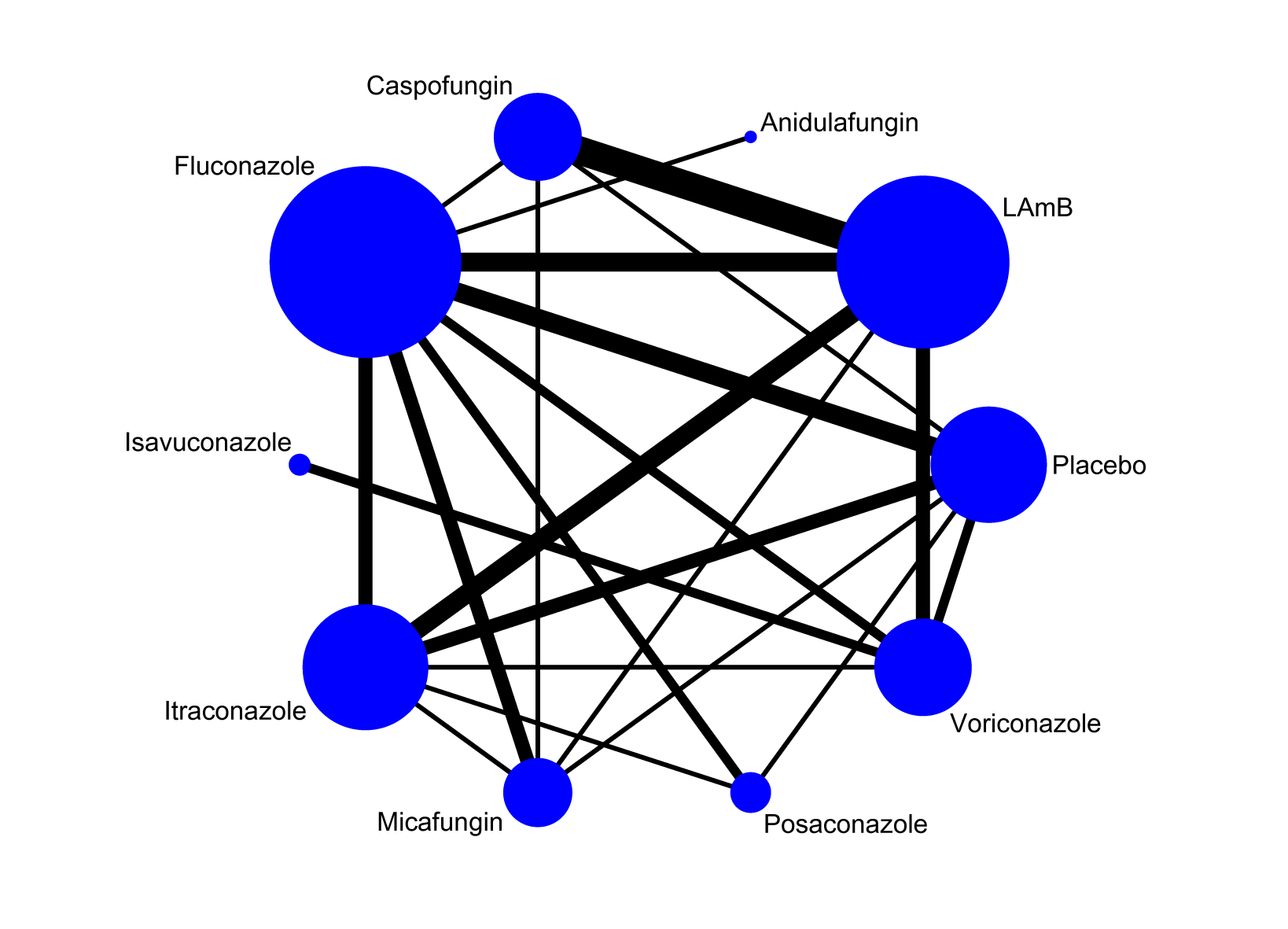


**Figure S6** Network plot for Hepatobiliary disorders analysis


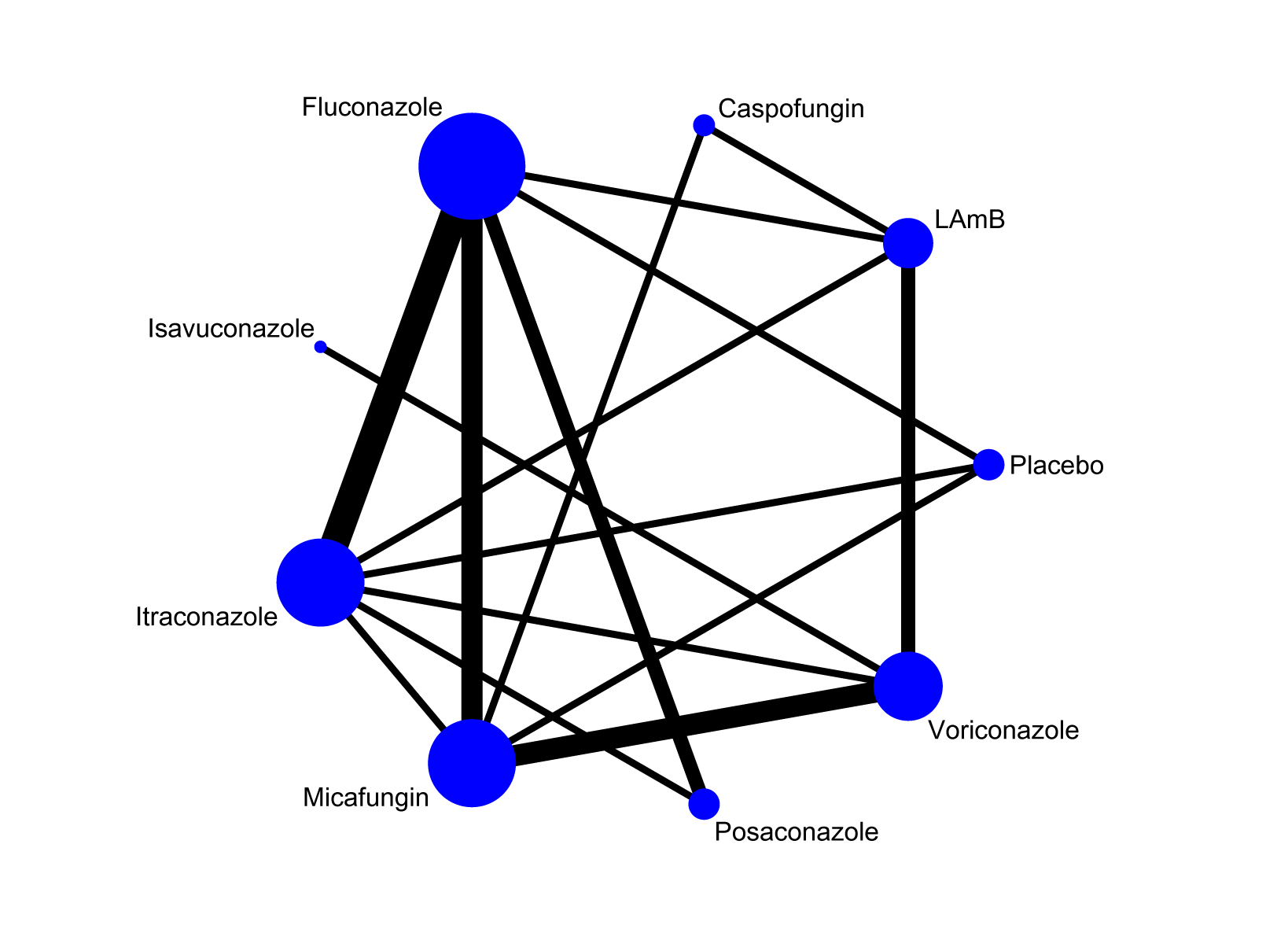


**Figure S7** Network plot for Renal and urinary disorders analysis


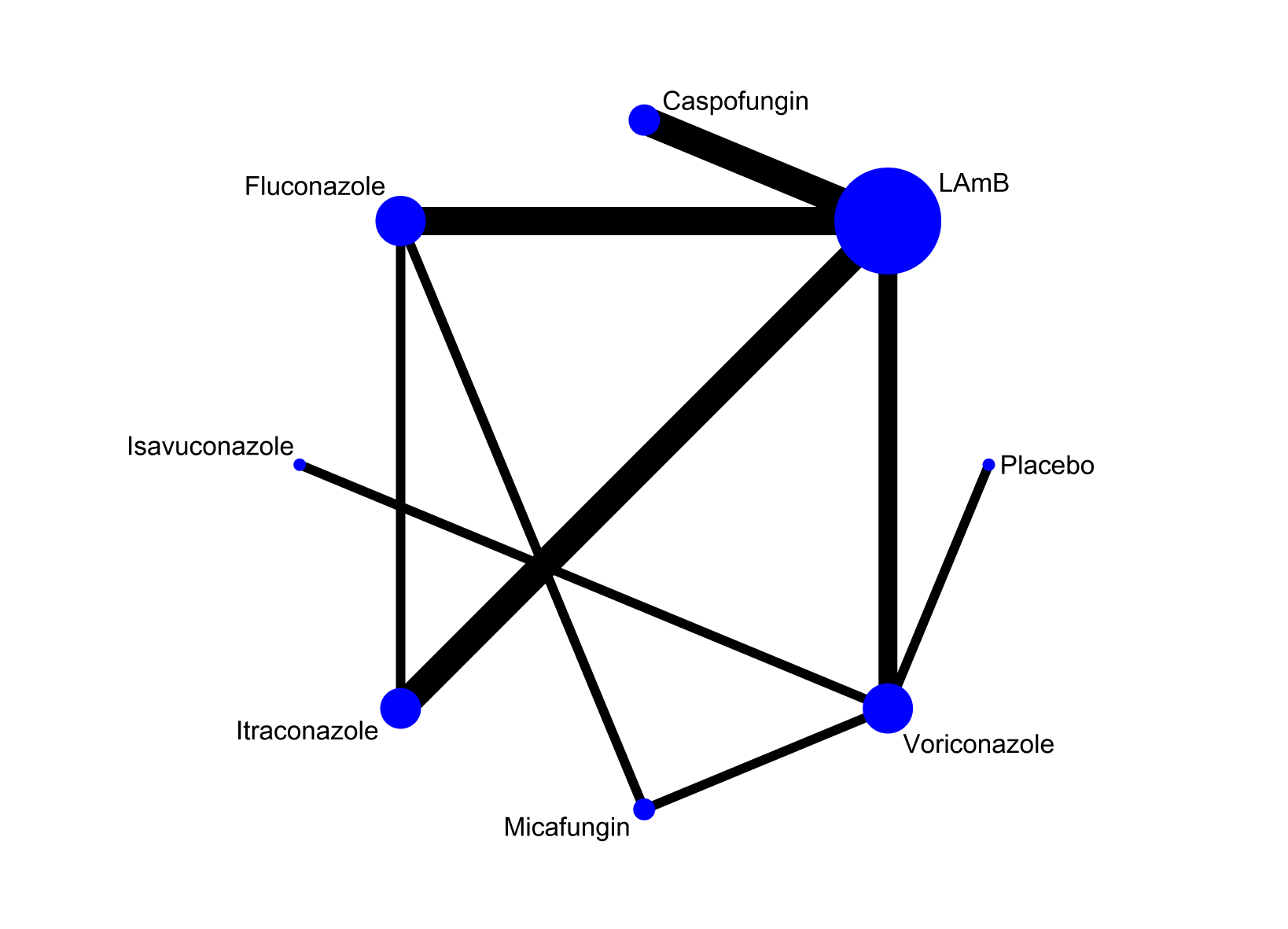


**Figure S8** Network plot for Respiratory, thoracic, and mediastinal disorders analysis


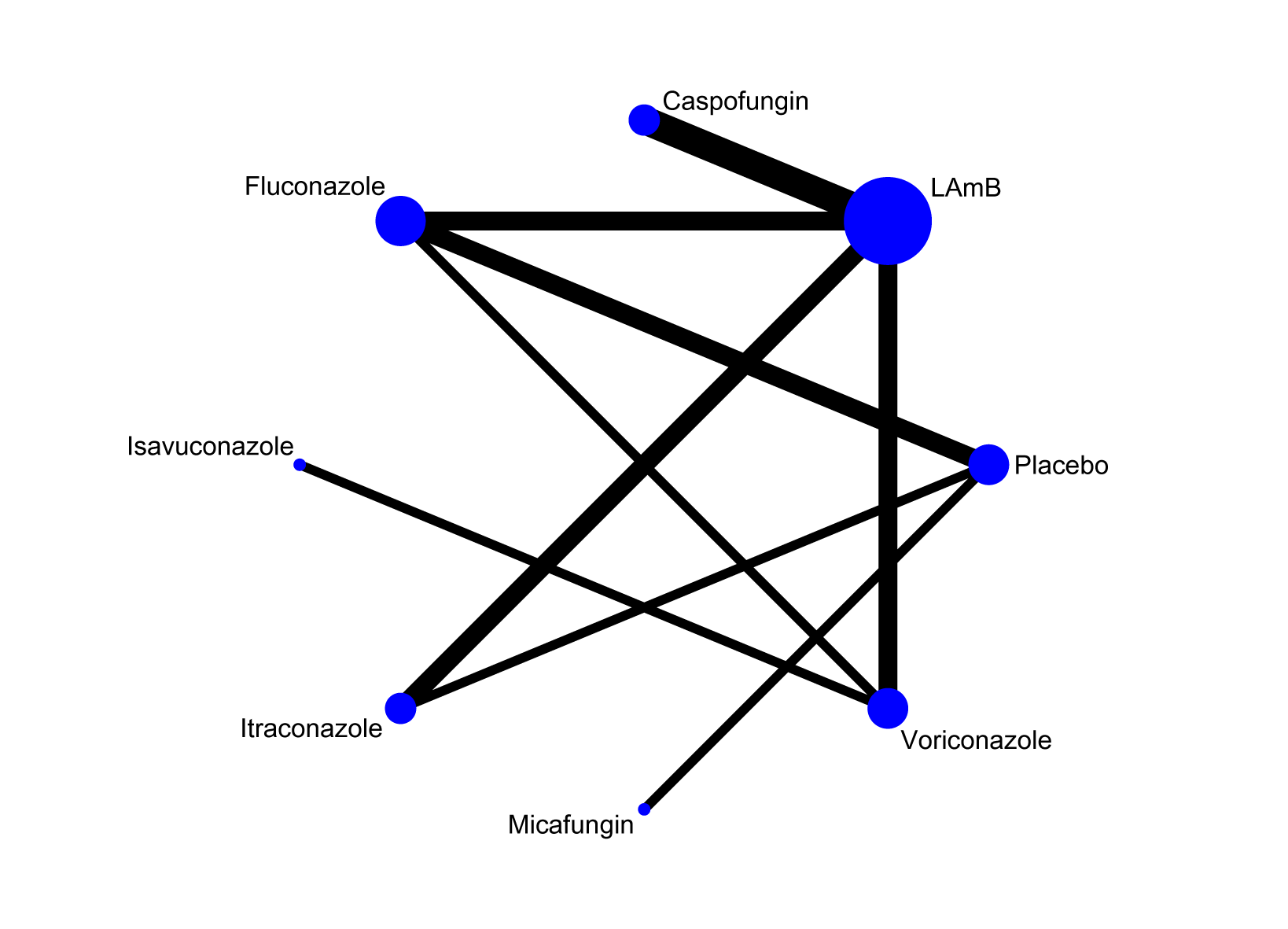


**Figure S9** Network plot for Skin and subcutaneous tissue disorders analysis


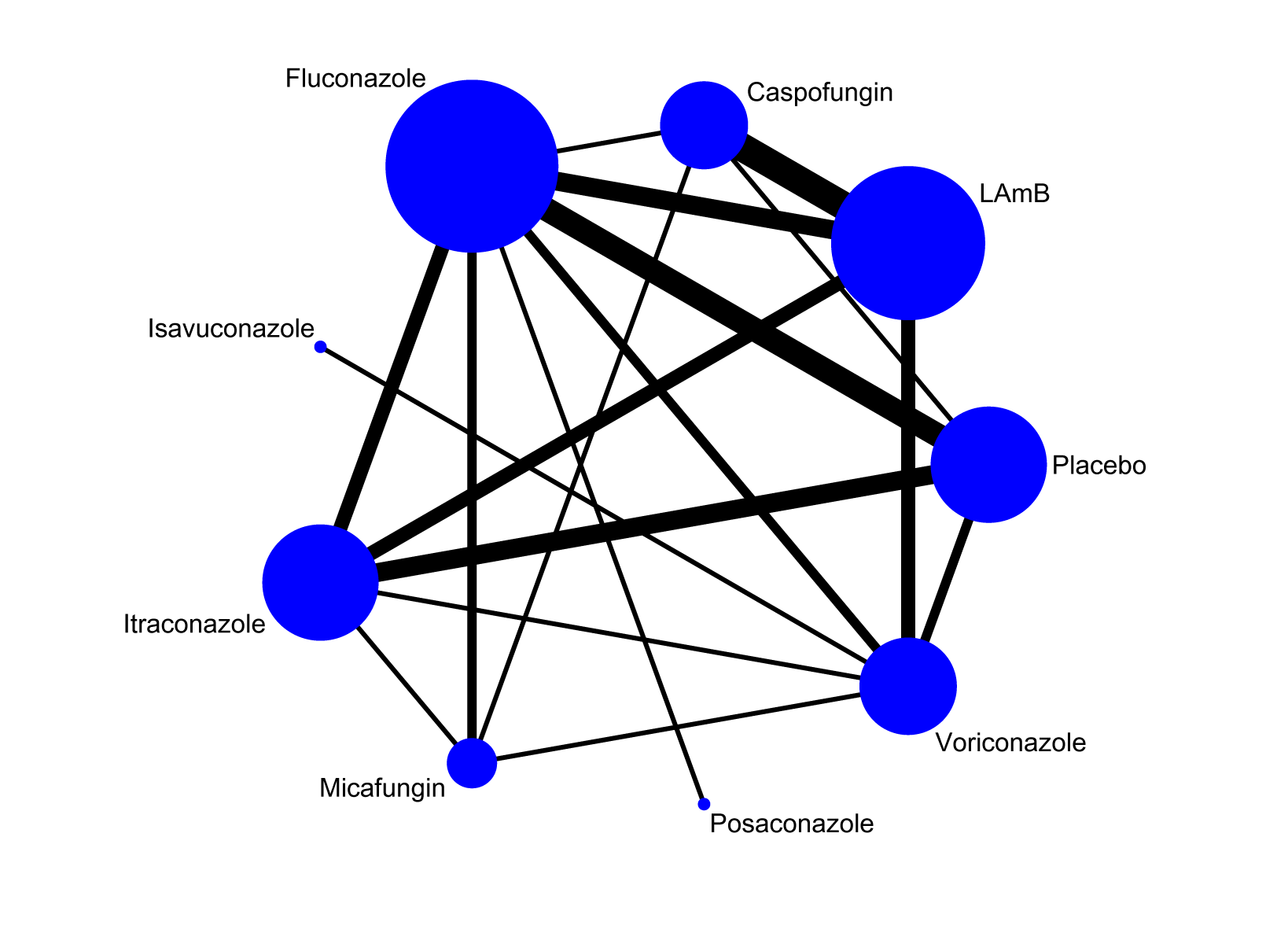


**Figure S10** Network plot for Vascular disorders analysis


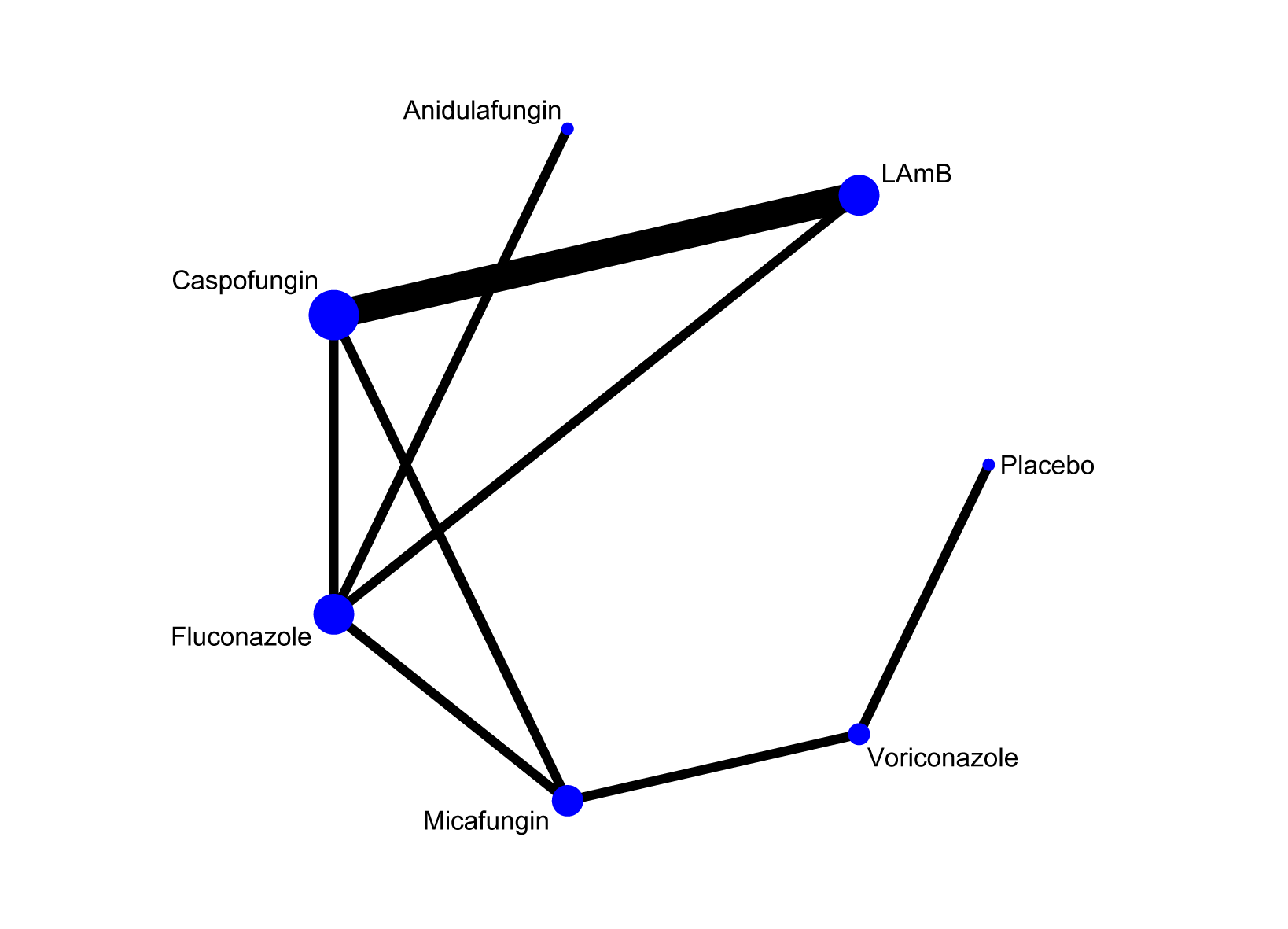


**Figure S11** Network plot for Nervous system disorders analysis


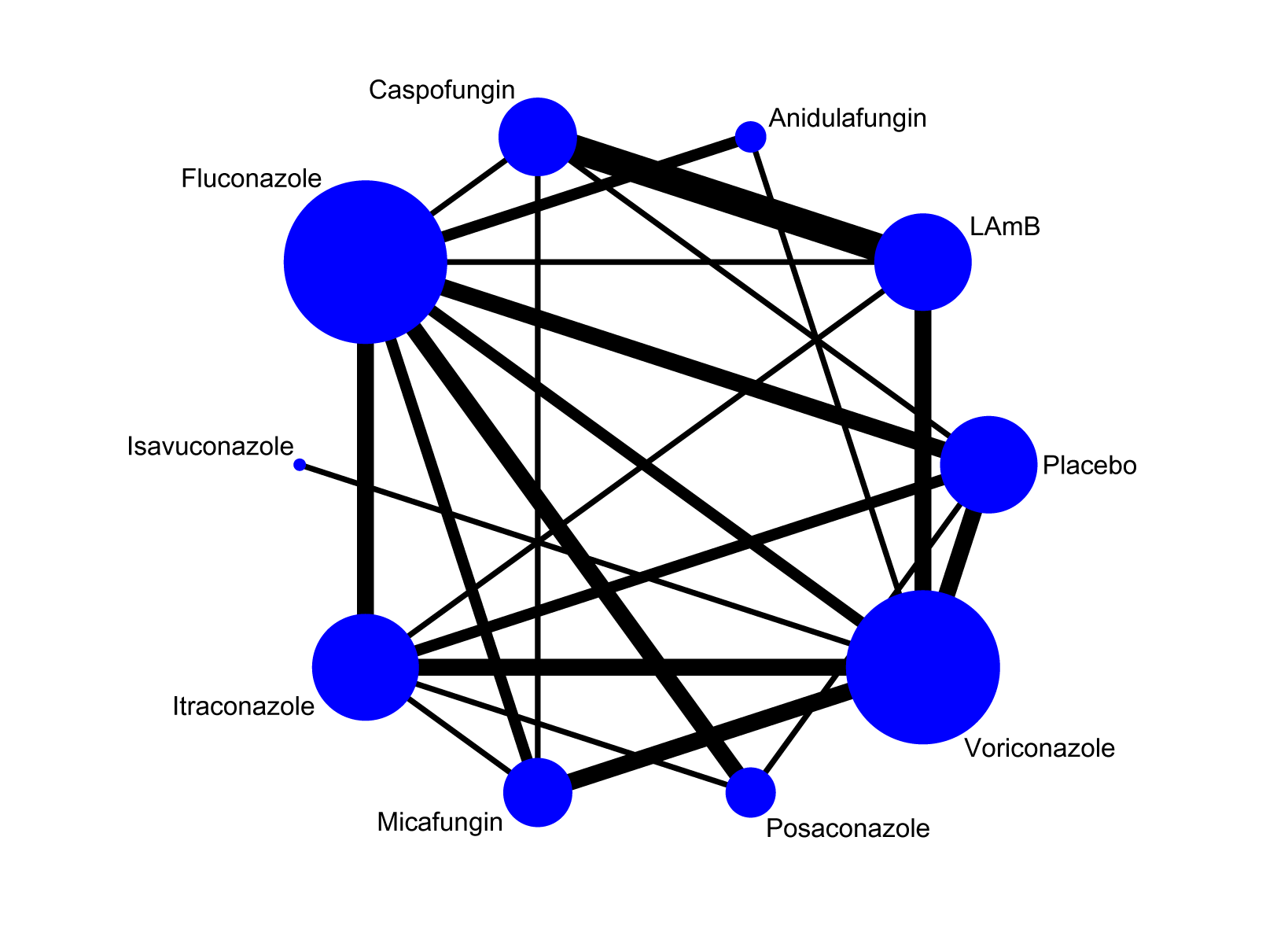


**Figure S12** Network plot for Increase in liver enzymes analysis


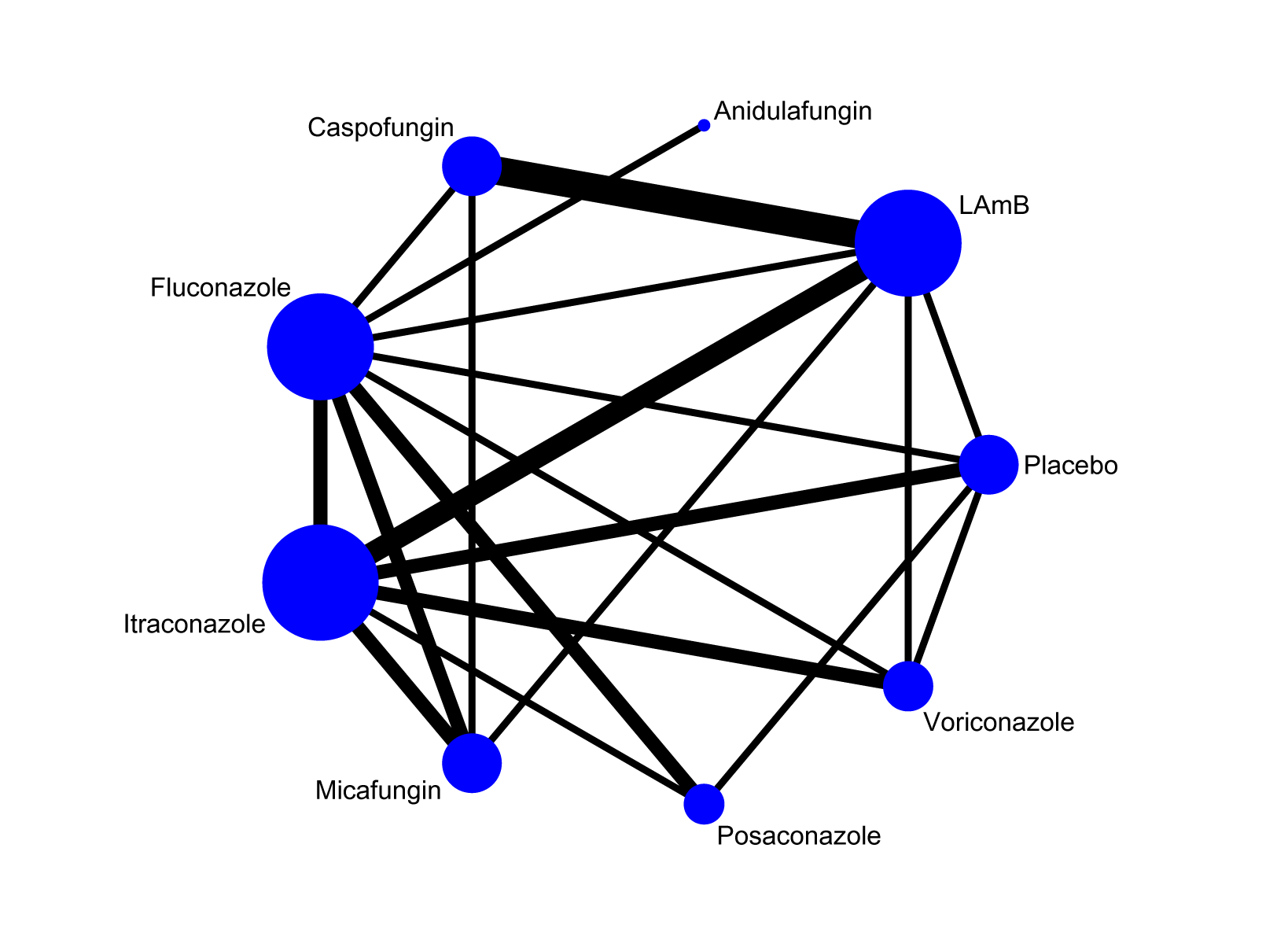


**Figure S13** Network plot for Decrease in potassium analysis


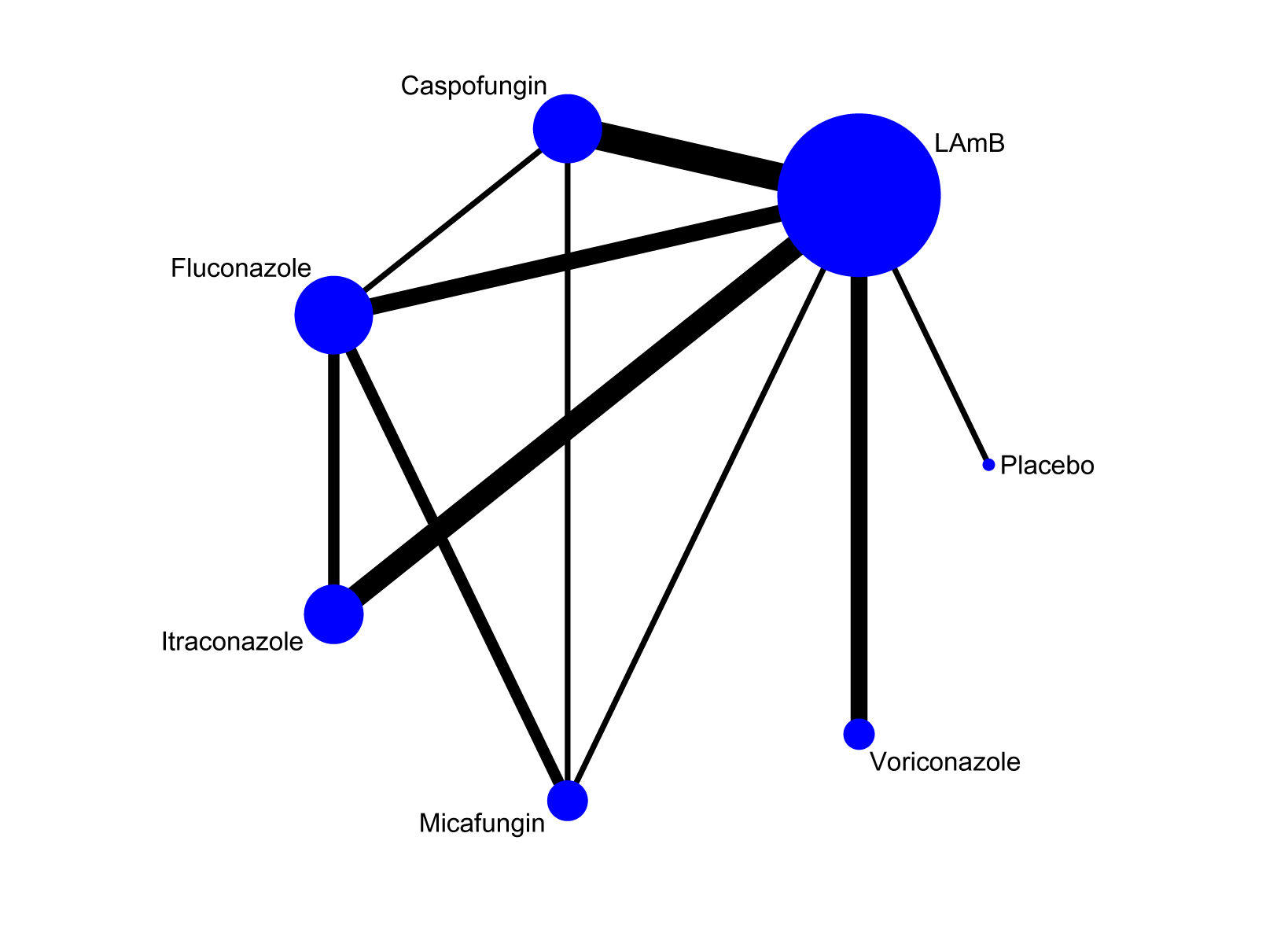


**Figure S14** Network plot for Increase in total or direct bilirubin analysis


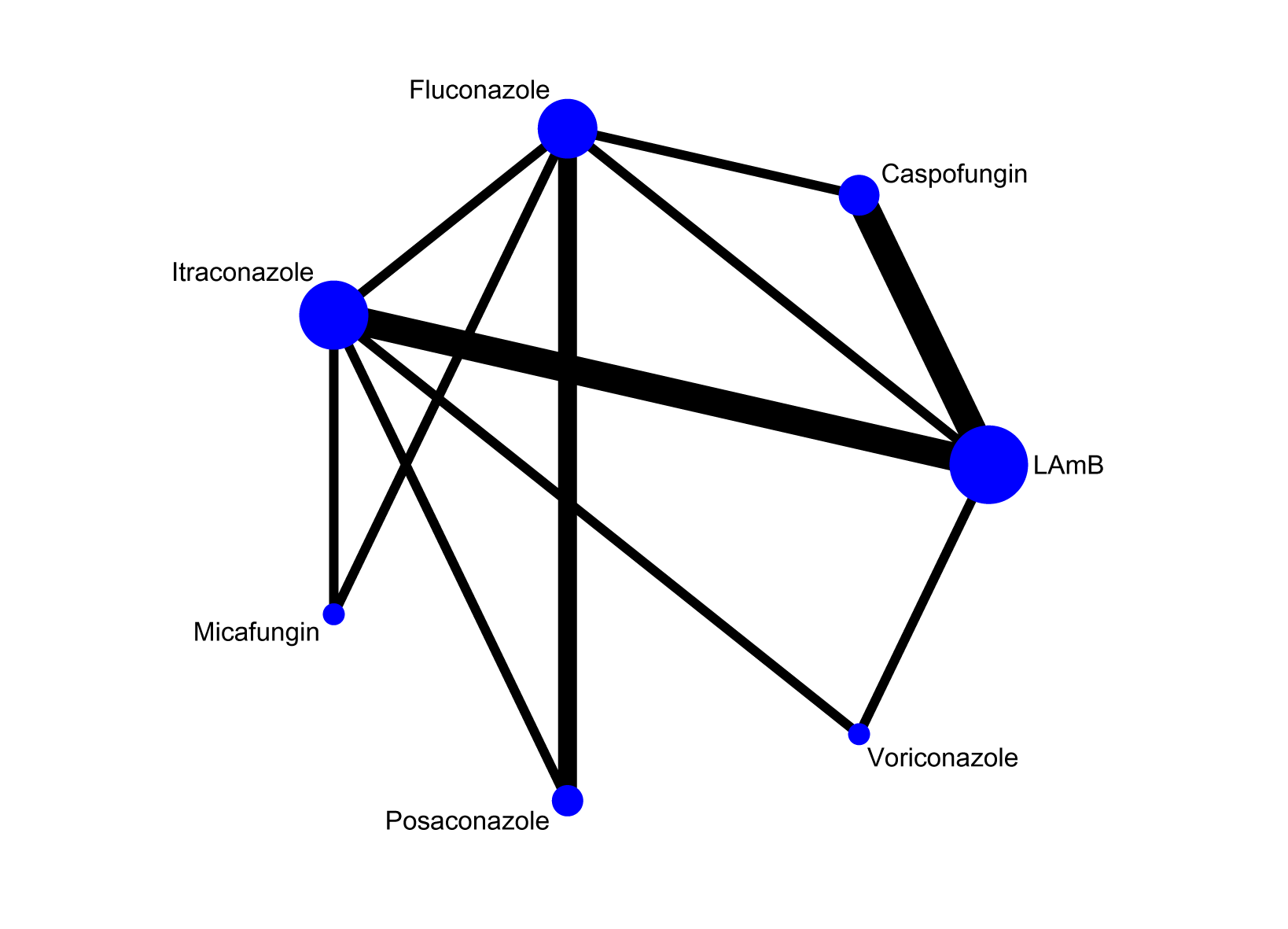


**Figure S15** Network plot for Increase in creatinine analysis


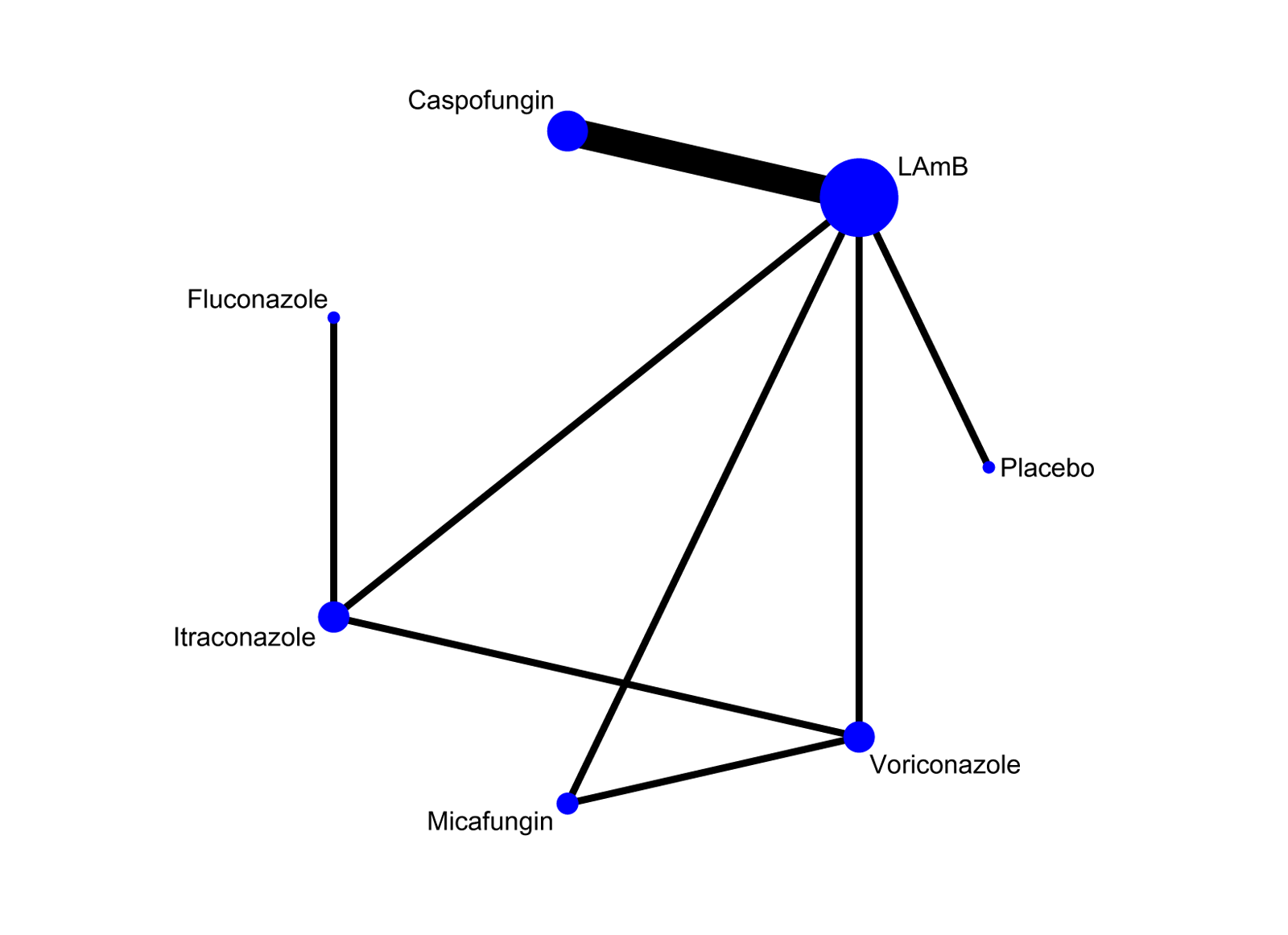


**Figure S16** Funnel plot for Cardiac disorders analysis


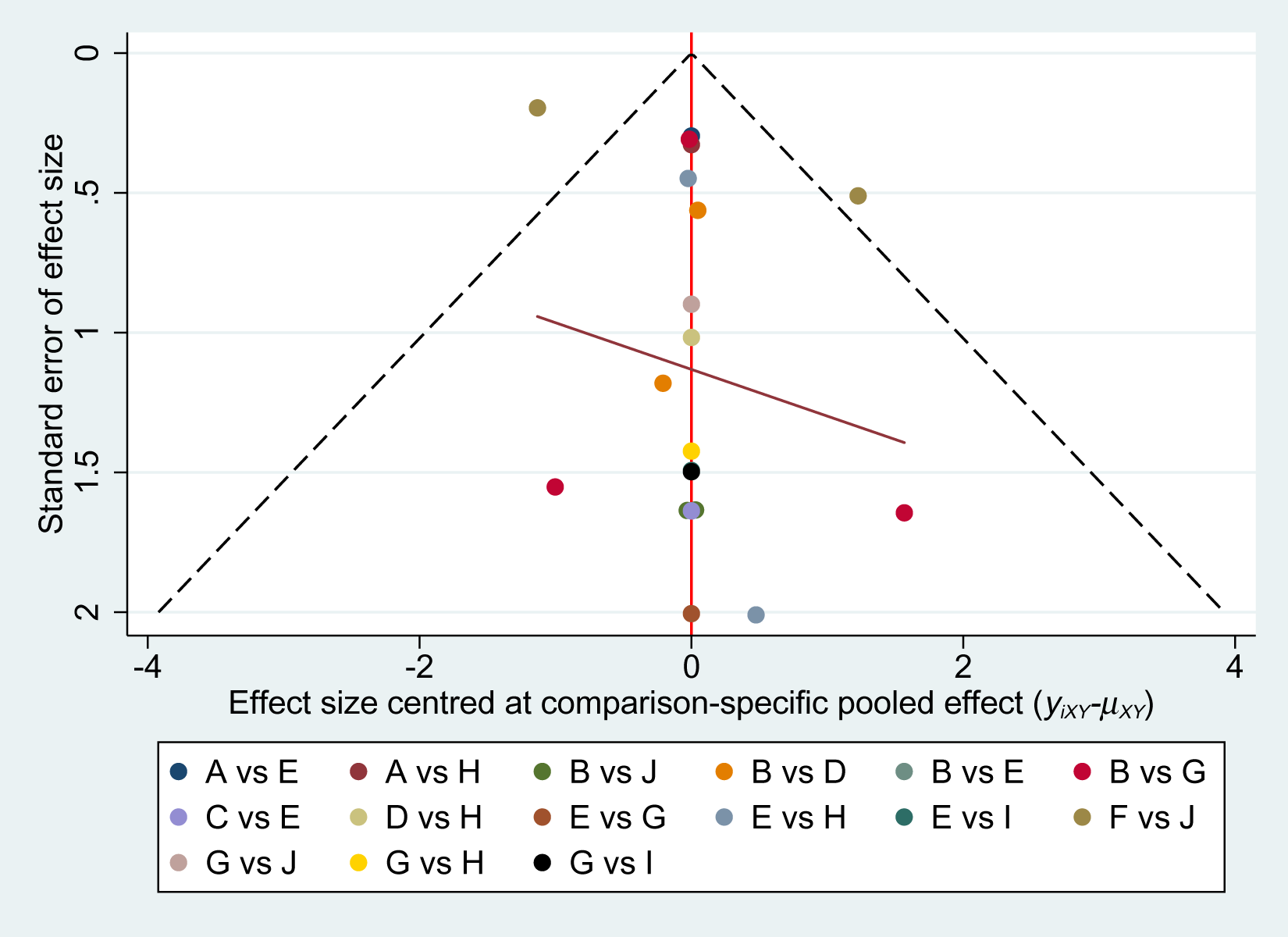


**Figure S17** Funnel plot for Gastrointestinal disorders analysis

**
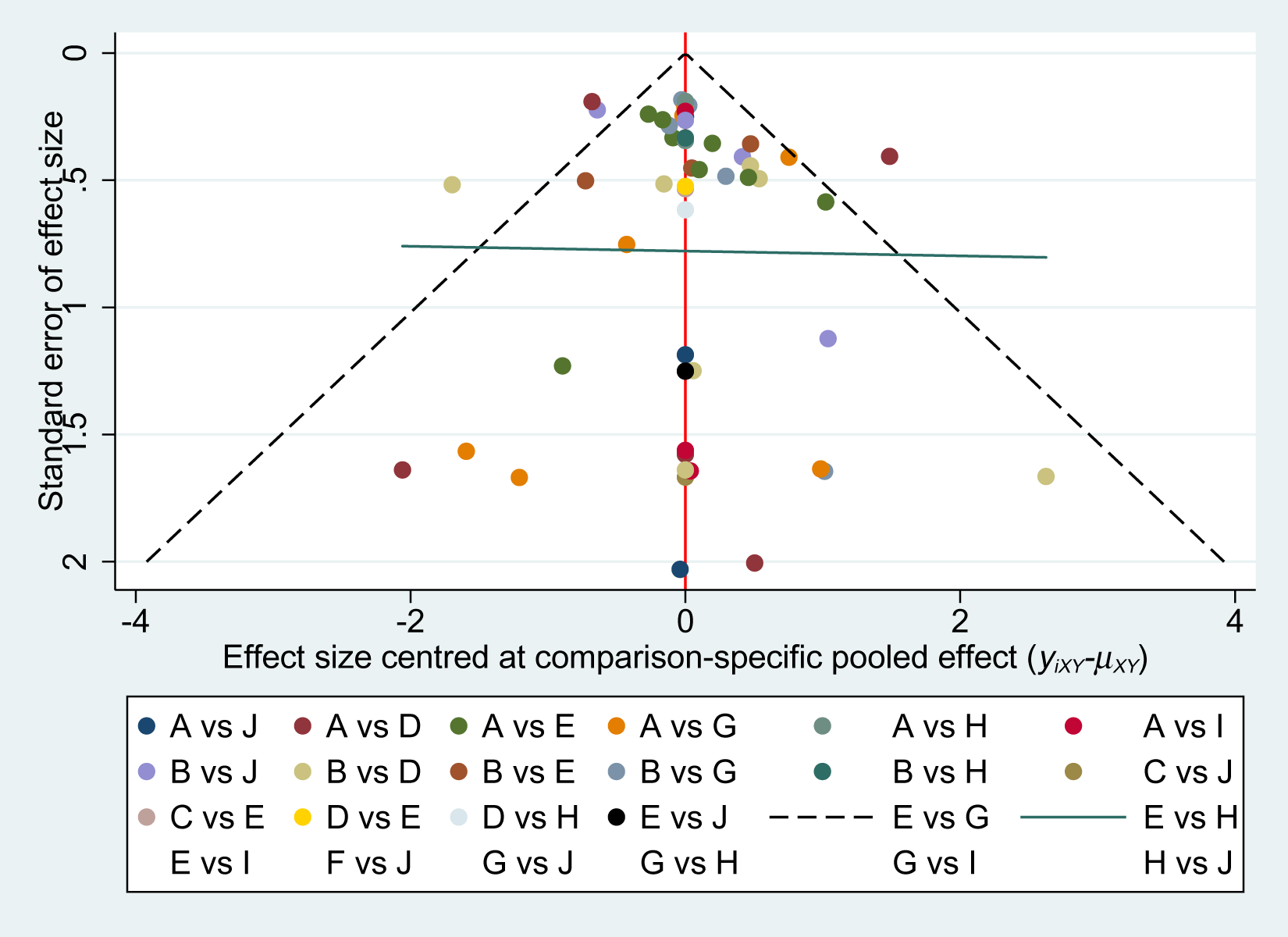
**

**Figure S18** Funnel plot for General disorders and administrative site conditions analysis


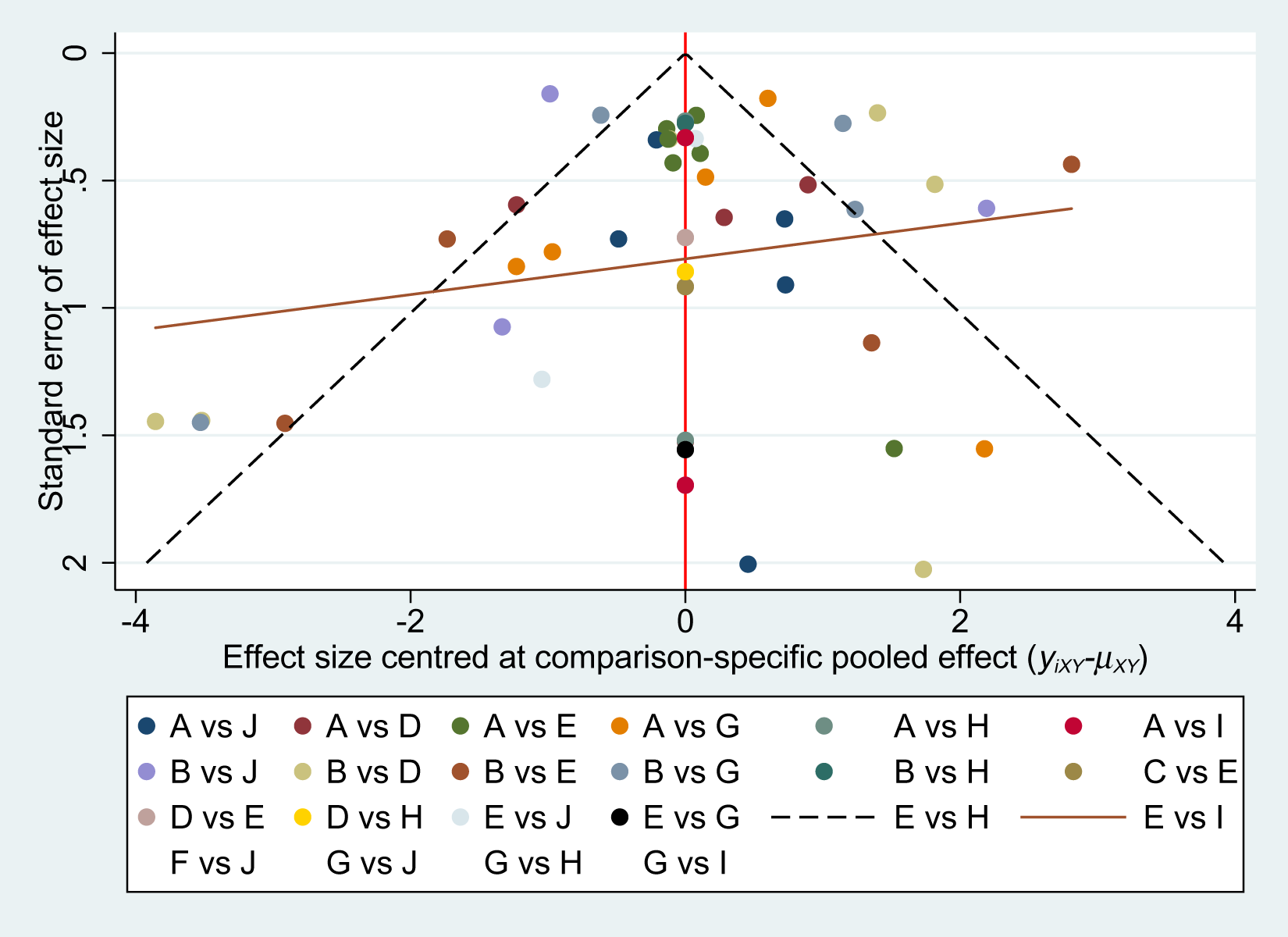


**Figure S19** Funnel plot for Hepatobiliary disorders analysis


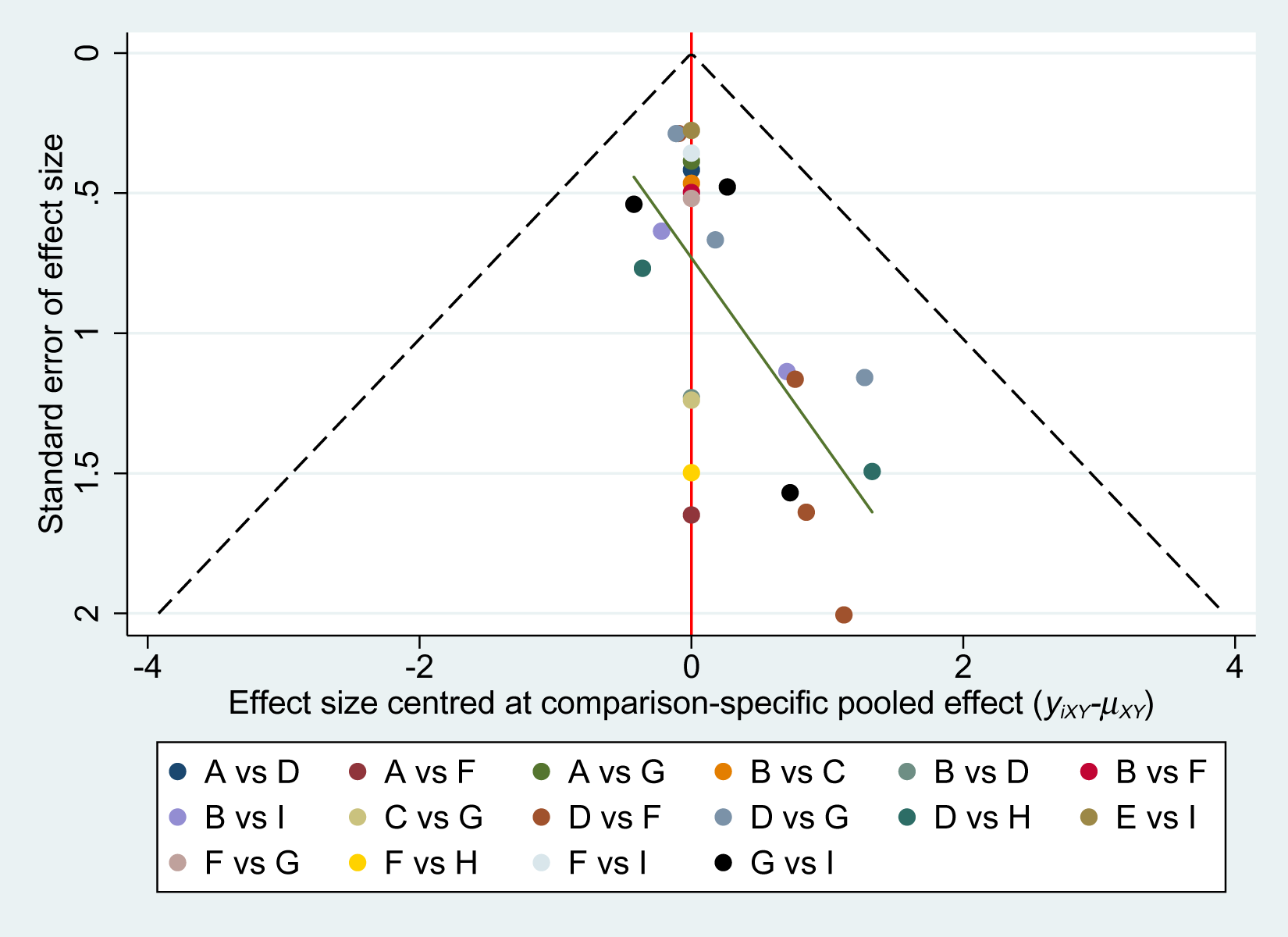


**Figure S20** Funnel plot for Renal and urinary disorders analysis


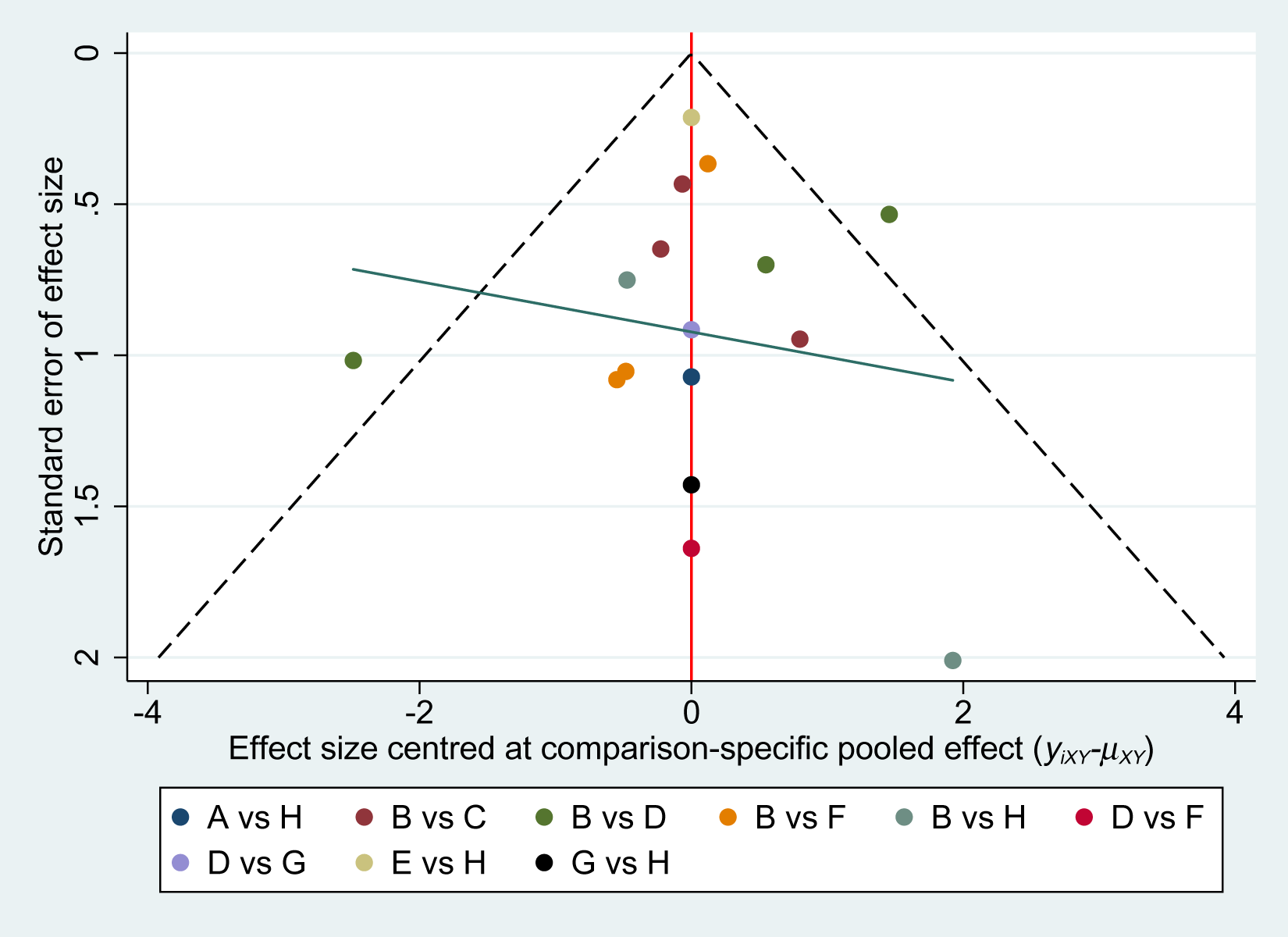


**Figure S21** Funnel plot for Respiratory, thoracic, and mediastinal disorders analysis


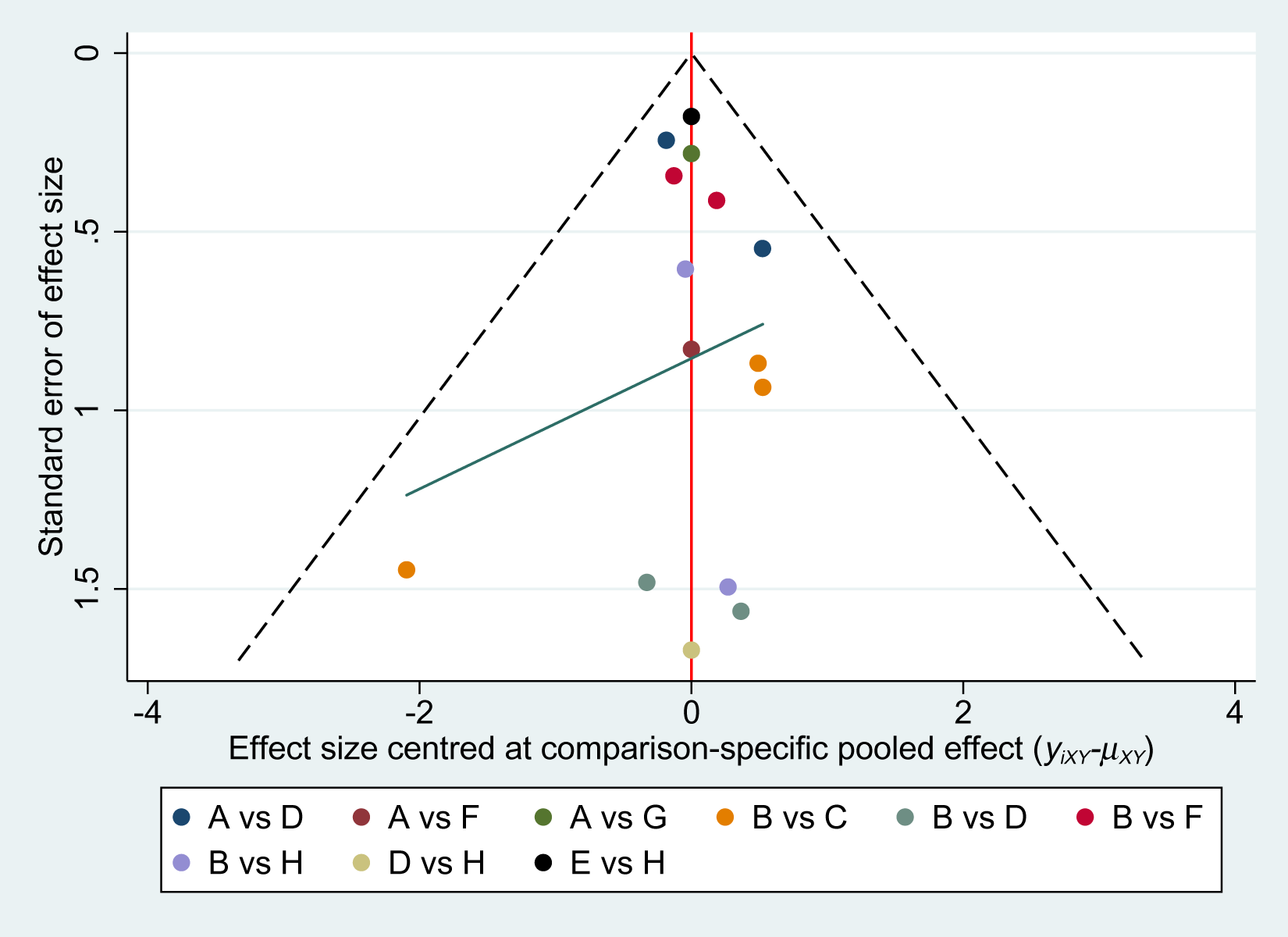


**Figure S22** Funnel plot for Skin and subcutaneous tissue disorders analysis


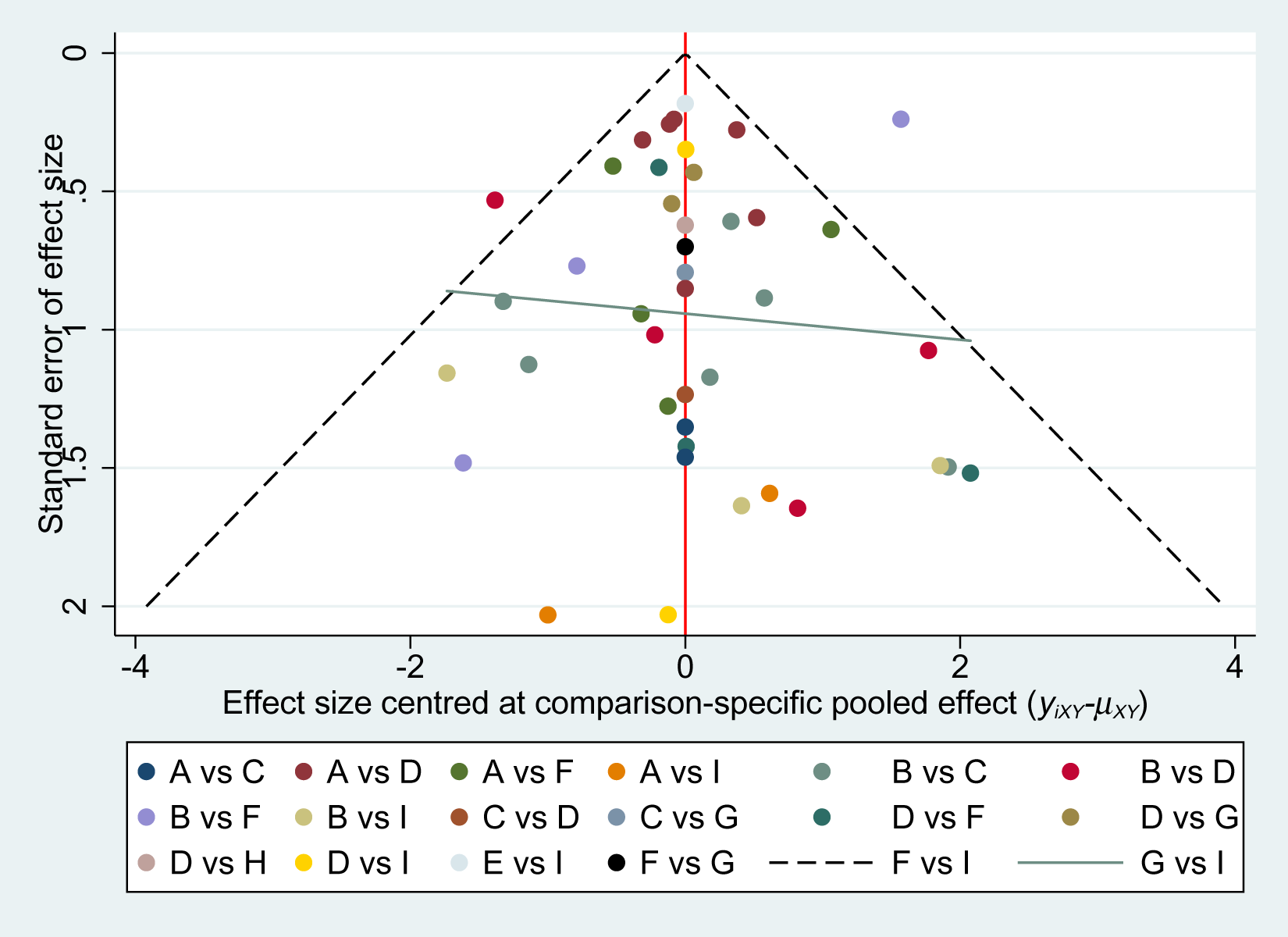


**Figure S23** Funnel plot for Vascular disorders analysis


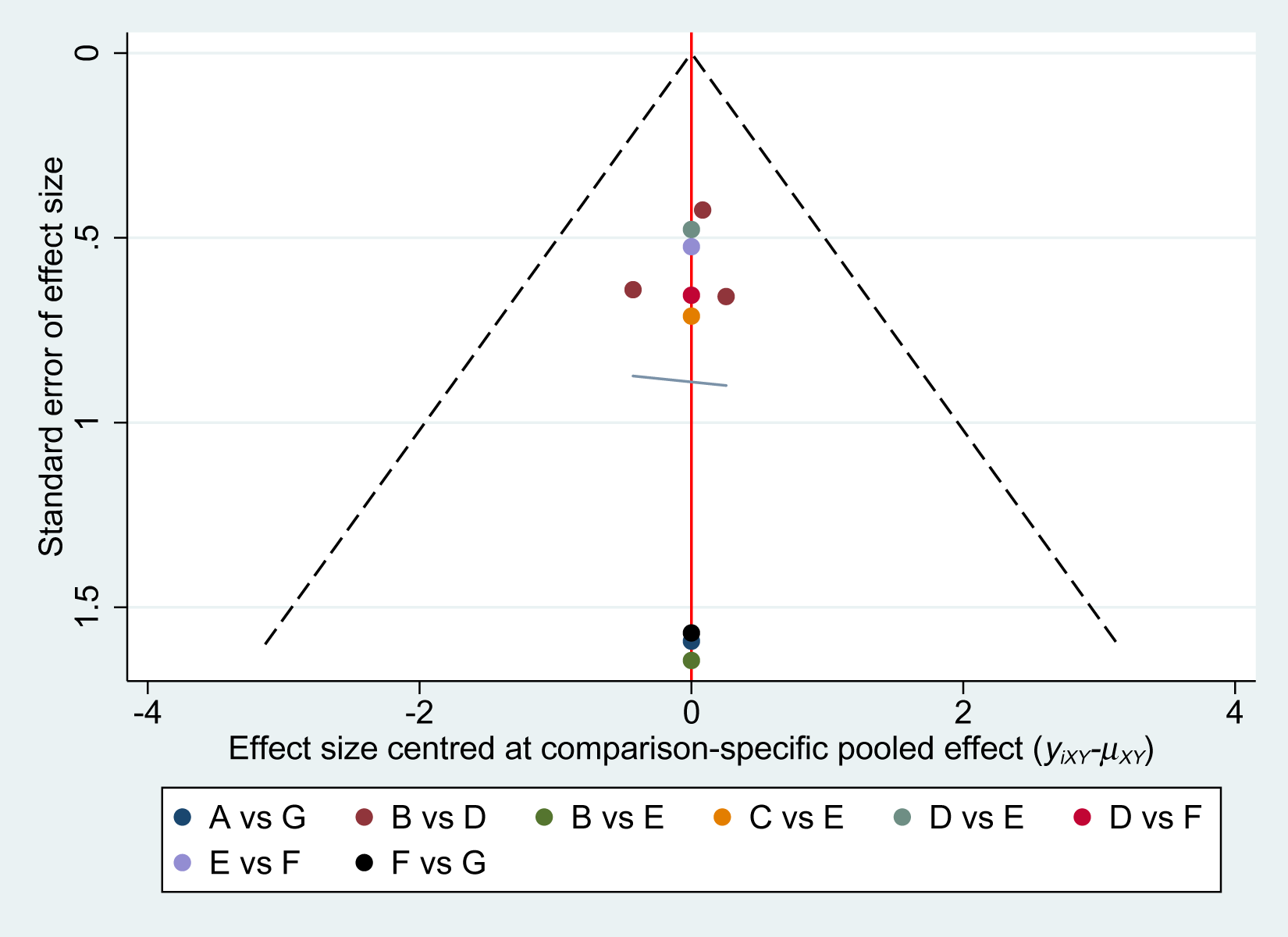


**Figure S24** Funnel plot for Nervous system disorders analysis


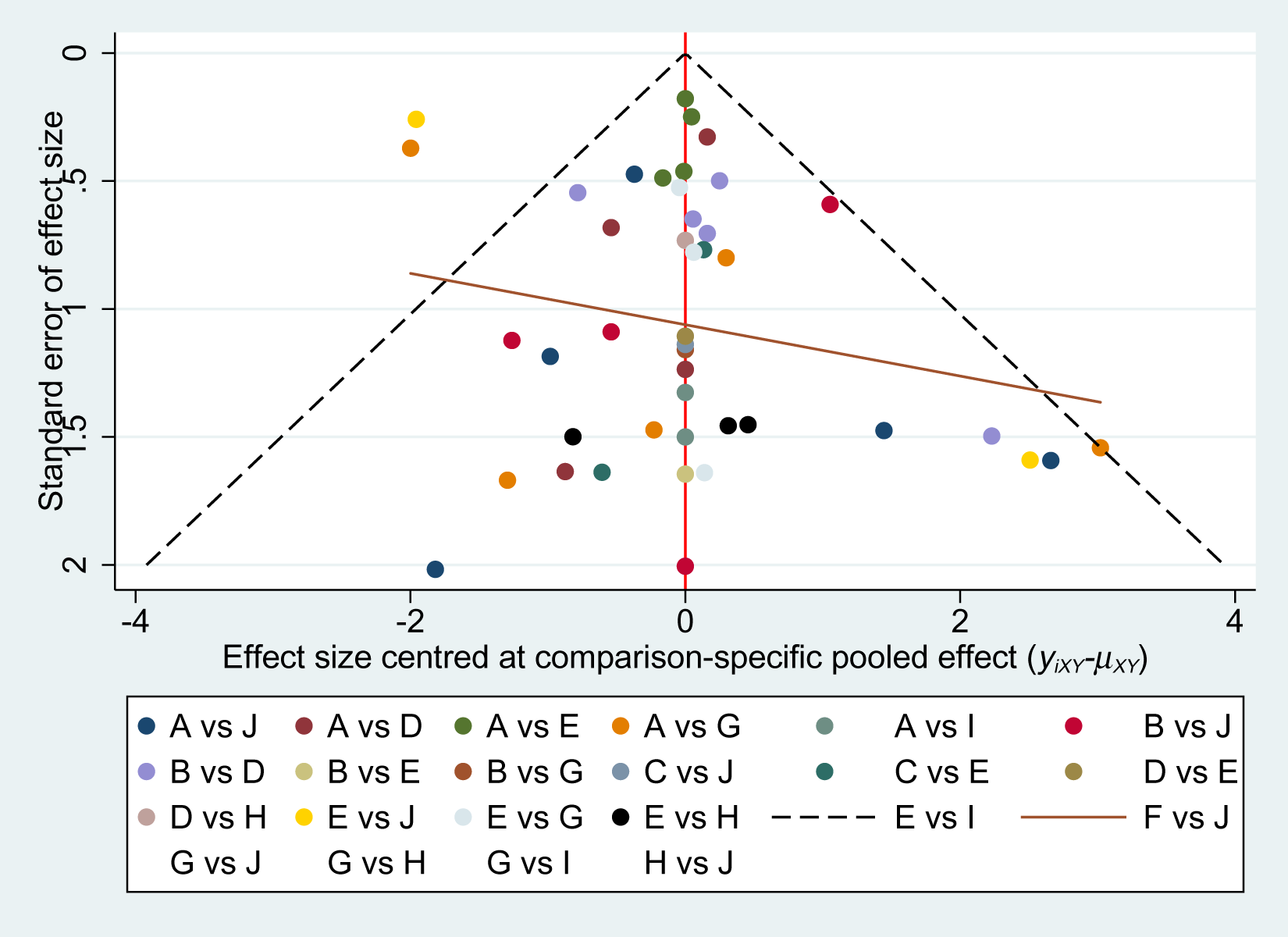


**Figure S25** Funnel plot for Increase in liver enzymes analysis


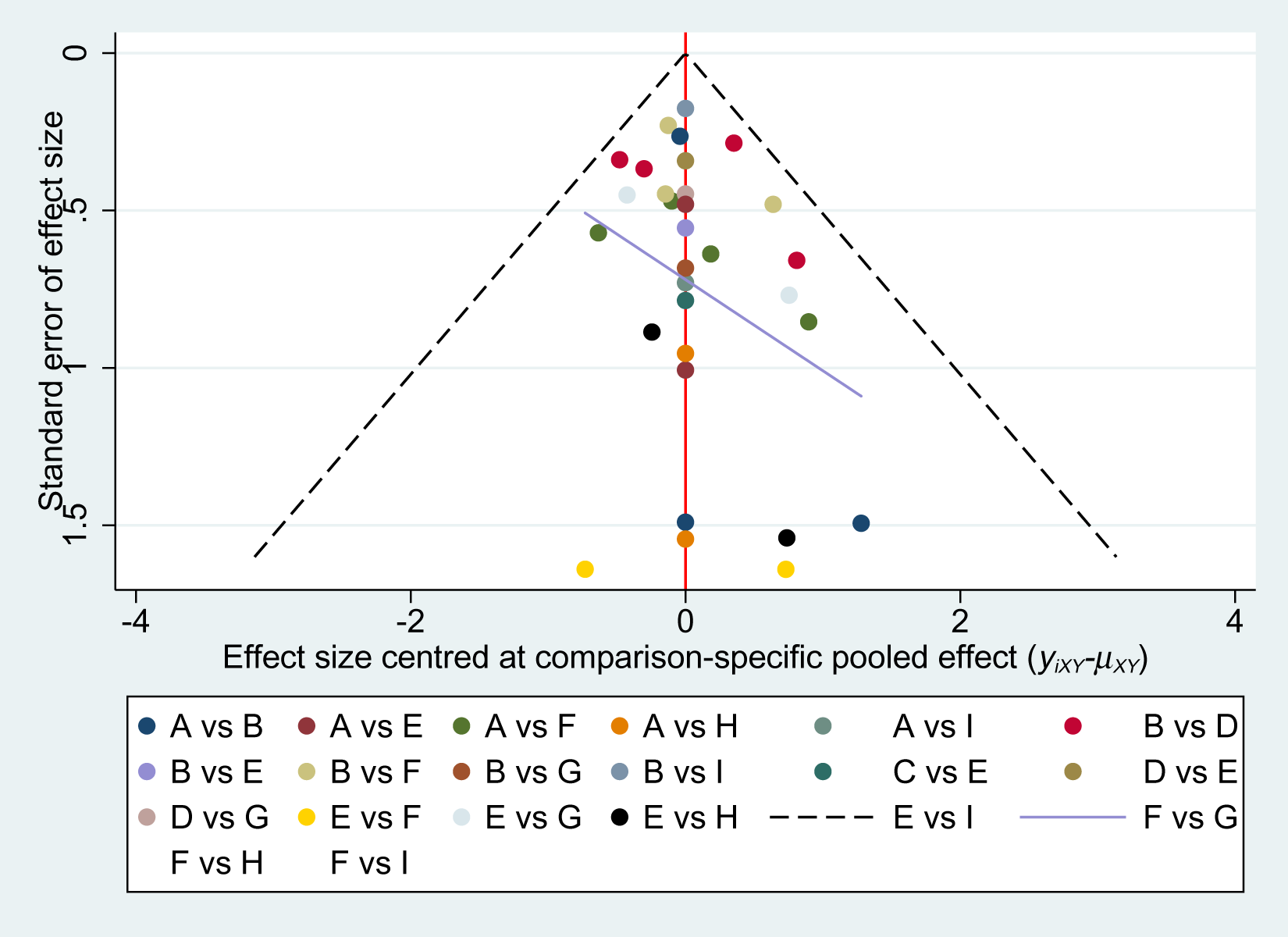


**Figure S26** Funnel plot for Decrease in potassium analysis


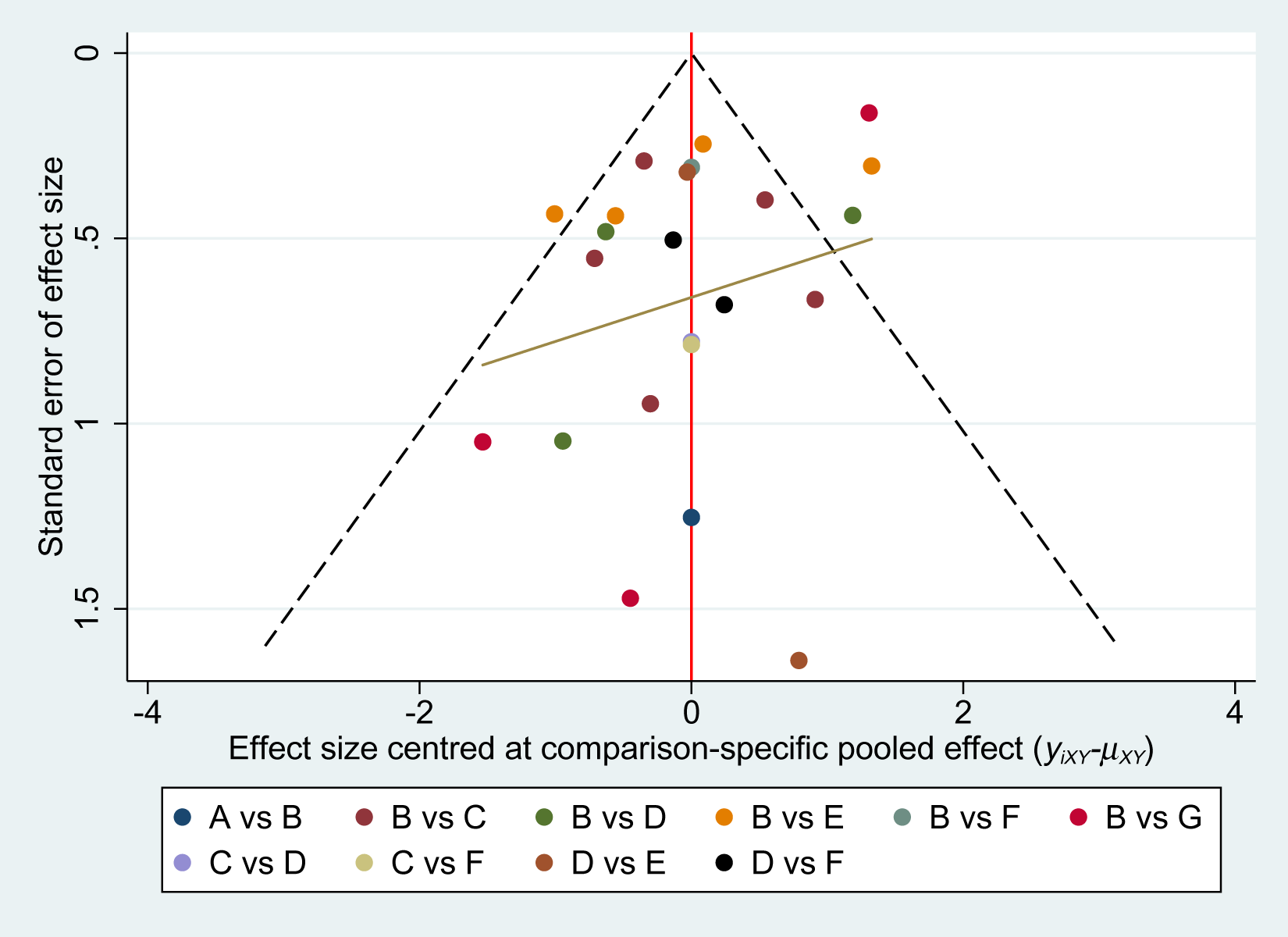


**Figure S27** Funnel plot for Increase in total or direct bilirubin analysis


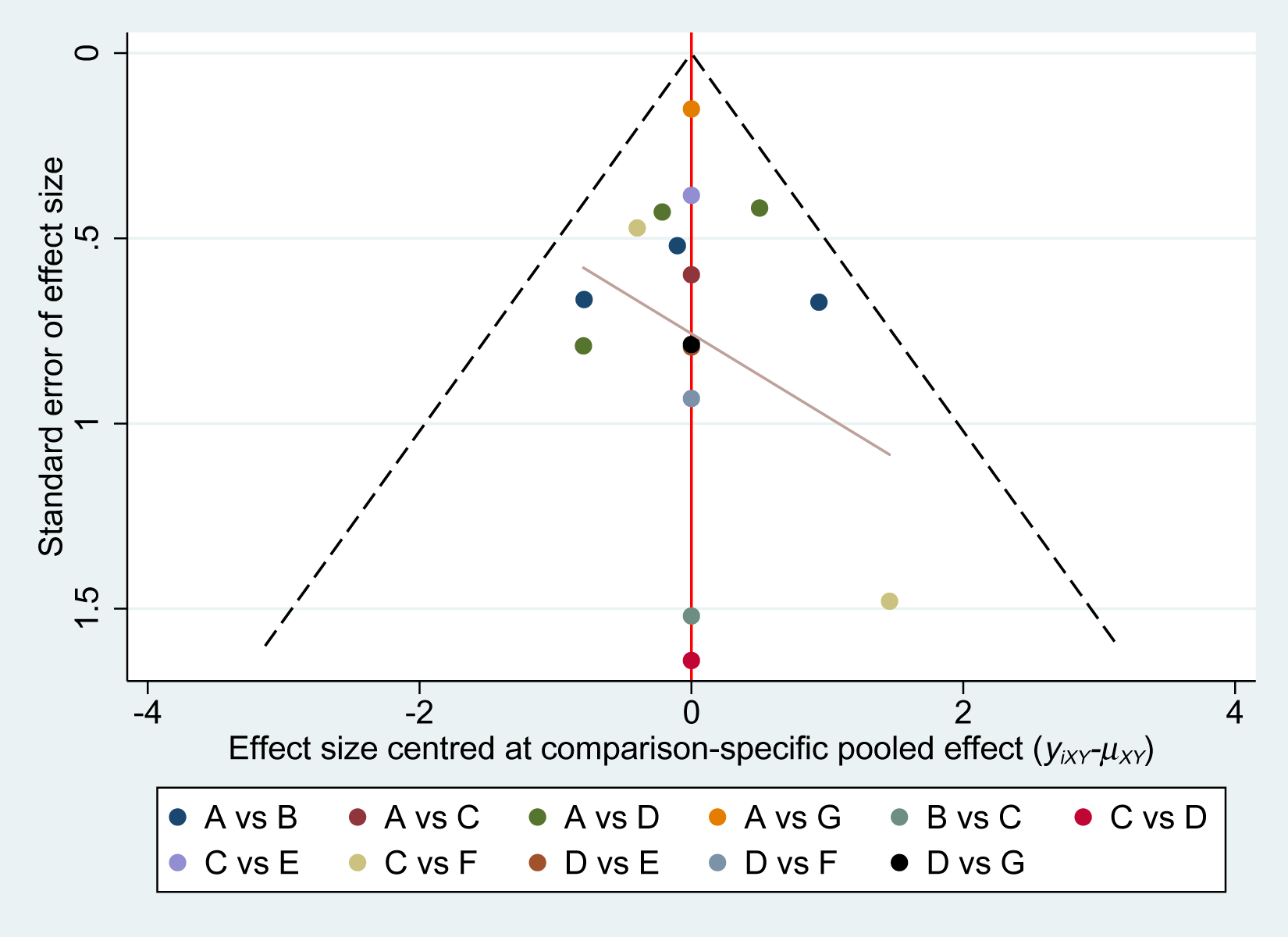


**Figure S28** Funnel plot for Increase in creatinine analysis


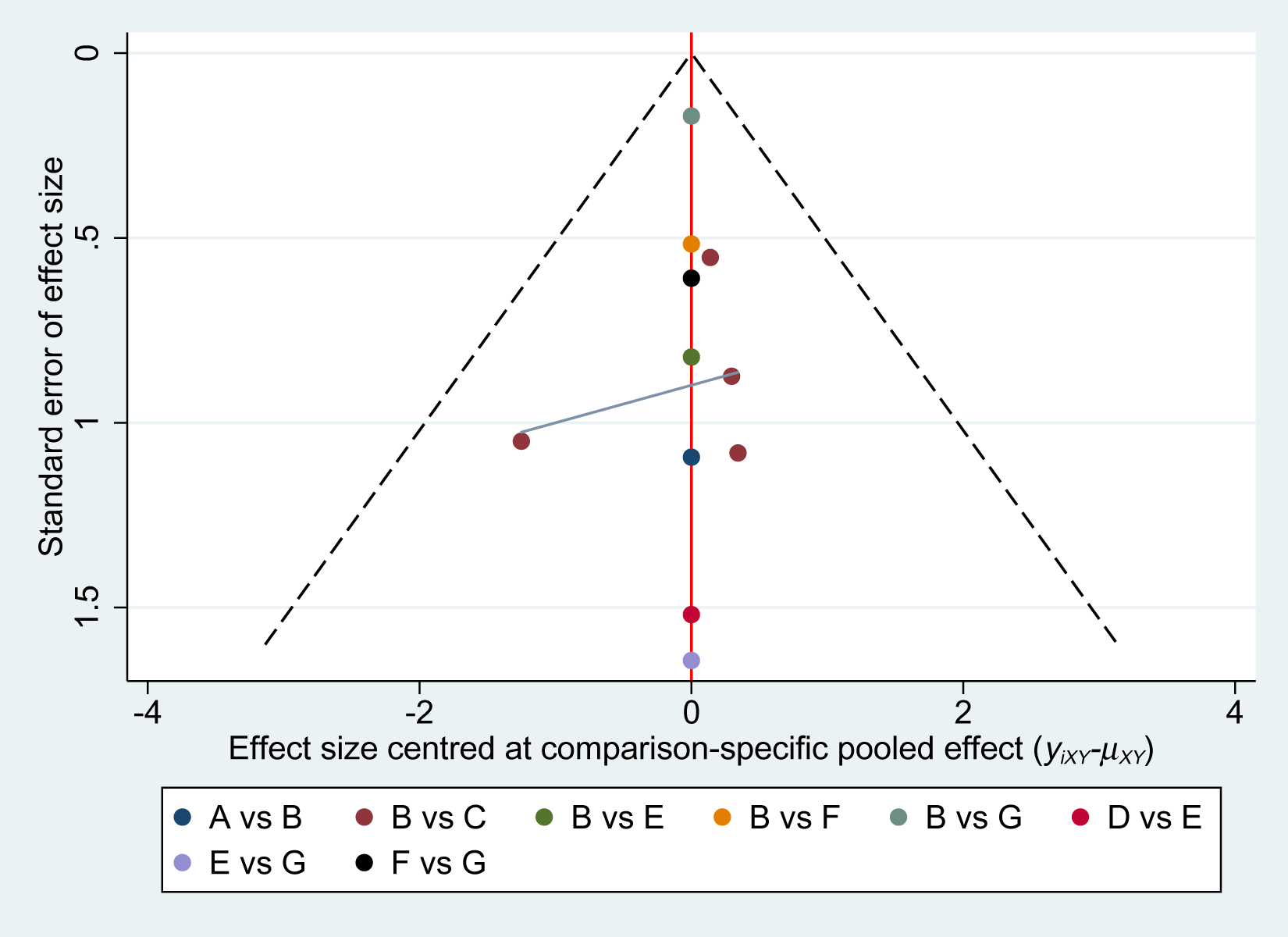


**Figure S29** Funnel plot for tolerability analysis


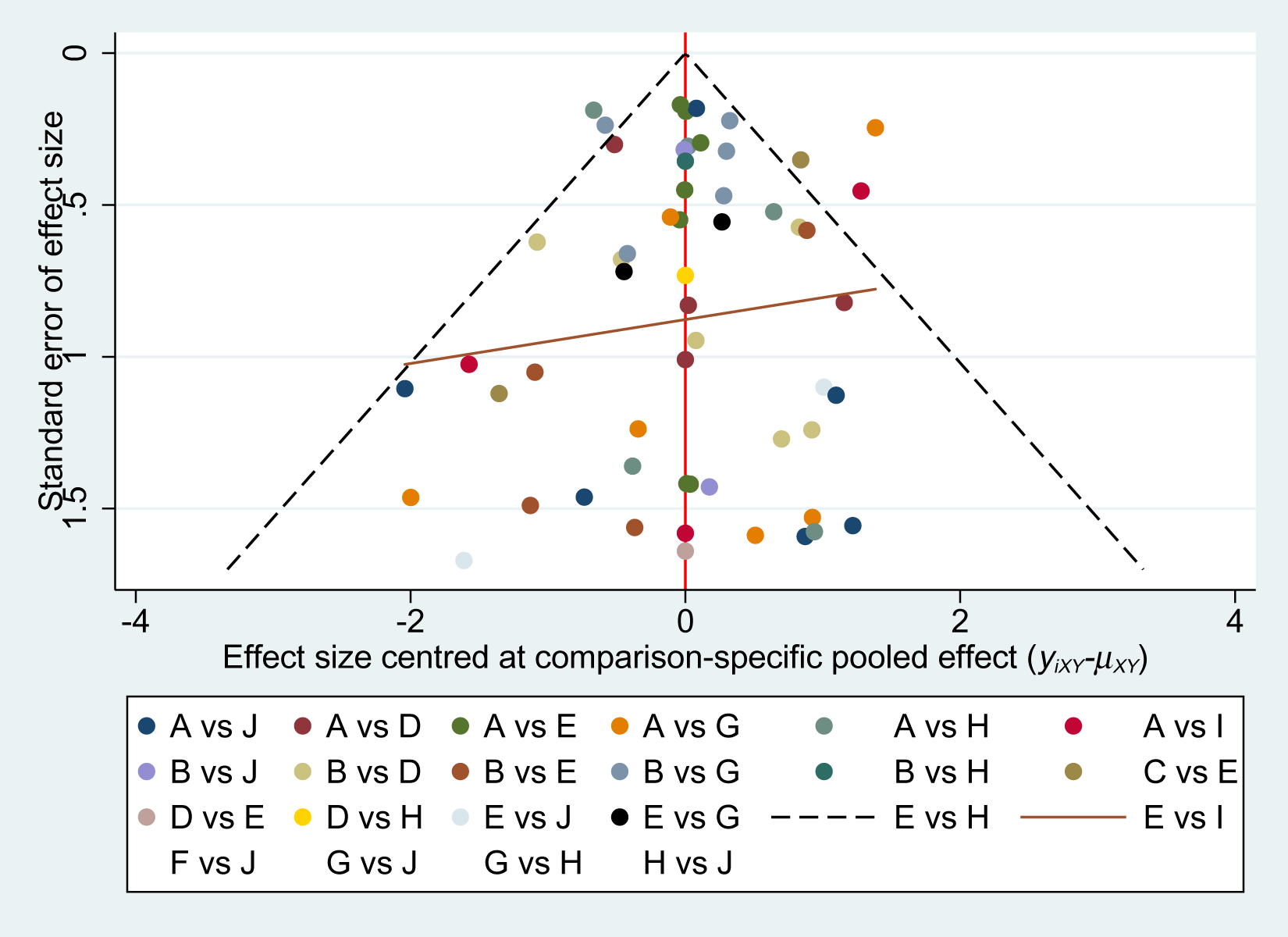


**References**

Aaron, S.D., Vandemheen, K.L., Freitag, A., Pedder, L., Cameron, W., Lavoie, A., et al. (2012). Treatment of Aspergillus fumigatus in patients with cystic fibrosis: a randomized, placebo-controlled pilot study. PLoS One 7, e36077. doi:10.1371/journal.pone.0036077

Ally, R., Schurmann, D., Kreisel, W., Carosi, G., Aguirrebengoa, K., Dupont, B., et al. (2001). A randomized, double-blind, double-dummy, multicenter trial of voriconazole and fluconazole in the treatment of esophageal candidiasis in immunocompromised patients. Clin Infect Dis 33, 1447-1454. doi:10.1086/322653

Arathoon, E.G., Gotuzzo, E., Noriega, L.M., Berman, R.S., DiNubile, M.J., Sable, C.A. (2002). Randomized, double-blind, multicenter study of caspofungin versus amphotericin B for treatment of oropharyngeal and esophageal candidiases. Antimicrob Agents Chemother 46, 451-457. doi:10.1128/aac.46.2.451-457.2002

Boogaerts, M., Maertens, J., van Hoof, A., de Bock, R., Fillet, G., Peetermans, M., et al. (2001a). Itraconazole versus amphotericin B plus nystatin in the prophylaxis of fungal infections in neutropenic cancer patients. J Antimicrob Chemother 48, 97-103. doi:10.1093/jac/48.1.97

Boogaerts, M., Winston, D.J., Bow, E.J., Garber, G., Reboli, A.C., Schwarer, A.P., et al. (2001b). Intravenous and oral itraconazole versus intravenous amphotericin B deoxycholate as empirical antifungal therapy for persistent fever in neutropenic patients with cancer who are receiving broad-spectrum antibacterial therapy. A randomized, controlled trial. Ann Intern Med 135, 412-422. doi:10.7326/0003-4819-135-6-200109180-00010

Chariyalertsak, S., Supparatpinyo, K., Sirisanthana, T., Nelson, K.E. (2002). A controlled trial of itraconazole as primary prophylaxis for systemic fungal infections in patients with advanced human immunodeficiency virus infection in Thailand. Clin Infect Dis 34, 277-284. doi:10.1086/338154

Cornely, O.A., Maertens, J., Winston, D.J., Perfect, J., Ullmann, A.J., Walsh, T.J., et al. (2007). Posaconazole vs. fluconazole or itraconazole prophylaxis in patients with neutropenia. N Engl J Med 356, 348-359. doi:10.1056/NEJMoa061094

Cornely, O.A., Meems, L., Herbrecht, R., Viscoli, C., van Amsterdam, R.G., Ruhnke, M. (2015). Randomised, multicentre trial of micafungin vs. an institutional standard regimen for salvage treatment of invasive aspergillosis. Mycoses 58, 58-64. doi:10.1111/myc.12274

Corvò, R., Amichetti, M., Ascarelli, A., Arcangeli, G., Buffoli, A., Cellini, N., et al. (2008). Effects of fluconazole in the prophylaxis of oropharyngeal candidiasis in patients undergoing radiotherapy for head and neck tumour: results from a double-blind placebo-controlled trial. Eur J Cancer Care (Engl) 17, 270-277. doi:10.1111/j.1365-2354.2007.00855.x

de Wet, N., Llanos-Cuentas, A., Suleiman, J., Baraldi, E., Krantz, E.F., Della Negra, M., et al. (2004). A randomized, double-blind, parallel-group, dose-response study of micafungin compared with fluconazole for the treatment of esophageal candidiasis in HIV-positive patients. Clin Infect Dis 39, 842-849. doi:10.1086/423377

de Wet, N.T., Bester, A.J., Viljoen, J.J., Filho, F., Suleiman, J.M., Ticona, E., et al. (2005). A randomized, double blind, comparative trial of micafungin (FK463) vs. fluconazole for the treatment of oesophageal candidiasis. Aliment Pharmacol Ther 21, 899-907. doi:10.1111/j.1365-2036.2005.02427.x

Dowell, J.A., Schranz, J., Baruch, A., Foster, G. (2005). Safety and pharmacokinetics of coadministered voriconazole and anidulafungin. J Clin Pharmacol 45, 1373-1382. doi:10.1177/0091270005281234

Galgiani, J.N., Catanzaro, A., Cloud, G.A., Johnson, R.H., Williams, P.L., Mirels, L.F., et al. (2000). Comparison of oral fluconazole and itraconazole for progressive, nonmeningeal coccidioidomycosis. A randomized, double-blind trial. Mycoses Study Group. Ann Intern Med 133, 676-686. doi:10.7326/0003-4819-133-9-200011070-00009

Gallin, J.I., Alling, D.W., Malech, H.L., Wesley, R., Koziol, D., Marciano, B., et al. (2003). Itraconazole to prevent fungal infections in chronic granulomatous disease. N Engl J Med 348, 2416-2422. doi:10.1056/NEJMoa021931

Glasmacher, A., Cornely, O., Ullmann, A.J., Wedding, U., Bodenstein, H., Wandt, H., et al. (2006). An open-label randomized trial comparing itraconazole oral solution with fluconazole oral solution for primary prophylaxis of fungal infections in patients with haematological malignancy and profound neutropenia. J Antimicrob Chemother 57, 317-325. doi:10.1093/jac/dki440

Groll, A.H., Silling, G., Young, C., Schwerdtfeger, R., Ostermann, H., Heinz, W.J., et al. (2010). Randomized comparison of safety and pharmacokinetics of caspofungin, liposomal amphotericin B, and the combination of both in allogeneic hematopoietic stem cell recipients. Antimicrob Agents Chemother 54, 4143-4149. doi:10.1128/aac.00425-10

Harousseau, J.L., Dekker, A.W., Stamatoullas-Bastard, A., Fassas, A., Linkesch, W., Gouveia, J., et al. (2000). Itraconazole oral solution for primary prophylaxis of fungal infections in patients with hematological malignancy and profound neutropenia: a randomized, double-blind, double-placebo, multicenter trial comparing itraconazole and amphotericin B. Antimicrob Agents Chemother 44, 1887-1893. doi:10.1128/aac.44.7.1887-1893.2000

Herbrecht, R., Denning, D.W., Patterson, T.F., Bennett, J.E., Greene, R.E., Oestmann, J.W., et al. (2002). Voriconazole versus amphotericin B for primary therapy of invasive aspergillosis. N Engl J Med 347, 408-415. doi:10.1056/NEJMoa020191

Hiramatsu, Y., Maeda, Y., Fujii, N., Saito, T., Nawa, Y., Hara, M., et al. (2008). Use of micafungin versus fluconazole for antifungal prophylaxis in neutropenic patients receiving hematopoietic stem cell transplantation. Int J Hematol 88, 588-595. doi:10.1007/s12185-008-0196-y

Huang, X., Chen, H., Han, M., Zou, P., Wu, D., Lai, Y., et al. (2012). Multicenter, randomized, open-label study comparing the efficacy and safety of micafungin versus itraconazole for prophylaxis of invasive fungal infections in patients undergoing hematopoietic stem cell transplant. Biol Blood Marrow Transplant 18, 1509-1516. doi:10.1016/j.bbmt.2012.03.014

Ito, Y., Ohyashiki, K., Yoshida, I., Takeuchi, M., Aoyama, Y., Mugitani, A., et al. (2007). The prophylactic effect of itraconazole capsules and fluconazole capsules for systemic fungal infections in patients with acute myeloid leukemia and myelodysplastic syndromes: a Japanese multicenter randomized, controlled study. Int J Hematol 85, 121-127. doi:10.1532/ijh97.06079

Jarvis, J.N., Leeme, T.B., Molefi, M., Chofle, A.A., Bidwell, G., Tsholo, K., et al. (2019). Short-course High-dose Liposomal Amphotericin B for Human Immunodeficiency Virus-associated Cryptococcal Meningitis: A Phase 2 Randomized Controlled Trial. Clin Infect Dis 68, 393-401. doi:10.1093/cid/ciy515

Jeong, S.H., Kim, D.Y., Jang, J.H., Mun, Y.C., Choi, C.W., Kim, S.H., et al. (2016). Efficacy and safety of micafungin versus intravenous itraconazole as empirical antifungal therapy for febrile neutropenic patients with hematological malignancies: a randomized, controlled, prospective, multicenter study. Ann Hematol 95, 337-344. doi:10.1007/s00277-015-2545-2

Kakuda, T.N., Van Solingen-Ristea, R., Aharchi, F., Smedt, G.D., Witek, J., Nijs, S., et al. (2013). Pharmacokinetics and short-term safety of etravirine in combination with fluconazole or voriconazole in HIV-negative volunteers. J Clin Pharmacol 53, 41-50. doi:10.1177/0091270011433329

Keirns, J., Desai, A., Kowalski, D., Lademacher, C., Mujais, S., Parker, B., et al. (2017). QT Interval Shortening With Isavuconazole: In Vitro and In Vivo Effects on Cardiac Repolarization. Clin Pharmacol Ther 101, 782-790. doi:10.1002/cpt.620

Kohno, S., Izumikawa, K., Ogawa, K., Kurashima, A., Okimoto, N., Amitani, R., et al. (2010). Intravenous micafungin versus voriconazole for chronic pulmonary aspergillosis: a multicenter trial in Japan. J Infect 61, 410-418. doi:10.1016/j.jinf.2010.08.005

Kohno, S., Izumikawa, K., Yoshida, M., Takesue, Y., Oka, S., Kamei, K., et al. (2013). A double-blind comparative study of the safety and efficacy of caspofungin versus micafungin in the treatment of candidiasis and aspergillosis. Eur J Clin Microbiol Infect Dis 32, 387-397. doi:10.1007/s10096-012-1754-z

Krause, D.S., Simjee, A.E., van Rensburg, C., Viljoen, J., Walsh, T.J., Goldstein, B.P., et al. (2004). A randomized, double-blind trial of anidulafungin versus fluconazole for the treatment of esophageal candidiasis. Clin Infect Dis 39, 770-775. doi:10.1086/423378

Krishna, G., Ma, L., Martinho, M., Preston, R.A., O'Mara, E. (2012). A new solid oral tablet formulation of posaconazole: a randomized clinical trial to investigate rising single- and multiple-dose pharmacokinetics and safety in healthy volunteers. J Antimicrob Chemother 67, 2725-2730. doi:10.1093/jac/dks268

Kuse, E.R., Chetchotisakd, P., da Cunha, C.A., Ruhnke, M., Barrios, C., Raghunadharao, D., et al. (2007). Micafungin versus liposomal amphotericin B for candidaemia and invasive candidosis: a phase III randomised double-blind trial. Lancet 369, 1519-1527. doi:10.1016/s0140-6736(07)60605-9

Le, T., Kinh, N.V., Cuc, N.T.K., Tung, N.L.N., Lam, N.T., Thuy, P.T.T., et al. (2017). A Trial of Itraconazole or Amphotericin B for HIV-Associated Talaromycosis. N Engl J Med 376, 2329-2340. doi:10.1056/NEJMoa1613306

Lefebvre, J.L., Domenge, C. (2002). A comparative study of the efficacy and safety of fluconazole oral suspension and amphotericin B oral suspension in cancer patients with mucositis. Oral Oncol 38, 337-342. doi:10.1016/s1368-8375(01)00063-x

Liu, P., Foster, G., Gandelman, K., LaBadie, R.R., Allison, M.J., Gutierrez, M.J., et al. (2007). Steady-state pharmacokinetic and safety profiles of voriconazole and ritonavir in healthy male subjects. Antimicrob Agents Chemother 51, 3617-3626. doi:10.1128/aac.00526-07

Maertens, J.A., Madero, L., Reilly, A.F., Lehrnbecher, T., Groll, A.H., Jafri, H.S., et al. (2010). A randomized, double-blind, multicenter study of caspofungin versus liposomal amphotericin B for empiric antifungal therapy in pediatric patients with persistent fever and neutropenia. Pediatr Infect Dis J 29, 415-420. doi:10.1097/INF.0b013e3181da2171

Maertens, J.A., Raad, II, Marr, K.A., Patterson, T.F., Kontoyiannis, D.P., Cornely, O.A., et al. (2016). Isavuconazole versus voriconazole for primary treatment of invasive mould disease caused by Aspergillus and other filamentous fungi (SECURE): a phase 3, randomised-controlled, non-inferiority trial. Lancet 387, 760-769. doi:10.1016/s0140-6736(15)01159-9

Malik, I.A., Moid, I., Aziz, Z., Khan, S., Suleman, M. (1998). A randomized comparison of fluconazole with amphotericin B as empiric anti-fungal agents in cancer patients with prolonged fever and neutropenia. Am J Med 105, 478-483. doi:10.1016/s0002-9343(98)00326-x

Mandhaniya, S., Swaroop, C., Thulkar, S., Vishnubhatla, S., Kabra, S.K., Xess, I., et al. (2011). Oral voriconazole versus intravenous low dose amphotericin B for primary antifungal prophylaxis in pediatric acute leukemia induction: a prospective, randomized, clinical study. J Pediatr Hematol Oncol 33, e333-341. doi:10.1097/MPH.0b013e3182331bc7

Marks, D.I., Pagliuca, A., Kibbler, C.C., Glasmacher, A., Heussel, C.P., Kantecki, M., et al. (2011). Voriconazole versus itraconazole for antifungal prophylaxis following allogeneic haematopoietic stem-cell transplantation. Br J Haematol 155, 318-327. doi:10.1111/j.1365-2141.2011.08838.x

Mattiuzzi, G.N., Cortes, J., Alvarado, G., Verstovsek, S., Koller, C., Pierce, S., et al. (2011). Efficacy and safety of intravenous voriconazole and intravenous itraconazole for antifungal prophylaxis in patients with acute myelogenous leukemia or high-risk myelodysplastic syndrome. Support Care Cancer 19, 19-26. doi:10.1007/s00520-009-0783-3

McKinsey, D.S., Wheat, L.J., Cloud, G.A., Pierce, M., Black, J.R., Bamberger, D.M., et al. (1999). Itraconazole prophylaxis for fungal infections in patients with advanced human immunodeficiency virus infection: randomized, placebo-controlled, double-blind study. National Institute of Allergy and Infectious Diseases Mycoses Study Group. Clin Infect Dis 28, 1049-1056. doi:10.1086/514744

Migoya, E.M., Mistry, G.C., Stone, J.A., Comisar, W., Sun, P., Norcross, A., et al. (2011). Safety and pharmacokinetics of higher doses of caspofungin in healthy adult participants. J Clin Pharmacol 51, 202-211. doi:10.1177/0091270010374853

Mora-Duarte, J., Betts, R., Rotstein, C., Colombo, A.L., Thompson-Moya, L., Smietana, J., et al. (2002). Comparison of caspofungin and amphotericin B for invasive candidiasis. N Engl J Med 347, 2020-2029. doi:10.1056/NEJMoa021585

Ostrosky-Zeichner, L., Shoham, S., Vazquez, J., Reboli, A., Betts, R., Barron, M.A., et al. (2014). MSG-01: A randomized, double-blind, placebo-controlled trial of caspofungin prophylaxis followed by preemptive therapy for invasive candidiasis in high-risk adults in the critical care setting. Clin Infect Dis 58, 1219-1226. doi:10.1093/cid/ciu074

Oyake, T., Kowata, S., Murai, K., Ito, S., Akagi, T., Kubo, K., et al. (2016). Comparison of micafungin and voriconazole as empirical antifungal therapies in febrile neutropenic patients with hematological disorders: a randomized controlled trial. Eur J Haematol 96, 602-609. doi:10.1111/ejh.12641

Park, S., Choi, S.-M., Lee, D.-G., Choi, J.-H., Yoo, J.-H., Min, W.-S., et al., 2006. Intravenous itraconazole vs. amphotericin B deoxycholate for empirical antifungal therapy in patients with persistent neutropenic fever, The korean journal of internal medicine, pp. 165‐172.

Parkes-Ratanshi, R., Wakeham, K., Levin, J., Namusoke, D., Whitworth, J., Coutinho, A., et al. (2011). Primary prophylaxis of cryptococcal disease with fluconazole in HIV-positive Ugandan adults: a double-blind, randomised, placebo-controlled trial. Lancet Infect Dis 11, 933-941. doi:10.1016/s1473-3099(11)70245-6

Purkins, L., Wood, N., Greenhalgh, K., Allen, M.J., Oliver, S.D. (2003a). Voriconazole, a novel wide-spectrum triazole: oral pharmacokinetics and safety. Br J Clin Pharmacol 56 Suppl 1, 10-16. doi:10.1046/j.1365-2125.2003.01993.x

Purkins, L., Wood, N., Kleinermans, D., Nichols, D. (2003b). Voriconazole potentiates warfarin-induced prothrombin time prolongation. Br J Clin Pharmacol 56 Suppl 1, 24-29. doi:10.1046/j.1365-2125.2003.01995.x

Queiroz-Telles, F., Goldani, L.Z., Schlamm, H.T., Goodrich, J.M., Espinel-Ingroff, A., Shikanai-Yasuda, M.A. (2007). An open-label comparative pilot study of oral voriconazole and itraconazole for long-term treatment of paracoccidioidomycosis. Clin Infect Dis 45, 1462-1469. doi:10.1086/522973

Reboli, A.C., Rotstein, C., Pappas, P.G., Chapman, S.W., Kett, D.H., Kumar, D., et al. (2007). Anidulafungin versus fluconazole for invasive candidiasis. N Engl J Med 356, 2472-2482. doi:10.1056/NEJMoa066906

Rotstein, C., Bow, E.J., Laverdiere, M., Ioannou, S., Carr, D., Moghaddam, N. (1999). Randomized placebo-controlled trial of fluconazole prophylaxis for neutropenic cancer patients: benefit based on purpose and intensity of cytotoxic therapy. The Canadian Fluconazole Prophylaxis Study Group. Clin Infect Dis 28, 331-340. doi:10.1086/515128

Saliba, F., Pascher, A., Cointault, O., Laterre, P.F., Cervera, C., De Waele, J.J., et al. (2015). Randomized trial of micafungin for the prevention of invasive fungal infection in high-risk liver transplant recipients. Clin Infect Dis 60, 997-1006. doi:10.1093/cid/ciu1128

Schuster, M.G., Edwards, J.E., Jr., Sobel, J.D., Darouiche, R.O., Karchmer, A.W., Hadley, S., et al. (2008). Empirical fluconazole versus placebo for intensive care unit patients: a randomized trial. Ann Intern Med 149, 83-90. doi:10.7326/0003-4819-149-2-200807150-00004

Shang, W., Feng, G., Sun, R., Wang, X., Liu, W., Zhang, S., et al. (2012). Comparison of micafungin and voriconazole in the treatment of invasive fungal infections in kidney transplant recipients. J Clin Pharm Ther 37, 652-656. doi:10.1111/j.1365-2710.2012.01362.x

Sobel, J.D., Wiesenfeld, H.C., Martens, M., Danna, P., Hooton, T.M., Rompalo, A., et al. (2004). Maintenance fluconazole therapy for recurrent vulvovaginal candidiasis. N Engl J Med 351, 876-883. doi:10.1056/NEJMoa033114

Taillandier, J., Esnault, Y., Alemanni, M. (2000). A comparison of fluconazole oral suspension and amphotericin B oral suspension in older patients with oropharyngeal candidosis. Multicentre Study Group. Age Ageing 29, 117-123. doi:10.1093/ageing/29.2.117

Ullmann, A.J., Lipton, J.H., Vesole, D.H., Chandrasekar, P., Langston, A., Tarantolo, S.R., et al. (2007). Posaconazole or fluconazole for prophylaxis in severe graft-versus-host disease. N Engl J Med 356, 335-347. doi:10.1056/NEJMoa061098

van Burik, J.A., Ratanatharathorn, V., Stepan, D.E., Miller, C.B., Lipton, J.H., Vesole, D.H., et al. (2004). Micafungin versus fluconazole for prophylaxis against invasive fungal infections during neutropenia in patients undergoing hematopoietic stem cell transplantation. Clin Infect Dis 39, 1407-1416. doi:10.1086/422312

Vazquez, J.A., Skiest, D.J., Nieto, L., Northland, R., Sanne, I., Gogate, J., et al. (2006). A multicenter randomized trial evaluating posaconazole versus fluconazole for the treatment of oropharyngeal candidiasis in subjects with HIV/AIDS. Clin Infect Dis 42, 1179-1186. doi:10.1086/501457

Villanueva, A., Arathoon, E.G., Gotuzzo, E., Berman, R.S., DiNubile, M.J., Sable, C.A. (2001). A randomized double-blind study of caspofungin versus amphotericin for the treatment of candidal esophagitis. Clin Infect Dis 33, 1529-1535. doi:10.1086/323401

Villanueva, A., Gotuzzo, E., Arathoon, E.G., Noriega, L.M., Kartsonis, N.A., Lupinacci, R.J., et al. (2002). A randomized double-blind study of caspofungin versus fluconazole for the treatment of esophageal candidiasis. Am J Med 113, 294-299. doi:10.1016/s0002-9343(02)01191-9

Viscoli, C., Castagnola, E., Van, L.M., Moroni, C., Garaventa, A., Rossi, M., et al., 1996. Fluconazole versus amphotericin B as empirical antifungal therapy of unexplained fever in granulocytopenic cancer patients: a pragmatic, multicentre, prospective and randomised clinical trial, European journal of cancer (Oxford, England : 1990), pp. 814‐820.

Walsh, T.J., Pappas, P., Winston, D.J., Lazarus, H.M., Petersen, F., Raffalli, J., et al. (2002). Voriconazole compared with liposomal amphotericin B for empirical antifungal therapy in patients with neutropenia and persistent fever. N Engl J Med 346, 225-234. doi:10.1056/nejm200201243460403

Walsh, T.J., Teppler, H., Donowitz, G.R., Maertens, J.A., Baden, L.R., Dmoszynska, A., et al. (2004). Caspofungin versus liposomal amphotericin B for empirical antifungal therapy in patients with persistent fever and neutropenia. N Engl J Med 351, 1391-1402. doi:10.1056/NEJMoa040446

Winston, D.J., Busuttil, R.W. (2002). Randomized controlled trial of oral itraconazole solution versus intravenous/oral fluconazole for prevention of fungal infections in liver transplant recipients. Transplantation 74, 688-695. doi:10.1097/00007890-200209150-00017

Winston, D.J., Hathorn, J.W., Schuster, M.G., Schiller, G.J., Territo, M.C. (2000). A multicenter, randomized trial of fluconazole versus amphotericin B for empiric antifungal therapy of febrile neutropenic patients with cancer. Am J Med 108, 282-289. doi:10.1016/s0002-9343(99)00457-x
